# Supplementary material for: Discrepancy Study of the Chemical Constituents of Panax Ginseng from Different Growth Environments with UPLC-MS-Based Metabolomics Strategy
Source: Molecules. 2023 Mar 24;28(7):2928. doi: 10.3390/molecules28072928 (PMC10095802; doi:10.3390/molecules28072928)
Supplement: Supplementary file 1 [file molecules-28-02928-s001.zip › molecules-2301062-supplementary.pdf]

Figure S1

Aglycone, glycosyl and acyls types of the ginsenosides. (**A**) The aglycone of all ginsenosides (PPD1 is protopanaxadiol saponin, PPT1 is protopanaxatriol saponin, OA is oleanolic acid saponin, PG is pseudo ginsenoside, and the rest are aglycones with changed side chains). (**B**) The glycosyl and acyls of all ginsenosides.

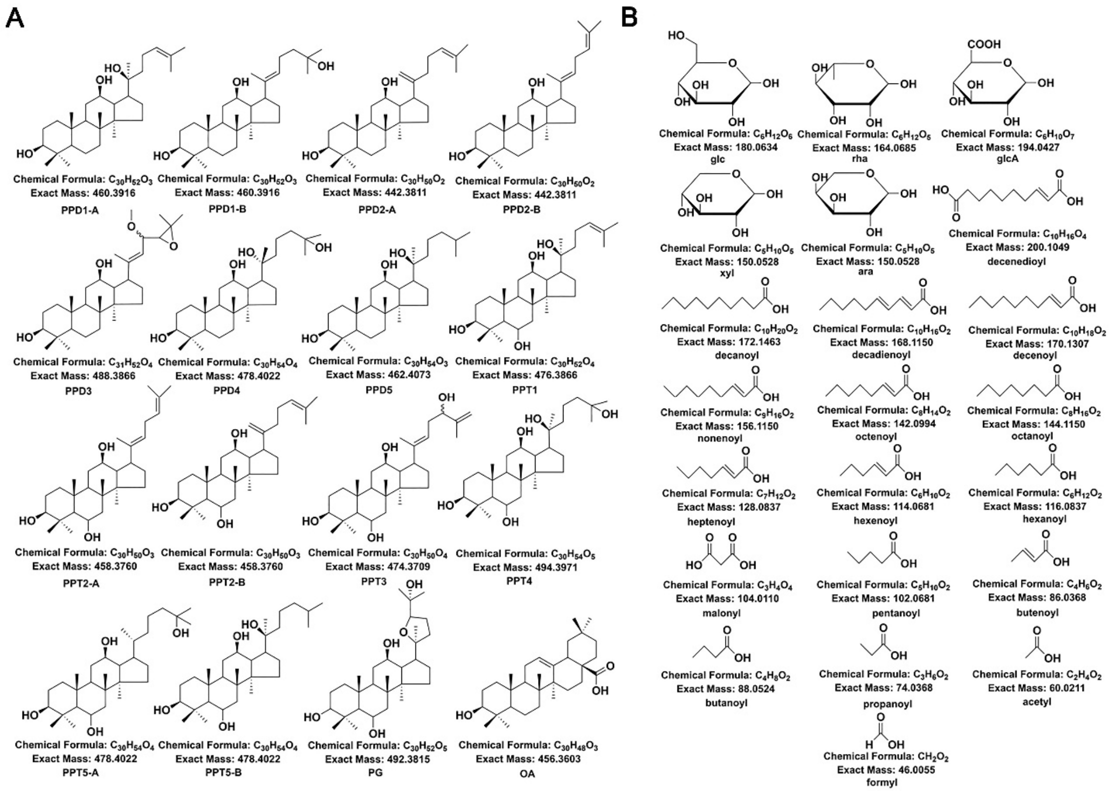

Figure S2

The histogram of labeled reference content in four groups of *P. ginseng* samples.

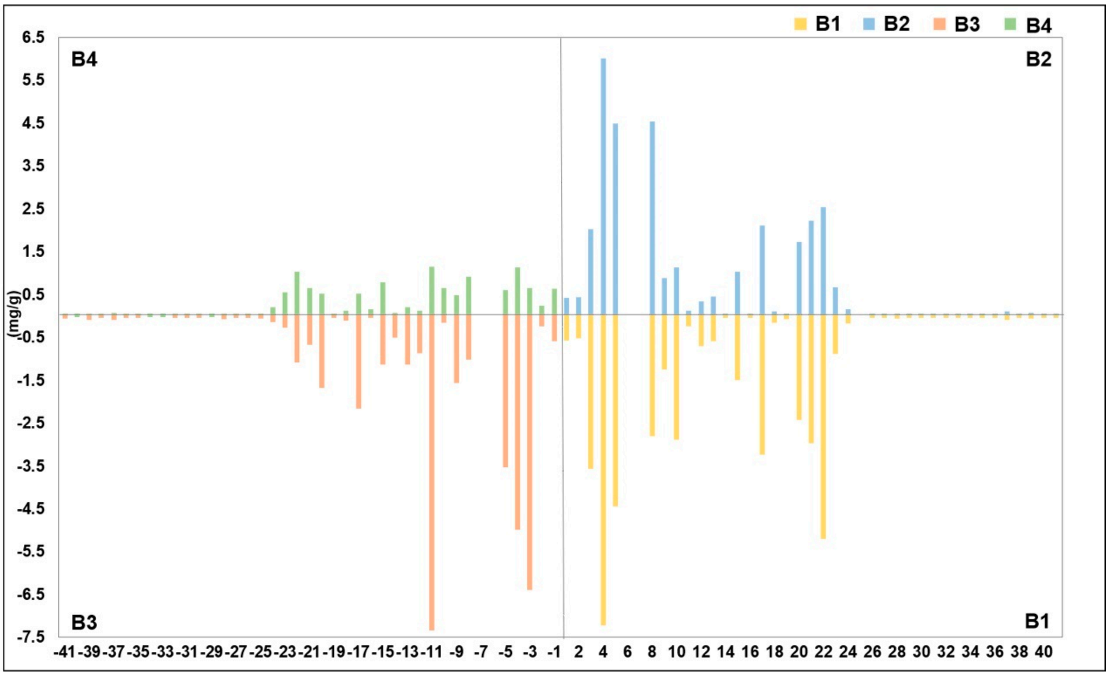

Figure S3

MRM mass spectrograms of each group of samples

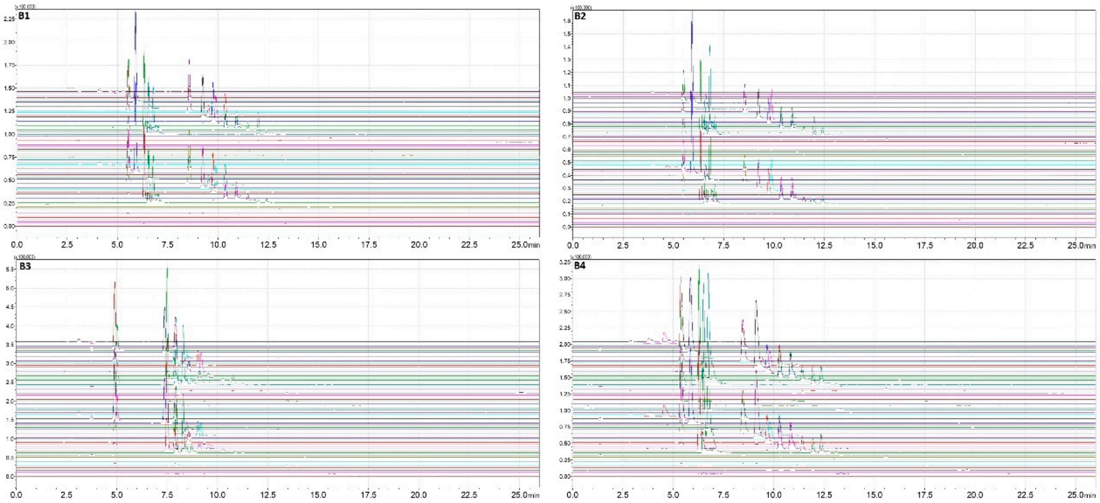

Table S1

MS and MS/MS fragmentation of the 81 new ginsenosides.

No.	Rt	Found At Mass	MS/MS Fragmentation	Identification	Name	Formula	Theoretical Mass (Da)	Source	Ppm
1	1.32	695.4369	695.4409[M-H]⁻ 653.4249[M-H-Ac]⁻ 635.4196[M-H-Ac-H2O]⁻ 491.3773[M-H-Ac-glc]⁻	PG-glc-acetyl	acetyl-PG-RT5A	C38H64O11	696.4449	B1, B2, B3, B4	0.29
2	1.39	975.5436	975.5508[M-H]⁻ 933.5478[M-H-Ac]⁻ 801.4520[M-H-Ac-xyl/ara]⁻ 639.4764[M-H-Ac-xyl/ara-glc]⁻ 477.3725[M-H-Ac-xyl/ara-2glc]⁻	PPD4 /PPT5-glc-glc-xyl/ara-acetyl	H-acetyl-NG-R1A	C49H84O19	976.5607	B1, B2, B3	9.53
3	1.62	843.503	843.5047[M-H]⁻ 801.4975[M-H-Ac]⁻ 639.4474[M-H-Ac-glc]⁻ 477.3917[M-H-Ac-2glc]⁻	PPD4/PPT5-glc-glc-acetyl	H-acetyl-Rg1A	C44H76O15	844.5184	B1, B3	9.01
4	1.68	695.4434	695.4434[M-H]⁻ 653.4506[M-H-Ac]⁻ 635.4248[M-H-Ac-H2O]⁻ 491.3734[M-H-Ac-glc]⁻	PG-glc-acetyl	acetyl-PG-RT5B	C38H64O11	696.4449	B2, B3, B4	-9.06
5	1.87	887.4938	887.5044[M-H]⁻ 843.5059[M-H-CO2]⁻ 801.4959[M-H-CO2-Ac]⁻ 639.4376[M-H-CO2-Ac-glc]⁻ 477.3861[M-H-CO2-Ac-2glc]⁻	PPD4/PPT5-glc-glc-malonyl	H-malonyl-NG-R1	C45H76O17	888.5083	B1, B3	7.55
6	2.21	843.5069	843.5069[M-H]⁻ 801.5057[M-H-Ac]⁻ 639.4390[M-H-Ac-glc]⁻ 477.3710[M-H-Ac-2glc]⁻	PPD4/PPT5-glc-glc-acetyl	H-acetyl-Rg1B	C44H76O15	844.5184	B1, B3	4.39
7	2.41	927.4953	927.4953[M-H]- 783.4803 637.4358 475.4038	PPT1-glc-glc-malonyl-acetyl	malonyl-acetyl-Rg1C	C47H76O18	928.5032	B1, B2, B3, B4	0.11
8	3.38	883.5058	883.5058[M-H]⁻ 815.4911[M-H-butenoyl]⁻ 653.4365[M-H-butenoyl-glc]⁻ 491.3820[M-H-butenoyl-2glc]⁻	PG-glc-glc--butenoyl	butenoyl-PG-RT5-G	C46H76O16	884.5133	B3	-0.34
9	3.43	1063.5697	1063.5764[M-H]⁻ 931.5228[M-H-xyl/ara]⁻ 799.4954[M-H-2xyl/ara]⁻ 653.4053[M-H-2xyl/ara-rha]⁻ 491.3828[M-H-2xyl/ara-rha-glc]⁻	PG-glc-rha-xyl/ara-xyl/ara	PG-F11-X2	C52H88O22	1064.5767	B1, B3, B4	-0.75

10	3.88	855.4702	855.3721[M-H]⁻ 811.4421[M-H-CO2]⁻ 769.4736[M-H-CO2-Ac]⁻ 637.4198[M-H-CO2-Ac-xyl/ara]⁻ 475.3785[M-H-CO2-Ac-xyl/ara-glc]⁻	PPT1-glc-xyl/ara-malonyl	malonyl-NG-R2A	C44H72O16	856.482	B1, B3	4.68
11	4.52	855.476	811.4970[M-H-CO2]⁻ 769.4548[M-H-CO2-Ac]⁻ 607.4351[M-H-CO2-Ac-glc]⁻ 475.3802[M-H-CO2-Ac-glc-xyl/ara]⁻	PPT1-glc-xyl/ara-malonyl	malonyl-NG-R2B	C44H72O16	856.482	B3	-2.10
12	4.75	999.5567	999.5567[M-H]⁻ 931.5066[M-H-C4H4O]⁻ 799.4781[M-H-C4H4O-xyl/ara]⁻ 637.4363[M-H-C4H4O-xyl/ara-glc]⁻ 475.3695[M-H-C4H4O-xyl/ara-2glc]⁻	PPT1-glc-glc-xyl/ara-butenoyl	butenoyl-Re1	C51H84O19	1000.5607	B1, B3, B4	-3.80
13	5.03	925.5862	925.5862[M-H]⁻ 799.4620[M-H-octanoyl]⁻ 637.4310[M-H-octanoyl-glc]⁻ 475.3778[M-H-octanoyl-2glc]⁻	PPT1-glc-glc-octanoyl	octanoyl-Rg1	C50H86O15	926.5967	B3	2.92
14	5.29	887.4939	887.5211[M-H]⁻ 843.5072[M-H-CO2]⁻ 801.4906[M-H-CO2-Ac]⁻ 639.4408[M-H-CO2-Ac-glc]⁻ 477.3833[M-H-CO2-Ac-2glc]⁻	PPD4/PPT5-glc-glc-malonyl	H-malonyl-F2B	C45H76O17	888.5083	B3, B4	7.44
15	5.34	983.5583	983.5583[M-H]⁻ 939.4786[M-H-CO2]⁻ 841.5011[M-H-CO2-hexanoyl]⁻ 799.4868[M-H-CO2-hexanoyl-Ac]⁻ 637.4386[M-H-CO2-hexanoyl-Ac-glc]⁻ 475.3842[M-H-CO2-hexanoyl-Ac-2glc]⁻	PPT1-glc-glc-malonyl-hexanoyl	malonyl-hexanoyl-Rg1	C51H84O18	984.5658	B1, B3	-0.31
16	5.35	1279.5996	1191.6171[M-H-2CO2]⁻ 1149.6062[M-H-2CO2-Ac]⁻ 1131.5957[M-H-2CO2-Ac-H2O]⁻ 1107.5953[M-H-2CO2-2Ac]⁻ 945.5472[M-H-2CO2-2Ac-glc]⁻ 783.5033[M-H-2CO2-2Ac-2glc]⁻ 621.4567[M-H-2CO2-2Ac-3glc]⁻ 459.4026[M-H-2CO2-2Ac-4glc]⁻	PPD1-glc-glc-glc-glc-malonyl-malonyl	D-malonyl-Rb1	C60H96O29	1280.6037	B1, B2, B3, B4	-2.89
17	5.36	1193.5915	1193.5915 1149.6280 1107.6326 961.5373 799.4689 637.4350 475.3552	PPT1-glc-glc-glc-rha-malonyl	malonyl-Re-Ga	C57H94O26	1194.6033	B1, B3	3.35

18	5.66	1249.5866	1161.6069[M-H-2CO2]$^-$ 1119.5955[M-H-2CO2-Ac]$^-$ 1101.5865[M-H-2CO2-Ac-H2O]$^-$ 1077.5872[M-H-2CO2-2Ac]$^-$ 945.5560[M-H-2CO2-2Ac-xyl/ara]$^-$ 783.4834[M-H-2CO2-2Ac-xyl/ara-glc]$^-$ 621.4268[M-H-2CO2-2Ac-xyl/ara-2glc]$^-$ 459.3783[M-H-2CO2-2Ac-xyl/ara-3glc]$^-$	PPD1-glc-glc-glc-xyl/ara-malontl-malonyl	D-malonyl-Rc	C59H94O28	1250.5932	B1, B3	-0.96
19	5.74	1047.5349	1047.5365[M-H]$^-$ 1003.5476[M-H-CO2]$^-$ 961.5414[M-H-CO2-Ac]$^-$ 799.5008 [M-H-CO2-Ac-glc]$^-$ 637.4311[M-H-CO2-Ac-2glc]$^-$ 475.3544[M-H-CO2-Ac-3glc]$^-$	PPT1-glc-glc-glc-malonyl	malonyl-Re2-C	C51H84O22	1048.5454	B1, B2, B4	2.58
20	5.86	995.6385	995.5600[M+FA-H]$^-$ 949.5890[M-H]$^-$ 799.4851[M-H-decadienoyl]$^-$ 783.4906[M-H-decadienoyl-H2O]$^-$ 637.4335[M-H-decadienoyl-glc]$^-$ 475.3807[M-H-decadienoyl-2glc]$^-$	PPT1-2glc-decadienoyl	decadienoyl-Rg1	C52H86O15	950.5967	B2, B4	-0.11
21	5.98	1249.5851	1161.6082[M-H-2CO2]$^-$ 1119.5956[M-H-2CO2-Ac]$^-$ 1101.5883[M-H-2CO2-Ac-H2O]$^-$ 1077.5854[M-H-2CO2-2Ac]$^-$ 945.5282[M-H-2CO2-2Ac-xyl/ara]$^-$ 783.5170[M-H-2CO2-2Ac-xyl/ara-glc]$^-$ 621.4366[M-H-2CO2-2Ac-xyl/ara-2glc]$^-$ 459.3980[M-H-2CO2-2Ac-xyl/ara-3glc]$^-$	PPD1-glc-glc-glc-xyl/ara-malony-malonyl	D-malonyl-Rc	C59H94O28	1250.5932	B1, B2, B3, B4	0.24
22	6.03	971.5583	971.5583[M-H]$^-$ 929.5409[M-H-Ac]$^-$ 767.4929[M-H-Ac-glc]$^-$ 605.4216[M-H-Ac-2glc]$^-$ 459.3959[M-H-Ac-2glc-rha]$^-$	PPD1-glc-glc-rha-acetyl	acetyl-C-K-GR	C50H84O18	972.5658	B1, B3	-0.31
23	6.07	827.5129	827.5129[M-H] - 785.5054[M-H-Ac]$^-$ 767.4813[M-H-Ac-H2O]$^-$ 639.4435[M-H-Ac-rha]$^-$ 477.3872[M-H-Ac-octanoyl-glc]$^-$	PPD4/PPT5-glc-rha-acetyl	H-acetyl-Rg2	C44H76O14	828.5235	B3	3.38
24	6.46	855.4767	855.4801[M-H]$^-$ 811.4875[M-H-CO2]$^-$ 769.4719[M-H-CO2-Ac]$^-$ 637.4481[M-H-CO2-Ac-xyl/ara]$^-$ 475.3722[M-H-CO2-Ac-xyl/ara-glc]$^-$	PPT1-glc-xyl/ara-malonhyl	malonhyl-NG-R2	C44H72O16	856.482	B1, B2, B3, B4	-2.92

25	6.64	1291.6627	1193.6019[M-H-hexanoyl]⁻ 1149.6129[M-H-hexanoyl-CO2]⁻ 1107.5944[M-H-hexanoyl-CO2-Ac]⁻ 1089.5438[M-H-hexanoyl-CO2-Ac-H2O]⁻ 945.5759[M-H-hexanoyl-CO2-Ac-glc]⁻ 783.5012[M-H-hexanoyl-CO2-Ac-2glc]⁻ 621.4295 [M-H-hexanoyl-CO2-Ac-3glc]⁻	PPD1-glc-glc-glc-glc-hexanoyl-malonyl	hexanoyl-malonyl-Rb1	C63H104O27	1292.6765	B1, B2, B3, B4	4.65
26	6.82	973.5329	973.5346[M-H]⁻ 931.5246[M-H-Ac]⁻ 799.4980[M-H-Ac-xyl/ara]⁻ 637.4173[M-H-Ac-xyl/ara-glc]⁻ 475.3779[M-H-Ac-xyl/ara-2glc]⁻	PPT1-glc-glc-xyl/ara-acetyl	acetyl-NG-R1C	C49H82O19	974.545	B2	4.42
27	7.31	1261.645	1163.5847[M-H-hexanoyl]⁻ 1119.5947[M-H-hexanoyl-CO2]⁻ 1077.5891[M-H-hexanoyl-CO2-Ac]⁻ 945.5486[M-H-hexanoyl-CO2-Ac-xyl/ara]⁻ 783.4967[M-H-hexanoyl-CO2-Ac-xyl/ara-glc]⁻ 621.4977[M-H-hexanoyl-CO2-Ac-xyl/ara-2glc]⁻ 459.3786[M-H-hexanoyl-CO2-Ac-xyl/ara-3glc]⁻	PPD1-glc-glc-glc-xyl/ara-hexanoyl-malonyl	hexanoyl-malonyl-Rc	C62H102O26	1262.6659	B1, B2, B3, B4	0.26
28	7.36	1121.6043	1121.6058[M-H]⁻ 1079.5970[M-H-Ac]⁻ 1061.5862[M-H-Ac-H2O]⁻ 947.5568[M-H-Ac-xyl/ara]⁻ 917.5476[M-H-Ac-glc]⁻ 785.5089[M-H-Ac-glc-xyl/ara]⁻ 767.4896[M-H-Ac-glc-xyl/ara-H2O]⁻ 623.4883[M-H-Ac-2glc-xyl/ara]⁻ 461.3999[M-H-Ac-3glc-xyl/ara]⁻	PPD5-glc-glc-glc-xyl/ara-acetyl	H-acetyl-Rc	C55H94O23	1122.6186	B1, B2, B3	5.80
29	8.08	1193.5905	1193.5905[M-H]⁻ 1149.5988[M-H-CO2]⁻ 1107.5836[M-H-CO2-Ac]⁻ 945.5503[M-H-CO2-Ac-glc]⁻ 799.4961[M-H-CO2-Ac-glc-rha]⁻ 637.4177[M-H-CO2-Ac-2glc-rha]⁻ 475.3788[M-H-CO2-Ac-3glc-rha]⁻	PPT1-glc-glc-glc-rha-malonyl	malonyl-Re-Gb	C57H94O26	1194.6033	B1, B2, B4	4.19
30	8.16	1043.606	1043.6060[M-H]⁻ 945.5448[M-H-hexanoyl]⁻ 783.4916[M-H-hexanoyl-glc]⁻ 621.4397[M-H-hexanoyl-2glc]⁻ 459.3821[M-H-hexanoyl-3glc]⁻	PPD1-glc-glc-glc-hexanoyl	hexanoyl-Rd	C54H92O19	1044.6233	B1, B2, B3, B4	9.10

31	8.31	999.5543	999.5526[M-H]⁻ 931.5267[M-H-C4H4O]⁻ 799.4904[M-H-C4H4O-xyl/ara]⁻ 637.4442[M-H-C4H4O-xyl/ara-glc]⁻ 475.4149[M-H-C4H4O-xyl/ara-2glc]⁻	PPT1-glc-glc-xyl/ara-butenoyl	butenoyl-NG-R1	C51H84O19	1000.5607	B2	-1.40
32	8.43	1089.5827	1089.5809[M-H]⁻ 1047.5798[M-H-Ac]⁻ 1029.5652[M-H-Ac-H2O]⁻ 915.5452[M-H-Ac-xyl/ara]⁻ 753.4677[M-H-Ac-xyl/ara-glc]⁻ 621.4413[M-H-Ac-2xyl/ara-glc]⁻	PPD1-glc-glc-xyl/ara-xyl/ara-acetyl	acetyl-CS-III-G	C54H90O22	1090.5924	B1, B2, B3, B4	1.74
33	8.48	1043.606	1043.6060[M-H]⁻ 945.5465[M-H-hexanoyl]⁻ 783.4928[M-H-hexanoyl-glc]⁻ 621.4458[M-H-hexanoyl-2glc]⁻ 459.3973[M-H-hexanoyl-3glc]⁻	PPD1-glc-glc-glc-hexanoyl	hexanoyl-Rd	C54H92O19	1044.6233	B1, B2, B3, B4	9.10
34	8.74	971.5768	971.5768[M-H]⁻ 929.5511[M-H-Ac]⁻ 767.5098[M-H-Ac-glc]⁻ 605.4654[M-H-Ac-2glc]⁻ 455.5640[M-H-Ac-2glc-decadienoyl]⁻	OA-decadienoyl-2glc-acetyl	decadienoyl-acetyl-CS-2G	C54H84O15	972.581	B1, B2, B3, B4	-3.71
35	9.14	897.562	897.5620[M-H]⁻ 799.4937[M-H-hexanoyl]⁻ 637.4349[M-H-hexanoyl-glc]⁻ 475.3794[M-H-hexanoyl-2glc]⁻	PPT1-glc-glc-hexanoyl	hexanoyl-Rg1	C48H82O15	898.5654	B1, B2, B3, B4	-4.90
36	9.38	823.4842	823.4873[M-H]⁻ 781.4798[M-H-Ac]⁻ 763.4757[M-H-Ac-H2O]⁻ 619.4243[M-H-Ac-glc]⁻ 457.4639[M-H-Ac-2glc]⁻	PPT2-glc-glc-acetyl	acetyl-Rg9A	C44H72O14	824.4922	B1, B2, B3	0.24
37	9.68	1203.6863	1077.600[M-H-octanoyl]⁻ 915.5355[M-H-octanoyl-glc]⁻ 783.5193[M-H-octanoyl-glc-xyl/ara]⁻ 621.4446[M-H-octanoyl-2glc-xyl/ara]⁻ 459.3890[M-H-octanoyl-3glc-xyl/ara]⁻	PPD1-glc-glc-glc-xyl/ara-octanoyl	octanoyl-Rb2	C61H104O23	1204.6968	B1, B3	2.24
38	9.75	1023.6231	1023.6231[M-H]⁻ 979.5358[M-H-CO2]⁻ 937.5303[M-H-CO2-Ac]⁻ 783.4888[M-H-CO2-Ac-decanoyl]⁻ 621.4422[M-H-CO2-Ac-decanoyl-glc]⁻ 459.3918[M-H-CO2-Ac-decanoyl-2glc]⁻	PPD1-glc-glc-decanoyl-malonyl	decanoyl-malonyl-F2	C55H92O17	1024.6335	B1, B3, B4	2.54
39	9.81	867.5455	867.5455[M-H]⁻ 769.4904[M-H-hexanoyl]⁻ 637.4332[M-H-hexanoyl-xyl/ara]⁻ 475.3870[M-H-hexanoyl-xyl/ara-glc]⁻	PPT1-glc-xyl/ara-hexanoyl	hexanoyl-F3	C47H80O14	868.5548	B1, B2, B3, B4	1.73

40	10.08	943.5259	943.5289[M-H]⁻ 781.4644[M-H-glc]⁻ 619.4159[M-H-glc-glc]⁻ 457.3717[M-H-glc-glc-glc]⁻	PPT2-glc-glc-glc	Rg9-G	C48H80O18	944.5345	B1, B2, B3, B4	0.85
41	10.09	1073.5519	1073.5520[M-H]⁻ 1029.5669[M-H-CO2]⁻ 987.5538[M-H-CO2-Ac]⁻ 945.5395[M-H-CO2-2Ac]⁻ 783.4895[M-H-CO2-2Ac-glc]⁻ 621.4199[M-H-CO2-2Ac-2glc]⁻ 459.3966[M-H-CO2-2Ac-3glc]⁻	PPD1-glc-glc-glc-malonyl-acetyl	malonyl-acetyl-Rd	C53H86O22	1074.5611	B1, B2, B3, B4	1.30
42	10.56	833.5049	833.4575[M+FA-H]⁻ 787.5343[M-H]⁻ 637.4324[M-H-decadienoyl]⁻ 475.3793[M-H-decadienoyl-glc]⁻	PPT1-glc-decadienoyl	decadienoyl-Rh1	C46H76O10	788.5438	B2, B4	2.16
43	10.58	851.5228	851.5228[M-H]⁻ 783.4890[M-H-C4H4O]⁻ 637.4310[M-H-C4H4O-rha]⁻ 475.3724[M-H-C4H4O-rha-glc]⁻	PPT1-rha-glc-butenoyl	butenoyl-Rg2	C46H76O14	852.5235	B1, B2	-8.34
44	10.83	867.4659	867.4860[M-H]⁻ 823.4852[M-H-CO2]⁻ 781.5006[M-H-CO2-Ac]⁻ 619.3931[M-H-CO2-Ac-glc]⁻ 457.3654[M-H-CO2-Ac-2glc]⁻	PPT2-glc-glc-malonyl	malonyl-Rg9A	C45H72O16	868.482	B1, B2, B3, B4	9.57
45	10.89	971.5536	971.5555[M-H]⁻ 929.5439[M-H-Ac]⁻ 911.5370[M-H-Ac-H2O]⁻ 767.4978[M-H-Ac-glc]⁻ 621.4377[M-H-Ac-glc-rha]⁻ 459.3938[M-H-Ac-2glc-rha]⁻	PPD1-glc-glc-rha-acetyl	acetyl-F2-G	C50H84O18	972.5658	B1, B2, B3, B4	4.53
46	10.99	823.4839	823.4805 781.4802 763.4445 619.4298 457.3831	PPT2-glc-glc-acetyl	acetyl-Rg9B	C44H72O14	824.4922	B1, B2, B3, B4	0.61
47	11.24	1071.637	1071.6370[M-H]⁻ 945.5269[M-H-octanoyl]⁻ 783.5023[M-H-octanoyl-glc]⁻ 621.4619[M-H-octanoyl-2glc]⁻ 459.3769[M-H-octanoyl-3glc]⁻	PPD1-glc-glc-glc-octanoyl	octanoyl-Rd	C56H96O19	1072.6546	B1, B3, B4	9.14
48	11.5	857.4916	857.5184[M+FA-H]⁻ 811.4687[M-H]⁻ 769.4793[M-H-Ac]⁻ 637.4492[M-H-Ac-xyl/ara]⁻ 475.3522[M-H-Ac-xyl/ara-glc]⁻	PPT1-glc-xyl/ara-acetyl	acetyl-F3	C43H72O14	812.4922	B1	-1.98
49	11.65	941.5449	941.5559[M+FA-H]⁻ 895.5467[M-H]⁻ 799.4779[M-H-hexenoyl]⁻ 637.4152[M-H-hexenoyl-glc]⁻	PPT1-glc-glc-hexenoyl	hexenoyl-Rg1	C48H80O15	896.5497	B1, B2, B3	-5.36

50	11.84	895.5051	895.5379[M-H]⁻ 853.5021[M-H-Ac]⁻ 691.4512[M-H-Ac-glc]⁻ 529.3804[M-H-Ac-2glc]⁻ 459.3879[M-H-Ac-2glc-butanoyl]⁻	PPD1-butanoyl-glc-glc-acetyl	butanoyl-acetyl-F2	C49H80O15	896.5497	B1, B2, B3, B4	4.47
51	11.92	937.5157	937.5157[M-H]⁻ 893.4948[M-H-CO2]⁻ 825.4992[M-H-CO2-C4H4O]⁻ 783.5174[M-H-CO2-C4H4O-Ac]⁻ 621.4410[M-H-CO2-C4H4O-Ac-glc]⁻ 459.3828[M-H-CO2-C4H4O-Ac-2glc]⁻	PPD1-glc-glc-butenoyl-malonyl	butenoyl-malonyl-F2	C49H78O17	938.5239	B1, B2	0.43
52	12.58	857.4912	857.4756[M+FA-H]⁻ 811.4862[M-H]⁻ 769.4762[M-H-Ac]⁻ 637.4266[M-H-Ac-xyl/ara]⁻ 475.3754[M-H-Ac-xyl/ara-glc]⁻	PPT1-glc-xyl/ara-acetyl	acetyl-NG-R2	C43H72O14	812.4922	B2	-2.22
53	12.65	955.5617	955.5679[M+FA-H]⁻ 909.5567[M-H]⁻ 799.4911[M-H-heptenoyl]⁻ 637.4343[M-H-heptenoyl-glc]⁻ 475.3630[M-H-heptenoyl-2glc]⁻	PPT1-glc-glc-heptenoyl	heptenoyl-Rg1	C49H82O15	910.5654	B1, B2, B3, B4	0.99
54	12.71	989.5319	989.5584[M+FA-H]⁻ 943.5322[M-H]⁻ 781.4672[M-H-glc]⁻ 619.4539[M-H-2glc]⁻ 473.1998[M-H-2glc-rha]⁻	PPT3-glc-glc-rha	Rh5-GR	C48H80O18	944.5345	B1, B2, B3, B4	-5.83
55	12.91	1167.605	1167.6087[M+FA-H]⁻ 1121.5705[M-H]⁻ 945.5601[M-H-glcA]⁻ 783.4773[M-H-glcA-glc]⁻	PPD1-glc-glc-glc-glcA	Rd-GA	C54H90O24	1122.5822	B1, B2, B3, B4	3.48
56	13.08	1273.7025	1175.6161[M-H-hexanoyl]⁻ 1107.5975[M-H-hexanoyl-butenoyl]⁻ 945.5644[M-H-hexanoyl-butenoyl-glc]⁻ 783.4881[M-H-hexanoyl-butenoyl-2glc]⁻ 459.4059[M-H-hexanoyl-butenoyl-4glc]⁻	PPD1-glc-glc-glc-glc-butenoyl-hexanoyl	butenoyl-hexanoyl-Rb1	C64H106O25	1274.7023	B1, B2, B3	-6.28
57	13.08	799.4836	799.4738[M+FA-H]⁻ 753.4802[M-H]⁻ 621.4326[M-H-xyl/ara]⁻ 475.3831[M-H-xyl/ara-rha]⁻	PPT1-xyl/ara-rha	PPTXR1	C41H70O12	754.4867	B1, B2, B3	-1.73
58	13.36	985.5362	985.5364[M-H]⁻ 943.5270[M-H-Ac]⁻ 781.4993[M-H-Ac-glc]⁻ 619.4551[M-H-Ac-glc-glc]⁻ 457.3722[M-H-Ac-glc-glc-glc]⁻	PPT2-glc-glc-glc-acetyl	acetyl-Rg9-G	C50H82O19	986.545	B1, B2, B3, B4	1.01

59	13.61	981.5753	981.5460[M-H]⁻ 937.5208[M-H-CO2]⁻ 799.4679[M-H-CO2-nonenoyl]⁻ 637.4203[M-H-CO2-nonenoyl-glc]⁻ 475.3530[M-H-CO2-nonenoyl-2glc]⁻	PPT1-glc-glc-decenedioyl	decenedioyl-Rg1	C52H86O17	982.5865	B1, B2, B3	3.46
60	13.61	937.5889	937.5889[M-H]⁻ 799.4739[M-H-nonenoyl]⁻ 637.4274[M-H-nonenoyl-glc]⁻	PPT1-glc-glc-nonenoyl	nonenoyl-Rg1	C51H86O15	938.5967	B1, B2, B3, B4	0.00
61	13.64	969.5818	969.5809[M+FA-H]⁻ 923.5784[M-H]⁻ 799.4886[M-H-octenoyl]⁻ 637.4297[M-H-octenoyl-glc]⁻ 475.3873[M-H-octenoyl-2glc]⁻	PPT1-glc-glc-octenoyl	octenoyl-Rg1	C50H84O15	924.581	B1, B2, B3, B4	-5.63
62	13.97	955.5625	955.5697[M+FA-H]⁻ 909.5643[M-H]⁻ 799.4916[M-H-heptenoyl]⁻ 637.4201[M-H-heptenoyl-glc]⁻ 475.3739[M-H-heptenoyl-2glc]⁻	PPT1-glc-glc-heptenoyl	heptenoyl-Rg1	C49H82O15	910.5654	B1, B2, B4	-7.37
63	14.23	1085.629	1085.6290[M-H]⁻ 987.5536[M-H-hexanoyl]⁻ 945.5446[M-H-hexanoyl-Ac]⁻ 783.4824[M-H-hexanoyl-Ac-glc]⁻ 621.4212[M-H-hexanoyl-Ac-2glc]⁻ 459.4032[M-H-hexanoyl-Ac-3glc]⁻	PPD1-glc-glc-glc-acetyl-hexanoyl	acetyl-hexanoyl-Rd	C56H94O20	1086.6338	B1	-2.76
64	14.31	1101.6188	1101.6282[M+FA-H]⁻ 1055.6179[M-H]⁻ 931.5239[M-H-octenoyl]⁻ 799.4841[M-H-octenoyl-xyl/ara]⁻ 637.4358[M-H-octenoyl-xyl/ara-glc]⁻ 475.3804[M-H-octenoyl-xyl/ara-2glc]⁻	PPT1-glc-glc-xyl/ara-octenoyl	octenoyl-Ng-R1	C55H92O19	1056.6233	B1, B2	-2.27
65	14.65	1115.6338	1115.6404[M+FA-H]⁻ 1069.6316[M-H]⁻ 945.5469[M-H-octenoyl]⁻ 927.5274[M-H-octenoyl-H2O]⁻ 783.512[M-H-octenoyl-glc]⁻ 621.4138[M-H-octenoyl-2glc]⁻ 459.3984[M-H-octenoyl-3glc]⁻	PPD1-glc-glc-glc-octenoyl	octenoyl-Rd	C56H94O19	1070.6389	B1, B2	-0.47
66	14.78	799.4822	799.4674[M+FA-H]⁻ 753.4786[M-H]⁻ 621.4506[M-H-xyl/ara]⁻ 475.4142[M-H-xyl/ara-rha]⁻	PPT1-xyl/ara-rha	PPTXR2	C41H70O12	754.4867	B1, B3, B4	0.40

67	15	969.5794	969.5827[M+FA-H]⁻ 923.5824[M-H]⁻ 799.5001[M-H-octenoyl]⁻ 637.4387[M-H-octenoyl-glc]⁻ 475.3746[M-H-octenoyl-2glc]⁻	PPT1-glc-glc-octenoyl	octenoyl-Rg1	C50H84O15	924.581	B1, B2, B3, B4	-9.96
68	15.51	1061.5785	1061.6069[M+FA-H]⁻ 1015.5785[M-H]⁻ 947.5484[M-H-C4H4O]⁻ 929.5403[M-H-C4H4O-H2O]⁻ 785.5197[M-H-C4H4O-glc]⁻ 623.4469[M-H-C4H4O-2glc]⁻ 461.2736[M-H-C4H4O-3glc]⁻	PPD5-glc-glc-glc-butenoyl	H-butenoyl-Rd	C52H88O19	1016.592	B1, B2, B3, B4	5.61
69	15.78	909.5574	909.5553[M-H]⁻ 799.4838[M-H-heptenoyl]⁻ 637.4369[M-H-heptenoyl-glc]⁻ 475.3916[M-H-heptenoyl-2glc]⁻	PPT1-2glc-heptenoyl	heptenoyl-Rg1	C49H82O15	910.5654	B1, B2, B4	0.22
70	16.28	1029.564	1029.5637[M+FA-H]⁻ 983.5609[M-H]⁻ 915.5307[M-H-butenoyl]⁻ 783.5307[M-H-butenoyl-xyl/ara]⁻ 765.4937[M-H-butenoyl-xyl/ara-H2O]⁻ 621.4684[M-H-butenoyl-xyl/ara-glc]⁻ 459.3944[M-H-butenoyl-xyl/ara-2glc]⁻	PPD1-glc-glc-xyl/ara-butenoyl	butenoyl-CS-III	C51H84O18	984.5658	B1, B2, B3, B4	-2.95
71	16.42	1201.678	1077.5909[M-H-octenoyl-H2O]⁻ 1059.5682[M-H-octenoyl-H2O]⁻ 945.5380[M-H-octenoyl-xyl/ara]⁻ 915.5076[M-H-octenoyl-glc]⁻ 783.4889[M-H-octenoyl-xyl/ara-glc]⁻ 459.3851459.3716 [M-H-octenoyl-xyl/ara-3glc]⁻	PPD1-glc-glc-glc-xyl/ara-octenoyl	octenoyl-Rb2	C61H102O23	1202.6812	B1, B2, B3, B4	-3.83
72	16.54	969.5763	969.5650[M+FA-H]⁻ 923.5695[M-H]⁻ 799.4805[M-H-octenoyl]⁻ 637.4648[M-H-octenoyl-glc]⁻ 475.3777[M-H-octenoyl-glc-glc]⁻	PPT1-glc-glc-octenoyl	octenoyl-Rg1	C50H84O15	924.581	B1, B2	4.01
73	16.8	925.5497	925.5522[M+FA-H]⁻ 879.5553[M-H]⁻ 769.4679[M-H-heptenoyl]⁻ 637.4494[M-H-heptenoyl-xyl/ara]⁻ 475.3921[M-H-heptenoyl-xyl/ara-glc]⁻	PPT1-glc-xyl/ara-heptenoyl	heptenoyl-NG-R2	C48H80O14	880.5548	B1	-9.44
74	16.95	1201.6724	1077.5879[M-H-octenoyl]⁻ 1059.5791[M-H-octenoyl-H2O]⁻ 945.5266[M-H-octenoyl-xyl/ara]⁻ 783.4699[M-H-octenoyl-	PPD1-glc-glc-glc-xyl/ara-octenoyl	octenoyl-Rb2	C61H102O23	1202.6812	B1, B2, B3, B4	0.83

			xyl/ara-glc]$^-$ 765.4786[M-H-octenoyl-xyl/ara-H2O]$^-$ 459.3716 [M-H-octenoyl-xyl/ara-3glc]$^-$						
75	17.08	969.5774	969.5812[M+FA-H]$^-$ 923.5743[M-H]$^-$ 799.4866[M-H-octenoyl]$^-$ 637.4502[M-H-octenoyl-glc]$^-$ 475.3798[M-H-octenoyl-2glc]$^-$	PPT1-glc-glc-octenoyl	octenoyl-Rg1	C50H84O15	924.581	B1, B2, B3, B4	-1.19
76	17.25	923.5729	923.5792[M-H]$^-$ 799.4982[M-H-octenoyl]$^-$ 637.4313[M-H-octenoyl-glc]$^-$ 475.3802[M-H-octenoyl-2glc]$^-$	PPT1-glc-glc-octenoyl	octenoyl-Rg1	C50H84O15	924.581	B1, B2, B3, B4	0.32
77	17.3	1101.646	1101.5773[M+FA-H]$^-$ 1055.6137[M-H]$^-$ 945.5383[M-H-heptenoyl]$^-$ 783.5014[M-H-heptenoyl-glc]$^-$ 621.4286[M-H-heptenoyl-2glc]$^-$ 459.3725[M-H-heptenoyl-3glc]$^-$	PPD1-glc-glc-glc-heptenoyl	heptenoyl-Rd	C55H92O19	1056.6233	B1, B2, B3, B4	1.71
78	18.19	939.5674	939.5777[M+FA-H]$^-$ 893.5607[M-H]$^-$ 769.4776[M-H-octenoyl]$^-$ 637.4229[M-H-octenoyl-xyl/ara]$^-$ 475.3782[M-H-octenoyl-xyl/ara-glc]$^-$	PPT1-glc-xyl/ara-octenoyl	octenoyl-Rg1	C49H84O12	894.5705	B1, B2	2.24
79	18.89	1115.6364	1115.6374[M+FA-H]$^-$ 1069.6358[M-H]$^-$ 945.5439[M-H-octenoyl]$^-$ 927.5297[M-H-octenoyl-H2O]$^-$ 783.4941[M-H-octenoyl-glc]$^-$ 765.4755[M-H-octenoyl-glc-H2O]$^-$ 621.4241[M-H-octenoyl-2glc]$^-$ 459.3803[M-H-octenoyl-3glc]$^-$	PPD1-glc-glc-glc-octenoyl	octenoyl-Rd	C56H94O19	1070.6389	B1, B2, B3, B4	-4.39
80	20.89	1105.6061	1105.6053[M+Cl-H]$^-$ 1069.6340[M-H]$^-$ 945.5399[]M-H-octenoyl]$^-$ 783.4971[M-H-octenoyl-glc]$^-$ 621.4394[M-H-octenoyl-2glc]$^-$ 459.4036[M-H-octenoyl-3glc]$^-$	PPD1-glc-glc-glc-octenoyl	octenoyl-Rd	C56H94O19	1070.6389	B1, B2, B3, B4	-2.71
81	21.46	1115.6369	1115.6300[M+FA-H]$^-$ 1069.6277[M-H]$^-$ 945.5368[M-H-octenoyl]$^-$ 783.5136[M-H-octenoyl-glc]$^-$ 621.4386[M-H-octenoyl-2glc]$^-$ 459.3829[M-H-octenoyl-3glc]$^-$	PPD1-glc-glc-glc-octenoyl	octenoyl-Rd	C56H94O19	1070.6389	B1, B2	3.18

Table S2

MS and MS/MS fragmentation of the known ginsenosides.

No.	Rt	Found At Mass	MS E Fragmentation	Identification	Name	Formula	Theoretical Mass (Da)	Source	Ppm
1	1.18	861.4858	861.4879[M+FA-H]$^{-}$ 815.4800[M-H]$^{-}$ 653.4251[M-H-glc]$^{-}$ 635.4230[M-H-glc-H2O]$^{-}$ 491.3715[M-H-2glc]$^{-}$	PG-glc-glc	Panajaponol A/isomer	C42H72O15	816.4871	B1, B2, B3, B4	-0.86
2	1.25	863.5005	863.5007[M+FA-H]$^{-}$ 817.4953[M-H]$^{-}$ 655.4458[M-H-glc]$^{-}$ 493.3921[M-H-2glc]$^{-}$	PPT4-glc-glc	20-S-Rf3/ isomer	C42H74O15	818.5028	B1, B3, B4	-0.37
3	1.37	1003.5493	1003.5530[M-H]$^{-}$ 961.5460[M-H-Ac]$^{-}$ 943.5340[M-H-Ac-H2O]$^{-}$ 799.4966[M-H-Ac-glc]$^{-}$ 637.4406[M-H-Ac-glc-glc]$^{-}$ 475.3464[M-H-Ac-glc-glc-glc]$^{-}$	PPT1-glc-glc-glc-acetyl	6-acetyl ginsenoside-Rg3/isomer	C50H84O20	1004.5556	B1, B2, B3, B4	-1.49
4	1.52	861.4849	861.4920[M+FA-H]$^{-}$ 815.4839[M-H]$^{-}$ 653.4332[M-H]$^{-}$ 491.3645[M-H-2glc]$^{-}$	PG-glc-glc	Panajaponol A/isomer	C42H72O15	816.4871	B2, B3, B4	-5.64
5	1.59	829.4844	829.4816[M+FA-H]$^{-}$ 783.4877[M-H]$^{-}$ 621.4538[M-H-glc]$^{-}$ 475.3971[M-H-glc-rha]$^{-}$	PPT1-rha-glc	Rg2 isomer	C42H72O13	784.4973	B1, B3	2.30
6	1.6	885.4866	885.4866[M-H]$^{-}$ 841.5011[M-H-CO2]$^{-}$ 799.4890[M-H-CO2-Ac]$^{-}$ 637.4360[M-H-CO2-Ac-glc]$^{-}$ 475.3743[M-H-CO2-Ac-glc-glc]$^{-}$	glc-PPT1-glc-malonyl	mRg1 isomer	C45H74O17	886.4926	B1, B2, B3	-2.03
7	1.62	841.4954	841.4990[M-H]$^{-}$ 799.4915[M-H-Ac]$^{-}$ 781.4738[M-H-Ac-H2O]$^{-}$ 679.4414[M-H-glc]$^{-}$ 637.4319[M-H-Ac-glc]$^{-}$ 619.4212[M-H-Ac-glc-H2O]$^{-}$ 475.3776[M-H-Ac-glc-glc]	acetyl-PPT1-glc-glc	noto-Rt/isomer	C44H74O15	842.5028	B1, B2, B3, B4	-0.48
8	1.62	827.4795	827.4811[M+FA-H]$^{-}$ 781.4744[M-H]$^{-}$ 619.4250[M-H-glc]$^{-}$ 475.3826[M-H-glc-glc+H2O]$^{-}$	PPT2-glc-glc	G-Rh15/isomer	C42H70O13	782.4816	B1, B2, B3, B4	-0.77

9	1.63	987.5533	987.5576[M-H]⁻ 945.5478[M-H-Ac]⁻ 927.5359[M-H-Ac-H2O]⁻ 799.4704[M-H-Ac-rha]⁻ 637.4245[M-H-Ac-rha-glc]⁻ 475.3881[M-H-Ac-rha-glc-glc]⁻	PPT1-glc-glc-rha-acetyl	Pseudoginsenoside Rs1/isomer	C50H84O19	988.5607	B1, B2, B3, B4	-0.41
10	1.66	1003.5456	1003.5499[M-H]⁻ 961.5429[M-H-Ac]⁻ 943.5308[M-H-Ac-H2O]⁻ 799.4796[M-H-Ac-glc]⁻ 637.4367[M-H-Ac-2glc]⁻ 475.3889[M-H-Ac-3glc]⁻	PPT1-glc-glc-glc-acetyl	6-acetyl ginsenoside-Rg3/isomer	C50H84O20	1004.5556	B2, B4	2.19
11	1.79	1031.544	1031.5449[M-H]⁻ 987.5586[M-H-CO2]⁻ 945.5471[M-H-CO2-Ac]⁻ 927.5362[M-H-CO2-Ac-H2O]⁻ 799.4916[M-H-CO2-Ac-rha]⁻ 637.4381[M-H-CO2-Ac-rha-glc]⁻ 475.3786[M-H-CO2-Ac-rha-1glc]⁻	PPT1-glc-glc-rha-malonyl	mRd isomer	C51H84O21	1032.5505	B1, B3, B4	-1.26
12	1.87	861.4859	861.4826[M+FA-H]⁻ 815.4800[M-H]⁻ 653.4314[M-H-glc]⁻ 491.3729[M-H-2glc]	PG-glc-glc	Panajaponol A/isomer	C42H72O15	816.4871	B1, B4	-0.86
13	1.89	885.4879	885.4878[M-H]⁻ 841.4982[M-H-CO2]⁻ 799.4874[M-H-CO2-Ac]⁻ 781.4768[M-H-CO2-Ac-H2O]⁻ 679.4427[M-H-CO2-glc]⁻ 637.4336[M-H-CO2-Ac-glc]⁻ 619.4224[M-H-CO2-Ac-glc-H2O]⁻ 475.3784[M-H-CO2-Ac-2glc]⁻	glc-PPT1-glc-malonyl	mRg1 isomer	C45H74O17	886.4926	B1, B2, B3, B4	-3.50
14	1.93	1123.5858	1123.5959[M-H]⁻ 961.5497[M-H-glc]⁻ 799.4793[M-H-2glc]⁻ 637.4076[M-H-3glc]⁻ 475.3864[M-H-4glc]⁻	PPT1-4glc	Notoginsenoside Fh7 isomer	C54H92O24	1124.5979	B1, B3, B4	3.83
15	1.95	799.4848	799.4875[M-H]⁻ 637.4325[M-H-glc]⁻ 475.3799[M-H-2glc]⁻	PPT1-2glc	Rg1 isomer	C42H72O14	800.4922	B1, B2, B3, B4	-0.50

16	1.96	945.5399	945.5496[M-H]ˉ 799.4873[M-H-rha]ˉ 637.4582[M-H-glc-rha]ˉ 475.3807[M-H-rha-glc-glc]ˉ	PPT1-glc-glc-rha	G-Re isomer	$C_{48}H_{82}O_{18}$	946.5501	B2	2.54
17	1.98	829.4873	829.4834[M+FA-H]ˉ 783.4901[M-H]ˉ 621.4325[M-H-glc]ˉ 475.3925[M-H-glc-rha]ˉ	PPT1-rha-glc	Rg2 isomer	$C_{42}H_{72}O_{13}$	784.4973	B1, B4	-0.77
18	2.09	987.5524	987.5571[M-H]ˉ 945.5453[M-H-Ac]ˉ 927.5352[M-H-Ac-H2O]ˉ 799.4873[M-H-Ac-rha]ˉ 637.4321[M-H-Ac-glc-rha]ˉ 475.3796[M-H-Ac-glc-rha-glc]ˉ	glc-PPT1-glc-rha-acetyl	Pseudoginsenoside Rs1/isomer	$C_{50}H_{84}O_{19}$	988.5607	B1, B2, B3, B4	0.51
19	2.1	1031.5443	1031.5453[M-H]ˉ 987.5562[M-H-CO2]ˉ 945.5456[M-H-CO2-Ac]ˉ 927.5361[M-H-CO2-Ac-H2O]ˉ 799.4901[M-H-CO2-Ac-rha]ˉ 637.4329[M-H-CO2-Ac-rha-glc]ˉ 475.3768[M-H-CO2-Ac-rha-2glc]ˉ	PPT1-glc-glc-rha-malonyl	mRd isomer	$C_{51}H_{84}O_{21}$	1032.5505	B1, B2, B3, B4	-1.55
20	2.15	861.4839	861.4863[M+FA-H]ˉ 815.4862[M-H]ˉ 653.4573[M-H-glc]ˉ 491.3827[M-H-2glc]ˉ	PG-glc-glc	Panajaponol A/isomer	$C_{42}H_{72}O_{15}$	816.4871	B1, B3, B4	-8.46
21	2.21	885.4873	885.4875[M-H]ˉ 841.4975[M-H-CO2]ˉ 799.4869[M-H-CO2-Ac]ˉ 637.4317[M-H-CO2-Ac-glc]ˉ 475.3839[M-H-CO2-Ac-2glc]	glc-PPT1-glc-malonyl	mRg1 isomer	$C_{45}H_{74}O_{17}$	886.4926	B1, B2, B3, B4	-2.82
22	2.21	841.498	841.4979[M-H]ˉ 799.4859[M-H-Ac]ˉ 637.4293[M-H-Ac-glc]ˉ 475.3849[M-H-Ac-2glc]	acetyl-PPT1-glc-glc	noto-Rt/isomer	$C_{44}H_{74}O_{15}$	842.5028	B1, B2, B3, B4	-3.57
23	2.25	987.5548	987.5547[M-H]ˉ 945.5413[M-H-Ac]ˉ 927.5322[M-H-Ac-H2O]ˉ 799.4837[M-H-Ac-rha]ˉ 783.4981[M-H-Ac-glc]ˉ 781.4879[M-H-Ac-rha-H2O]ˉ 637.4303[M-H-Ac-glc-rha]ˉ 475.3716[M-H-Ac-glc-rha-glc]ˉ	glc-PPT1-glc-rha-acetyl	Pseudoginsenoside Rs1/isomer	$C_{50}H_{84}O_{19}$	988.5607	B1, B2, B3, B4	-1.92

24	2.34	827.4815	827.4824[M+FA-H]⁻ 781.4755[M-H]⁻ 619.4175[M-H-glc]⁻ 457.3386[M-H-2glc]⁻	PPT2-glc-glc	G-Rh15/isomer	$C_{42}H_{70}O_{13}$	782.4816	B1, B2, B3, B4	-2.18
25	2.36	799.4806	799.4628[M-H]⁻ 637.4353[M-H-glc]⁻ 475.3788[M-H-2glc]⁻	PPT1-2glc	Rg1 isomer	$C_{42}H_{72}O_{14}$	800.4922	B1, B2, B3, B4	4.75
26	2.38	945.5413	945.5446[M-H]⁻ 783.4864[M-H-glc]⁻ 637.4350[M-H-glc-rha]⁻ 475.3736[M-H-rha-glc-glc]⁻	PPT1-glc-glc-rha	G-Re isomer	$C_{48}H_{82}O_{18}$	946.5501	B1, B2, B3, B4	1.06
27	2.71	861.4856	861.4888[M+FA-H]⁻ 815.4839[M-H]⁻ 653.4426[M-H-glc]⁻ 491.3645[M-H-2glc]⁻	PG-glc-glc	Panajaponol A/isomer	$C_{42}H_{72}O_{15}$	816.4871	B1, B2, B3, B4	-5.64
28	2.72	799.4849	799.4803[M-H]⁻ 637.4140[M-H-glc]⁻ 475.3608[M-H-glc-glc]⁻	PPT1-2glc	Rg1 isomer	$C_{42}H_{72}O_{14}$	800.4922	B1, B2, B3, B4	-0.63
29	2.74	885.4877	885.4880[M-H]⁻ 841.4993[M-H-CO2]⁻ 799.4819[M-H-CO2-Ac]⁻ 637.4325[M-H-CO2-Ac-glc]⁻ 619.4209[M-H-CO2-Ac-glc-H2O]⁻ 475.3920[M-H-CO2-Ac-2glc]⁻	glc-PPT1-glc-malonyl	mRg1 isomer	$C_{45}H_{74}O_{17}$	886.4926	B1, B2, B3, B4	-3.28
30	2.77	841.4953	841.5016[M-H]⁻ 799.4834[M-H-Ac]⁻ 781.4688[M-H-Ac-H2O]⁻ 637.4381[M-H-Ac-glc]⁻ 619.4221[M-H-Ac-glc-H2O]⁻ 475.3735[M-H-Ac-2glc]⁻	PPT1-glc-glc-acetyl	noto-Rt/isomer	$C_{44}H_{74}O_{15}$	842.5028	B1, B2, B3, B4	-0.36
31	2.79	945.5448	945.5448[M-H]⁻ 799.5061[M-H-rha]⁻ 637.4342[M-H-rha-glc]⁻ 475.3576[M-H-rha-2glc]⁻	PPT1-glc-glc-rha	G-Re isomer	$C_{48}H_{82}O_{18}$	946.5501	B1, B3	-2.64
32	2.82	863.4849	863.4849[M+FA-H]⁻ 817.4966[M-H]⁻ 655.4481[M-H-glc]⁻ 493.3895[M-H-2glc]⁻	PPT4-glc-glc	20-S-Rf3/ isomer	$C_{42}H_{74}O_{15}$	818.5028	B1, B2, B3, B4	-1.96
33	2.83	987.5541	987.5572[M-H]⁻ 945.5459[M-H-Ac] 927.5299[M-H-Ac-H2O]⁻ 799.4982[M-H-Ac-rha]⁻	PPT1-glc-glc-rha-acetyl	20-S-Rf3/ isomer	$C_{50}H_{84}O_{19}$	988.5607	B1, B2, B3, B4	-1.22

			781.4555[M-H-Ac-rha-H2O]⁻ 637.4408[M-H-Ac-rha-glc]⁻ 475.3925[M-H-Ac-rha-glc]⁻						
34	2.84	1093.5771	1093.5819[M-H]⁻ 931.5391[M-H-glc]⁻ 799.5220[M-H-glc-xyl/ara]⁻ 637.4491[M-H-xyl/ara-2glc]⁻ 475.3916[M-H-3glc-xyl/ara]⁻	PPT1-glc-glc-glc-xyl/ara	20S-sanchirhinosides A6 isomer	C53H90O23	1094.5873	B1, B2, B3, B4	2.19
35	2.87	1031.5437	1031.5450[M-H]⁻ 987.5545[M-H-CO2]⁻ 927.5376[M-H-CO2-Ac-H2O]⁻ 799.4965[M-H-CO2-Ac-rha]⁻ 637.4275[M-H-CO2-Ac-rha-glc]⁻ 475.3859[M-H-CO2-Ac-rha-2glc]⁻	PPT1-glc-glc-rha-malonyl	mRd isomer	C51H84O21	1032.5505	B1, B2, B3, B4	-0.97
36	2.92	1123.5888	1123.5888[M-H]⁻ 961.5481[M-H-glc]⁻ 799.4867[M-H-2glc]⁻ 637.4181[M-H-3glc]⁻ 475.3865[M-H-4glc]⁻	PPT1-glc-glc-glc-glc	Notoginsenoside Fh7 isomer	C54H92O24	1124.5979	B1, B4	1.16
37	3	861.4824	815.4809[M-H]⁻ 653.4293[M-H-glc]⁻ 635.4135[M-H-glc-H2O]⁻ 491.3729[M-H-2glc]⁻	PG-glc-glc	Panajaponol A/isomer	C42H72O15	816.4871	B1, B2, B3, B4	-1.96
38	3.07	885.4847	885.4068 841.5024 799.4841 781.4765 637.4359 475.3661	glc-PPT1-glc-malonyl	mRg1 isomer	C45H74O17	886.4926	B2, B3, B4	0.11
39	3.16	811.484	811.4747[M-H]⁻ 769.4617[M-H-Ac]⁻ 637.4069[M-H-Ac-xyl/ara]⁻ 475.3763[M-H-Ac-xyl/ara-glc]⁻	PPT1-glc-ara/xyl-acetyl	β-D-Glucopyranoside, (3β,6α,12β,20*R*)-3,12,20-trihydroxydammar-24-en-6-yl	C43H72O14	812.4922	B3	0.49

					2-*O*-β-D-xylopyranosyl-, 6-acetate isomer				
40	3.2	961.5397	961.5397[M-H]⁻ 799.4887[M-H-glc]⁻ 637.4224[M-H-2glc]⁻ 475.3553[M-H-3glc]⁻	PPT1-glc-glc-glc	Re1 isomer	$C_{48}H_{82}O_{19}$	962.545	B1, B2, B3, B4	-2.60
41	3.3	947.52	947.5208[M-H]⁻ 815.4781[M-H-xyl/ara]⁻ 653.4309[M-H-xyl/ara-glc]⁻ 491.3935[M-H-xyl/ara-2glc]⁻	PG-glc-glc-xyl/ara	Vinaginsenoside R6 isomer	$C_{47}H_{80}O_{19}$	948.5294	B1, B2, B3	1.69
42	3.37523	815.4816	815.4802[M-H]⁻ 653.4259[M-H-glc]⁻ 491.3736[M-H-2glc] 391.2856	PG(母核)-2glc 或如图	Panajaponol A/isomer	$C_{42}H_{72}O_{15}$	816.4871	B1, B2, B3, B4	-2.82
43	3.4	863.4889	863.4925[M+FA-H]⁻ 817.4953[M-H]⁻ 655.4112[M-H-glc]⁻ 493.3860[M-H-2glc]⁻	PPT4-glc-glc	20-S-Rf3/ isomer	$C_{42}H_{74}O_{15}$	818.5028	B1, B2, B3, B4	-0.37
44	3.67	1123.5924	1123.5951[M-H]⁻ 961.5277[M-H-glc]⁻ 799.4742[M-H-2glc]⁻ 637.4282[M-H-3glc]⁻ 475.3876[M-H-4glc]⁻	PPT1-4glc	Notoginsenoside Fh7 isomer	$C_{54}H_{92}O_{24}$	1124.5979	B1, B2, B3, B4	-2.05
45	3.67	861.4837	861.4795 815.4737 653.4092 491.3714	PG-glc-glc	Panajaponol A/isomer	$C_{42}H_{72}O_{15}$	816.4871	B1, B2, B4	6.87
46	3.68	961.53826	961.5401[M-H]⁻ 799.4852[M-H-Glc]⁻	G-Re3	G-Re3	$C_{48}H_{82}O_{19}$	962.545	B1, B2, B3, B4	-1.10
47	3.69	811.4863	811.4819[M-H]⁻ 769.4915[M-H-Ac]⁻ 751.4680[M-H-Ac-H2O]⁻ 637.4696[M-H-Ac-xyl/ara]⁻ 475.3877[M-H-Ac-xyl/ara-glc]⁻	PPT1-glc-ara/xyl-acetyl	β-D-Glucopyranoside, (3β,6α,12β,	$C_{43}H_{72}O_{14}$	812.4922	B3	-2.34

					20*R*)-3,12,20-trihydroxydammar-24-en-6-yl 2-*O*-β-D-xylopyranosyl-, 6-acetate isomer				
48	3.79	1009.5535	1009.5593[M+FA-H]⁻ 963.5464[M-H]⁻ 801.4990[M-H-glc]⁻ 639.4332[M-H-2glc]⁻ 477.3752[M-H-3glc]⁻	PPD4/PPT5-glc-glc-glc	Re-mo isomer	C48H84O19	964.5607	B1, B2, B3	6.75
49	3.82	831.4767	831.4831[M+FA-H]⁻ 785.4672[M-H]⁻ 653.4260[M-H-xyl/ara]⁻ 491.3778[M-H-xyl/ara-glc]⁻	PG-glc-ara/xyl	M-R2/isomer	C41H70O14	786.4766	B1, B2, B3, B4	2.04
50	4.15	787.4832	787.4909[M-H]⁻ 655.4474[M-H-xyl/ara]⁻ 493.3920[M-H-xyl/ara-glc]⁻	PPT4-glc-xyl/ara	3β,12β,20,25-tetrahydroxydammarane-6-O-β-D-xylopyranosyl-(1→2)-β-D-glucopyranoside isomer	C41H72O14	788.4922	B1	1.52
51	4.45	653.4267	653.3360[M-H]⁻ 491.2973[M-H-glc]⁻	PG-glc	PG-F11 isomer	C36H62O10	654.4343	B1, B2, B3, B4	-0.31

52	4.48	961.538	961.5409[M-H]$^-$ 799.4851[M-H-glc]$^-$ 781.4779[M-H-glc-H2O]$^-$ 637.4334[M-H-2glc-] 475.3780[M-H-3glc]$^-$	PPT1-glc-glc-glc	Re1 isomer	C48H82O19	962.545	B1, B2, B3, B4	-0.83
53	4.56	811.4856	811.4881[M-H]$^-$ 769.4817[M-H-Ac]$^-$ 751.4561[M-H-Ac-H2O]$^-$ 637.4559[M-H-Ac-xyl/ara]$^-$ 619.4267[M-H-Ac-xyl/ara-H2O]$^-$ 475.3810[M-H-Ac-xyl/ara-glc]$^-$	PPT1-glc-ara/xyl-acetyl	β-D-Glucopyranoside, (3β,6α,12β,20*R*)-3,12,20-trihydroxydammar-24-en-6-yl 2-*O*-β-D-xylopyranosyl-, 6-acetate isomer	C43H72O14	812.4922	B2, B3, B4	-1.48
54	4.61	1007.5436	1007.5537[M+FA-H]$^-$ 961.5420[M-H]$^-$ 799.4781[M-H-Glc]$^-$	20-glu-G-Rf	20-glu-G-Rf	C48H82O19	962.545	B1, B2, B3, B4	-4.99
55	4.6352	931.52836	931.5332[M-H]$^-$ 799.4894[M-H-Ara(f)]$^-$ 637.4357[M-H-Ara(f)-Glc]$^-$ 475.3795[M-H-Ara(f)-2Glc]$^-$	G-Re4	G-Re4	C47H80O18	932.5345	B1, B2, B3, B4	-1.78
56	4.66	845.4894	845.5101[M+FA-H]$^-$ 799.4923[M-H]$^-$ 653.4353[M-H-rha]$^-$ 491.3664[M-H-rha-glc]$^-$	PG-glc-rha	PG-F11 isomer	C42H72O14	800.4922	B1, B2, B3, B4	-9.88
57	4.68	831.475	831.4847[M+FA-H]$^-$ 785.4700[M-H]$^-$ 653.4272[M-H-ara/xyl]$^-$ 635.4084[M-H-ara/xyl-H2O]$^-$ 491.3704[M-H-ara/xyl-glc]$^-$	PG-glc-ara/xyl	M-R2/isomer	C41H70O14	786.4766	B1, B2, B3, B4	-1.53

58	4.73194	1007.5446	1007.5449[M+FA-H]⁻ 961.5403[M-H]⁻ 799.4850[M-H-Glc]⁻ 637.4181[M-H-2Glc]⁻	G-Re2	G-Re2	$C_{48}H_{82}O_{19}$	962.545	B1, B2, B3, B4	-3.22
59	4.82	1077.5826	1077.5888[M-H]⁻ 945.5443[M-H-xyl/ara]⁻ 799.4837[M-H-xyl/ara-rha]⁻ 783.4936[M-H-xyl/ara-glc]⁻ 637.4249[M-H-rha-xyl/ara-glc]⁻ 475.3740[M-H-rha-xyl/ara-2glc]⁻	ara/xyl-rha-PPT1-glc-glc	Floralginsenoside M isomer	$C_{53}H_{90}O_{22}$	1078.5924	B1, B2, B3, B4	1.86
60	4.85	1093.5789	1093.5856[M-H]⁻ 931.5350[M-H-glc]⁻ 799.4631[M-H-glc-xyl/ara]⁻ 637.4243[M-H-xyl/ara-2glc]⁻ 475.3803[M-H-3glc-xyl/ara]⁻	PPT1-glc-glc-glc-xyl/ara	20S-sanchirhinosides A6 isomer	$C_{53}H_{90}O_{23}$	1094.5873	B1, B2, B3, B4	0.55
61	4.9	883.5063	883.5079[M-H]⁻ 841.4887[M-H-Ac]⁻ 799.4767[M-H-2Ac]⁻ 781.4664[M-H-2Ac-H2O]⁻ 763.4465[M-H-2Ac-2H2O]⁻ 637.4242[M-H-2Ac-glc]⁻ 475.3739[M-H-2Ac-2glc]⁻	PPT1-glc-glc-acetyl-acetyl	6',6"-di-O-acetyl Rg1 isomer	$C_{46}H_{76}O_{16}$	884.5133	B1, B2, B3, B4	-0.91
62	4.91	861.4853	861.4935[M+FA-H]⁻ 815.4784[M-H]⁻ 653.4239[M-H-glc]⁻ 635.4399[M-H-glc-H2O]⁻ 491.3707[M-H-2glc]⁻	PG-glc-glc	Panajaponol A/isomer	$C_{42}H_{72}O_{15}$	816.4871	B1, B2, B3, B4	1.10
63	4.91	811.4826	811.5180[M-H]⁻ 769.4801[M-H-Ac]⁻ 751.4666[M-H-Ac-H2O]⁻ 637.4343[M-H-Ac-xyl/ara]⁻ 619.4128[M-H-Ac-xyl/ara-H2O]⁻ 475.3879[M-H-Ac-xyl/ara-glc]⁻	PPT1-glc-ara/xyl-acetyl	β-D-Glucopyranoside, (3β,6α,12β,20R)-3,12,20-trihydroxydammar-24-en-6-yl 2-O-β-D-	$C_{43}H_{72}O_{14}$	812.4922	B2, B4	2.22

					xylopyranosyl-, 6-acetate isomer				
64	5.01	845.4846	845.4843[M+FA-H]⁻ 799.4819[M-H]⁻ 653.4498[M-H-rha]⁻ 491.3980[M-H-rha-glc]⁻	PG-glc-rha	PG-F11 isomer	C42H72O14	800.4922	B2, B3	3.01
65	5.03	701.4383	701.4401[M+FA-H]⁻ 655.4375[M-H]⁻ 493.3932[M-H-glc]⁻	PPT4-rha	α-L-Mannopyranoside, (3β,12β)-3,12,20,24,25-pentahydroxydammaran-6-yl 6-deoxy- isomer	C36H64O10	656.4499	B3	7.02
66	5.03945	653.42632	653.4280[M-H]⁻ 491.3788[M-glc]⁻	PG-glc	PG-RT5 isomer	C36H62O10	654.4343	B1, B2, B3, B4	0.28
67	5.08	931.5247	931.5292[M-H]⁻ 799.4904[M-H-Xyl]⁻ 637.4294[M-H-Xyl-Glc]⁻ 475.3803[M-H-Xyl-2Glc]⁻	PPT1-glc-glc-xyl/ara	NG-R1 isomer	C47H80O18	932.5345	B1, B2, B3, B4	2.15
68	5.19	1107.5973	1107.5999[M-H]⁻ 961.5520[M-H-rha]⁻ 945.5238[M-H-glc]⁻ 927.4901[M-H-glc-H2O]⁻ 783.5103[M-H-2glc]⁻ 637.4377[M-H-2glc-rha]⁻ 475.3802[M-H-3glc-rha]⁻	glc-glc-PPT1-glc-rha	Yesanchinoside E/isomer	C54H92O23	1108.6029	B2	-1.99
69	5.19284	931.52841	977.5361[M+HCOO]⁻ 931.5323[M-H]⁻ 799.4895[M-H-Xyl]⁻ 637.4340[M-H-Xyl-Glc]⁻	NG-R1	NG-R1	C47H80O18	932.5345	B1, B2, B3, B4	-1.84

70	5.22	1077.586	1077.5891[M-H]⁻ 945.5427[M-H-ara/xyl]⁻ 931.5314[M-H-rha]⁻ 799.5007[M-H-rha-ara/xyl]⁻ 637.4326[M-H-rha-xyl/ara-glc]⁻ 475.3805[M-H-rha-xyl/ara-2glc]⁻	ara/xyl-PPT1-glc-glc-rha	Floralginsenoside M isomer	C53H90O22	1078.5924	B1, B2, B3, B4	-1.30
71	5.3	799.4853	799.4887[M-H]⁻ 653.4289[M-H-rha]⁻ 635.4220[M-H-rha-H2O]⁻ 491.3738[M-H-rha-glc]⁻	PG-F11 isomer	PG-F11 isomer	C42H72O14	800.4922	B1, B2, B3, B4	-1.13
72	5.34	961.53971	961.5439[M-H]⁻ 799.4901[M-H-Glc]⁻ 637.4361[M-H-2glc] 475.3979[M-H-3glc]	G-Re1	G-Re1	C48H82O19	962.545	B1, B2, B3, B4	-2.61
73	5.35	1003.5427	1003.5500[M-H]⁻ 961.5477[M-H-Ac]⁻ 799.4829[M-H-Ac-glc]⁻ 637.4480[M-H-Ac-glc-glc]⁻ 475.3933[M-H-Ac-glc-glc-glc]	PPT1-glc-glc-glc-acetyl	6-acetyl ginsenoside-Rg3/isomer	C50H84O20	1004.5556	B1, B2, B3, B4	5.08
74	5.38	1009.5498	1009.5523[M+FA-H]⁻ 963.5516[M-H]⁻ 801.4981[M-H-glc]⁻ 639.4391[M-H-2glc]⁻ 477.4156[M-H-3glc]⁻	PPD4/PPT5-glc-glc-glc	Re-mo isomer	C48H84O19	964.5607	B1, B2, B3	1.35
75	5.44	885.482	885.4974[M-H]⁻ 841.4966[M-H-CO2]⁻ 799.4868[M-H-CO2-Ac]⁻ 679.4388[M-H-CO2-glc]⁻ 637.4320[M-H-CO2-Ac-glc]⁻ 619.4206[M-H-CO2-Ac-glc-H2O]⁻ 475.3780[M-H-CO2-Ac-2glc]⁻	glc-PPT1-glc-malonyl	mRg1 isomer	C45H74O17	886.4926	B1, B2, B3, B4	3.16
76	5.46	861.4864	861.4869[M+FA-H]⁻ 815.4838[M-H]⁻ 653.4208[M-H-glc]⁻ 491.3700[M-H-2glc]⁻	PG-glc-glc	Panajaponol A/isomer	C42H72O15	816.4871	B1, B2, B3, B4	-5.52
77	5.47	1191.6141	1191.6233[M-H]⁻ 1149.6145[M-H-Ac]⁻ 987.5473[M-H-Ac-glc]⁻ 945.5232[M-H-2Ac-glc]⁻ 783.4682[M-H-2Ac-2glc]⁻ 621.4388[M-H-2Ac-3glc]⁻ 459.3897[M-H-2Ac-4glc]⁻	PPD1-glc-glc-glc-glc-2acetyl	Diacetyl-G-Rb1 isomer	C58H96O25	1192.6241	B1, B2, B3	1.85
78	5.47	841.497	841.4979[M-H]⁻ 799.4883[M-H-Ac]⁻ 679.4424[M-H-glc]⁻ 637.4333[M-H-Ac-glc]⁻	glc-PPT1-glc-acetyl	noto-Rt/isomer	C44H74O15	842.5028	B1, B2, B3, B4	-2.38

			619.4212[M-H-Ac-glc-H2O]⁻ 571.4000 475.3784[M-H-Ac-2glc]⁻						
79	5.5	817.4849	817.4920[M-H]⁻ 655.4331[M-H-glc]⁻ 493.3411[M-H-2glc]⁻	PPT4-glc-glc	R1-Hy isomer	C42H74O15	818.5028	B3	3.67
80	5.54	931.5264	931.5268[M-H]⁻ 799.4871[M-H-xyl/ara]⁻ 637.4097[M-H-xyl/ara-glc]⁻ 637.4328[M-H-xyl/ara-glc]⁻ 475.3707 [M-H-xyl/ara-2glc]⁻	PPT1-glc-glc-ara/xyl	Re4 isomer/NG-R1 isomer	C47H80O18	932.5345	B1, B2, B3, B4	0.32
81	5.6	1107.5946	1107.5993[M-H]⁻ 945.5454[M-H-glc]⁻ 799.4826[M-H-glc-rha]⁻ 637.4536[M-H-glc-rha-glc]⁻ 475.3969[M-H-glc-glc-glc-rha]	glc-glc-PPT1-glc-rha	Yesanchinoside E/isomer	C54H92O23	1108.6029	B4	0.45
82	5.62	961.5394	961.5421[M-H]⁻ 799.4894[M-H-glc]⁻ 637.4306[M-H-2glc]⁻ 475.3684[M-H-3glc]⁻	PPT1-glc-glc-glc	Re1 isomer	C48H82O19	962.545	B1, B2, B3, B4	-2.29
83	5.66	653.4207	653.3400[M-H]⁻ 491.2908[M-H-glc]⁻	PG-glc	PG-F11 isomer	C36H62O10	654.4343	B2	8.88
84	5.76	883.5036	883.5039[M-H]⁻ 841.4901[M-H-Ac]⁻ 799.4997[M-H-2Ac]⁻ 637.4221[M-H-2Ac-glc]⁻ 475.3955[M-H-2Ac-2glc]⁻	PPT1-glc-glc-acetyl-acetyl	6',6''-di-O-acetyl Rg1 isomer	C46H76O16	884.5133	B1	2.15
85	5.77396	845.49298	845.4931[M+HCOO]⁻ 799.4878[M-H]⁻ 637.4344[M-H-Glc]⁻ 475.3796[M-H-2Glc]⁻	G-Rg1	G-Rg1	C42H72O14	800.4922	B1, B2, B3, B4	-4.25
86	5.8113	991.54974	991.5509[M+HCOO]⁻ 945.5451[M-H]⁻ 783.4931[M-H-Glc]⁻ 637.4339[M-H-Glc-Rha]⁻	G-Re	G-Re	C48H82O18	946.5501	B1, B2, B3, B4	-2.96
87	5.92365	811.48514	811.4875[M-H]⁻ 769.4769[M-H-C2H2O]⁻ 637.4332[M-H-C2H2O-Ara]⁻ 475.3822[M-H-	PPT1-glc-xyl/ara-acetyl	β-D-Glucopyranosid	C43H72O14	812.4922	B1, B2, B3, B4	-0.91

			C2H2O-Ara-Glc]$^-$ 457.3785[M-H-C2H2O-Ara-Glc-H2O]$^-$		e, (3β,6α,12β, 20*R*)-3,12,20-trihydroxydammar-24-en-6-yl 2-*O*-β-D-xylopyranosyl-, 6-acetate isomer				
88	6.03	841.4946	841.4992[M-H]$^-$ 799.4870[M-H-Ac]$^-$ 781.4783[M-H-Ac-H2O]$^-$ 637.4433[M-H-Ac-glc]$^-$ 571.3910 475.3696[M-H-Ac-2glc]$^-$	PPT1-glc-glc-acetyl	noto-Rt/isomer	C44H74O15	842.5028	B1, B2, B3, B4	0.48
89	6.07	931.5273	931.5295[M-H]$^-$ 799.4904[M-H-ara/xyl]$^-$ 637.4383[M-H-ara/xyl-glc]$^-$ 475.3807[M-H-ara/xyl-2glc]$^-$	PPT1-glc-glc-ara/xyl	Re4 isomer/NG-R1 isomer	C47H80O18	932.5345	B1, B4	-0.64
90	6.12	885.4868	885.4981[M-H]$^-$ 841.4957[M-H-CO2]$^-$ 799.4857[M-H-CO2-Ac]$^-$ 781.4785[M-H-CO2-Ac-H2O]$^-$ 637.4350[M-H-CO2-Ac-glc]$^-$ 475.3668[M-H-CO2-Ac-2glc]$^-$	glc-PPT1-glc-malonyl	mRg1 isomer	C45H74O17	886.4926	B1, B3, B4	-2.26
91	6.13	825.496	825.5019[M-H]$^-$ 783.4912[M-H-Ac]$^-$ 765.4817[M-H-Ac-H2O]$^-$ 621.4336[M-H-Ac-glc]$^-$ 459.3839[M-H-Ac-2glc]$^-$	PPD1-glc-glc-acetyl	Rs3 isomer	C44H74O14	826.5079	B1, B2, B3, B4	4.97
92	6.14	1031.535	1031.5723[M-H]$^-$ 989.5603[M-H-Ac]$^-$ 945.5464[M-H-Ac-CO2]$^-$ 799.4866[M-H-Ac-rha]$^-$ 637.4181[M-H-Ac-rha-glc]$^-$ 475.3799[M-H-Ac-CO2-rha-2glc]$^-$	PPT1-glc-glc-rha-malonyl	mRd isomer	C51H84O21	1032.5505	B1, B4	7.46

93	6.15	831.4744	831.4778[M+FA-H]⁻ 785.4726[M-H]⁻ 653.4287[M-H-xyl/ara]⁻ 491.3841[M-H-xyl/ara-glc]⁻	PG-glc-ara/xyl	M-R2/isomer	C41H70O14	786.4766	B2	-4.84
94	6.16	969.5454	969.5454[M-H-H2O]⁻ 927.5341[M-H-H2O-Ac]⁻ 909.5226[M-H-2H2O-Ac]⁻ 765.4705[M-H-H2O-Ac-glc]⁻ 621.4331[M-H-Ac-2glc]⁻ 459.3891[M-H-Ac-3glc]⁻	PPD1-3glc-acetyl	6'''-O-acetylgypenoside XVII isomer	C50H84O19	988.5607	B1, B2, B3, B4	-3.14
95	6.18	1123.5938	1123.5953[M-H]⁻ 961.5364[M-H-glc]⁻ 799.4912[M-H-2glc]⁻ 637.4347[M-H-3glc]⁻ 475.3801[M-H-4glc]⁻	PPT1-4glc	Notoginsenoside Fh7 isomer	C54H92O24	1124.5979	B2	-3.29
96	6.19	825.4991	825.5018[M-H]⁻ 783.495[M-H-Ac]⁻ 765.4748[M-H-Ac-H2O]⁻ 679.4401[M-H-rha]⁻ 637.4332[M-H-Ac-rha]⁻ 619.4226[M-H-Ac-rha-H2O]⁻ 571.4032 475.3756[M-H-Ac-rha-glc]⁻	acetyl-PPT1-glc-rha	β-D-Glucopyranoside, (3β,6α,12β,20*R*)-3,12,20-trihydroxydammar-24-en-6-yl 2-*O*-(6-deoxy-α-L-mannopyranosyl)-, 6-acetate isomer	C44H74O14	826.5079	B1, B2, B3, B4	1.21

97	6.2	799.4847	799.4860[M-H]⁻ 637.4330[M-H-glc]⁻ 475.3830[M-H-2glc]⁻	PPT1-2glc	Rg1 isomer	C42H72O14	800.4922	B1, B2	-0.38
98	6.26	1029.5618	1029.5673[M-H]⁻ 987.5567[M-H-Ac]⁻ 945.5432[M-H-2Ac]⁻ 783.5020[M-H-Ac-Ac-glc]⁻ 621.4281[M-H-Ac-Ac-2glc]⁻ 459.3810[M-H-Ac-Ac-3glc]⁻	PPD1-glc-glc-glc-acetyl-acetyl	Diacetyl-G-Rd isomer	C52H86O20	1030.5712	B1	1.55
99	6.29	679.4419	679.4447[M-H]⁻ 637.4311[M-H-Ac]⁻ 619.4212[M-H-Ac-H2O]⁻ 475.3802[M-H-Ac-glc]⁻	PPT1-glc-acetyl	20R-ginsenoside Rh1 6‘-acetate isomer	C38H64O10	680.4499	B1, B2, B3, B4	0.29
100	6.29326	955.492	957.5016[M-H]⁻ 956.4995[M-H]⁻ 794.4441[M-H-Glc]⁻	G-Ro	G-Ro	C48H76O19	956.4981	B1, B2, B3, B4	-1.78
101	6.32	665.3858	665.3879[M+FA-H]⁻ 619.4193[M-H]⁻ 457.3288[M-H-glc]⁻	PPT2-glc	G-Rh4 isomer	C36H60O8	620.4288	B1, B2, B3, B4	2.74
102	6.37	845.4893	845.4843 799.4884 653.4308 491.3699	PG-F11 isomer	PG-F11 isomer	C42H72O14	800.4922	B1, B2	-5.00
103	6.43	931.5232	931.5243[M-H]⁻ 799.4716[M-H-ara/xyl]⁻ 637.4242[M-H-ara/xyl-glc]⁻ 475.3887[M-H-ara/xyl-2glc]⁻	PPT1-glc-glc-ara/xyl	Re4 isomer/NG-R1 isomer	C47H80O18	932.5345	B1, B2, B3, B4	3.76
104	6.5	831.4723	831.4686[M+FA-H]⁻ 785.4759[M-H]⁻ 653.4282[M-H-xyl/ara]⁻ 491.3705[M-H-xyl/ara-glc]⁻	PG-glc-ara/xyl	M-R2/isomer	C41H70O14	786.4766	B1, B2, B3	-9.04

105	6.51	815.478	815.4735[M-H]⁻ 653.4704[M-H-glc]⁻ 491.3819[M-H-2glc]⁻	PG-glc-glc	Panajaponol A/isomer	C42H72O15	816.4871	B1, B2, B4	1.59
106	6.51	811.4834	811.4895[M-H]⁻ 769.4773[M-H-Ac]⁻ 637.4368[M-H-Ac-ara/xyl]⁻ 475.3959[M-H-Ac-ara/xyl-glc]⁻	PPT1-glc-ara/xyl-acetyl	β-D-Glucopyranoside, (3β,6α,12β,20*R*)-3,12,20-trihydroxydammar-24-en-6-yl 2-*O*-β-D-xylopyranosyl-, 6-acetate isomer	C43H72O14	812.4922	B1, B2, B3	1.23
107	6.52	1093.5808	1093.5847[M-H]⁻ 961.5435[M-H-xyl/ara]⁻ 931.5202[M-H-glc]⁻ 799.4910[M-H-glc-xyl/ara]⁻ 637.4374[M-H-2glc-xyl/ara]⁻ 475.3764[M-H-3glc-xyl/ara]⁻	PPT1-glc-glc-glc-xyl/ara	20S-sanchirhinosides A6 isomer	C53H90O23	1094.5873	B1, B2, B4	-1.19
108	6.54	1003.5454	1003.5481[M-H]⁻ 961.5396[M-H-Ac]⁻ 799.4832[M-H-Ac-glc]⁻ 637.4175[M-H-Ac-glc-glc]⁻ 475.3772[M-H-Ac-glc-glc-glc]	PPT1-glc-glc-glc-acetyl	6-acetyl ginsenoside-Rg3/isomer	C50H84O20	1004.5556	B1, B2, B4	2.39
109	6.54	769.4724	769.4772[M-H]⁻ 637.4407[M-H-xyl/ara]⁻ 475.3722[M-H-xyl/ara-glc]⁻	PPT1-glc-xyl/ara	F3 isomer	C41H70O13	770.4816	B1, B2, B3	1.82

110	6.64	947.522	947.5277[M+FA-H]$^-$ 901.5203[M-H]$^-$ 769.4726[M-H-xyl/ara]$^-$ 637.4319[M-H-2xyl/ara]$^-$ 475.3788[M-H-2xyl/ara-glc]$^-$	PPT1-glc-xyl/ara-xyl/ara	chikusetsusaponin LM2 isomer	C46H78O17	902.5239	B1, B2, B3	-4.66
111	6.72	961.5359	961.5325[M+FA-H]$^-$ 915.5325[M-H]$^-$ 753.4576[M-H-glc]$^-$ 607.4219[M-H-glc-rha]$^-$ 475.3849[M-H-glc-rha-xyl/ara]$^-$	PPT1-glc-rha-xyl/ara	β-D-Glucopyranoside, (3β,6α,12β)-3,12-dihydroxy-20-(β-D-xylopyranosyloxy)dammar-24-en-6-yl 2-*O*-(6-deoxy-α-L-mannopyranosyl)- isomer	C47H80O17	916.5396	B1, B3	-0.76
112	6.73	1149.6066	1149.6089[M-H]$^-$ 1107.6009[M-H-Ac]$^-$ 1089.5892[M-H-Ac-H2O]$^-$ 945.5461[M-H-Ac-glc]$^-$ 783.4993[M-H-Ac-2glc]$^-$ 765.4829[M-H-Ac-2glc-H2O]$^-$ 621.4365[M-H-Ac-3glc]$^-$ 459.3825[M-H-Ac-4glc]$^-$	PPD1-glc-glc-glc-glc-acetyl	Q-R1 isomer	C56H94O24	1150.6135	B1, B2, B3, B4	-0.78
113	6.74285	925.48176	925.4842[M-H]$^-$	CS-IV	CS-IV	C47H74O18	926.4875	B1, B2, B3, B4	-2.23

114	6.75	1281.6478	1149.6117[M-H-xyl/ara]‾ 1107.5936[M-H-xyl/ara-Ac]‾ 1089.5892[M-H-xyl/ara-Ac-H2O]‾ 945.5520[M-H-xyl/ara-Ac-glc]‾ 783.4989[M-H-xyl/ara-Ac-2glc]‾ 621.4477[M-H-xyl/ara-Ac-3glc]‾ 459.3801[M-H-xyl/ara-Ac-4glc]‾	PPD1-glc-glc-glc-glc-acetyl-xyl/ara	O-acetyl-G-Ra3 isomer	C61H102O28	1282.6558	B2, B3, B4	0.16
115	6.76	815.4796	815.4854[M+FA-H]‾ 769.4784[M-H]‾ 607.4241[M-H-glc]‾ 475.3759[M-H-glc-xyl/ara]‾	PPT1-glc-xyl/ara	20(S)-NG-R2 isomer	C41H70O13	770.4816	B1, B2, B3	-5.98
116	6.77	679.4418	679.4397[M-H]‾ 637.4365[M-H-Ac]‾ 619.4195[M-H-Ac-H2O]‾ 475.3857[M-H-Ac-glc]‾	PPT1-glc-acetyl	20R-ginsenoside Rh1 6‘-acetate isomer	C38H64O10	680.4499	B1, B2, B3, B4	0.44
117	6.79	1193.5966	1193.5988[M-H]‾ 1149.6080[M-H-CO2]‾ 1107.5995[M-H-CO2-Ac]‾ 1089.5890[M-H-CO2-Ac-H2O]‾ 945.5485[M-H-CO2-Ac-glc]‾ 783.4917[M-H-CO2-Ac-2glc]‾ 621.4372[M-H-CO2-Ac-3glc]‾ 459.3923[M-H-CO2-Ac-4glc]‾	PPD1-glc-glc-glc-glc-malonyl	Ginsenoside Ma-Rb1 isomer	C57H94O26	1194.6033	B1, B2, B3, B4	-0.92
118	6.81	1153.6172	1153.6211[M+FA-H]‾ 1107.5999[M-H]‾ 945.5462[M-H-glc]‾ 783.4902[M-H-glc-glc]‾ 621.4482[M-H-glc-glc-glc] 459.3881[M-H-glc-glc-glc-glc]	PPD1-glc-glc-glc-glc	Rb1 isomer	C54H92O23	1108.6029	B1, B2, B3	-4.33
119	6.95	825.5009	825.4978[M-H]‾ 783.4903[M-H-Ac]‾ 637.4395[M-H-Ac-rha]‾ 475.3888[M-H-Ac-rha-glc]‾	PPT1-glc-rha-acetyl	β-D-Glucopyranoside, (3β,6α,12β,	C44H74O14	826.5079	B1, B2, B4	-0.97

					20*R*)-3,12,20-trihydroxydammar-24-en-6-yl 2-*O*-(6-deoxy-α-L-mannopyranosyl)-, 6-acetate isomer				
120	6.98	1149.6074	1149.6056[M-H]$^-$ 1107.5958[M-H-Ac]$^-$ 1089.5855[M-H-Ac-H2O]$^-$ 987.5480[M-H-glc]$^-$ 945.5435[M-H-Ac-glc]$^-$ 927.5342[M-H-Ac-glc-H2O]$^-$ 783.4877[M-H-Ac-2glc] 621.4405[M-H-Ac-3glc]$^-$ 459.3881[M-H-Ac-4glc]$^-$	glc-PPD1-glc-glc-glc-acetyl	Q-R1 isomer	$C_{56}H_{94}O_{24}$	1150.6135	B1, B2, B3, B4	-1.48
121	7.04	769.473	769.4780[M-H]$^-$ 637.4293[M-H-ara/xyl]$^-$ 619.4254[M-H-ara/xyl-H2O]$^-$ 475.3819[M-H-ara/xyl-glc]$^-$	PPT1-glc-xyl/ara	F3 isomer	$C_{41}H_{70}O_{13}$	770.4816	B1, B2, B3, B4	1.04
122	7.08036	1119.5959	1119.5964 1077.5883 1059.5768	R-s2isomer	R-s2 isomer	$C_{55}H_{92}O_{23}$	1120.6029	B1, B2, B3, B4	-0.75
123	7.13	1077.5817	1077.5881[M-H]$^-$ 945.5424[M-H-xyl/ara]$^-$ 783.4820[M-H-xyl/ara-glc]$^-$ 621.4493[M-H-xyl/ara-2glc]$^-$ 459.3929[M-H-xyl/ara-3glc]$^-$	xyl/ara-PPD1-glc-glc-glc	Rb2 isomer	$C_{53}H_{90}O_{22}$	1078.5924	B1, B2, B3, B4	2.69

124	7.13498	793.43963	793.4404[M-H]⁻ 631.3859[M-H-Glc]⁻ 613.3744[M-H-glc-H2O]⁻ 569.3856[M-H-glc-CO2]⁻ 455.3530[M-H-glc-glcA]⁻	CS-Iva	CS-Iva	C42H66O14	794.4453	B2, B3, B4	-2.68
125	7.17	915.5306	915.5325[M-H]⁻ 783.4918[M-H-xyl/ara]⁻ 637.4339[M-H-xyl/ara-rha]⁻ 475.3944[M-H-xyl/ara-rha-glc]⁻	PPT1-glc-rha-xyl/ara	β-D-Glucopyranoside, (3β,6α,12β)-3,12-dihydroxy-20-(β-D-xylopyranosyloxy)dammar-24-en-6-yl 2-*O*-(6-deoxy-α-L-mannopyranosyl)- isomer	C47H80O17	916.5396	B1, B2, B3, B4	1.31
126	7.18	1163.5858	1163.5887[M-H]⁻ 1119.5991[M-H-CO2]⁻ 1077.5834[M-H-CO2-Ac]⁻ 945.5411[M-H-CO2-Ac-xyl/ara]⁻ 915.5281[M-H-CO2-Ac-glc]⁻ 783.4903[M-H-CO2-Ac-xyl/ara-glc]⁻ 765.4694[M-H-CO2-Ac-xyl/ara-glc-H2O]⁻ 621.4272[M-H-CO2-Ac-xyl/ara-2glc]⁻ 459.3799[M-H-CO2-Ac-xyl/ara-3glc]⁻	PPD1-glc-glc-glc-xyl/ara-malonyl	m-floral-Rc4 isomer	C56H92O25	1164.5928	B1, B2, B3	-0.69

127	7.24	771.4855	771.4855[M-H]⁻ 639.4417[M-H-xyl/ara]⁻ 477.3792[M-H-xyl/ara-glc]⁻	PPD4/PPT5-glc-xyl/ara	β-D-Glucopyranoside, (3β,6α,12β)-3,12,25-trihydroxydammaran-6-yl 2-*O*-β-D-xylopyranosyl-isomer	C41H72O13	772.4973	B3, B4	5.18
128	7.25	1007.5434	1007.5456[M+FA-H]⁻ 961.5386[M-H]⁻ 799.5002[M-H-glc]⁻ 637.4392[M-H-glc-glc]⁻ 475.3713[M-H-glc-glc-glc]⁻	PPT1-glc-glc-glc	20-glu-G-Rf isomer	C48H82O19	962.545	B1, B2, B3, B4	-1.46
129	7.26	815.4806	815.4840[M+FA-H]⁻ 769.4783[M-H]⁻ 637.4345[M-H-xyl/ara]⁻ 475.3795[M-H-xyl/ara-glc]⁻	PPT1-glc-xyl/ara	20(S)-NG-R2 isomer	C41H70O13	770.4816	B1, B2, B3, B4	-5.85
130	7.27	679.4408	679.4489[M-Ac]⁻ 637.4302[M-H]⁻ 475.3788[M-H-glc]⁻	PPT1-glc-acetyl	20R-ginsenoside Rh1 6'-acetate isomer	C38H64O10	680.4499	B1, B2, B3, B4	1.91
131	7.36	841.4969	841.5016[M-H]⁻ 805.4004[M-H-H2O]⁻ 799.4845[M-H-Ac]⁻ 781.4655[M-H-Ac-H2O]⁻ 637.4320[M-H-Ac-glc]⁻ 475.3926[M-H-Ac-2glc]⁻	glc-PPT1-glc-acetyl	noto-Rt/isomer	C44H74O15	842.5028	B1, B2, B3, B4	-2.26
132	7.41	1119.5978	1119.5997[M-H]⁻ 1077.5891[M-H-Ac]⁻ 1059.5795[M-H-Ac-H2O]⁻ 945.5502[M-H-Ac-xyl/ara]⁻ 783.4929[M-H-Ac-xyl/ara-glc]⁻ 765.4853[M-H-Ac-xyl/ara-glc-H2O]⁻ 621.4379[M-	PPD1-glc-glc-glc-xyl/ara-acetyl	Rs1 isomer	C55H92O23	1120.6029	B1, B2, B3, B4	-2.41

			H-Ac-xyl/ara-2glc]⁻ 459.3871[M-H-Ac-xyl/ara-3glc]⁻						
133	7.48	1163.589	1163.5697[M-H]⁻ 1119.5992[M-HCOO]⁻ 1077.5875[M-H-HCOO-Ac]⁻ 1059.5772[M-H-HCOO-Ac-H2O]⁻ 945.5770[M-H-HCOO-Ac-xyl/ara]⁻ 783.4948[M-H-HCOO-Ac-xyl/ara-glc]⁻ 765.4912[M-H-HCOO-Ac-xyl/ara-glc-H2O]⁻ 621.4249[M-H-HCOO-Ac-xyl/ara-2glc]⁻ 459.3936[M-H-HCOO-Ac-xyl/ara-3glc]⁻	PPD1-glc-glc-glc-xyl/ara-malonyl	Malonyl-ginsenosideRc isomer	C56H92O25	1164.5928	B1, B2, B3	-3.44
134	7.49	1059.5786	1059.5801[M+FA-H]⁻ 945.5463[M-H-C4H4O]⁻ 783.5000[M-H-C4H4O-glc]⁻ 621.4579[M-H-C4H4O-2glc]⁻	PPD1-glc-glc-glc-butenoyl	Quinquenoside I isomer	C52H86O19	1014.5763	B1, B2, B3, B4	-4.83
135	7.52	1149.6075	1149.6048 1107.5995 1089.5870 1077.5993 987.5319 945.5390 783.4874 621.4255	Q-R1isomer	Q-R1 isomer	C56H94O24	1150.6135	B1, B2, B3, B4	-1.57
136	7.54	1077.5881	1077.5852[M-H]⁻ 945.5452[M-H-xyl/ara]⁻ 915.5419[M-H-glc]⁻ 783.4926[M-H-glc-xyl/ara]⁻ 765.4782[M-H-glc-xyl/ara-H2O]⁻ 621.4221[M-H-2glc-xyl/ara]⁻ 459.3994[M-H-3glc-xyl/ara]⁻	xyl/ara-PPD1-glc-glc-glc	Rb2 isomer	C53H90O22	1078.5924	B1, B2, B3, B4	-3.25
137	7.59	825.4986	825.5021[M-H]⁻ 783.4927[M-H-Ac]⁻ 621.4308[M-H-Ac-glc]⁻ 459.3853[M-H-Ac-2glc]⁻	PPD1-glc-glc-acetyl	Rs3 isomer	C44H74O14	826.5079	B1, B3	1.82
138	7.65	931.5248	931.5227[M-H]⁻ 799.4649[M-H-xyl/ara]⁻ 781.4975[M-H-xyl/ara-H2O]⁻ 637.3980[M-H-xyl/ara-glc]⁻ 475.3746[M-H-xyl/ara-2glc]⁻	PPT1-glc-glc-ara/xyl	Re4 isomer/NG-R1 isomer	C47H80O18	932.5345	B1, B2, B3, B4	2.04

139	7.69	829.4938	829.4938[M+FA-H]⁻ 783.4847[M-H]⁻ 621.4377[M-H-glc]⁻ 475.3752[M-H-glc-rha]⁻	PPT1-rha-glc	Rg2 isomer	C42H72O13	784.4973	B2, B3, B4	6.13
140	7.71	961.5355	961.5419[M-H]⁻ 799.4892[M-H-glc]⁻ 637.4323[M-H-2glc]⁻ 475.3792[M-H-3glc]⁻	PPT1-glc-glc-glc	Re1 isomer	C48H82O19	962.545	B1, B2, B3, B4	1.77
141	7.74	1193.5962	1193.5947[M-H]⁻ 1149.6054[M-H-CO2]⁻ 1107.6083[M-H-CO2-Ac]⁻ 987.5424[M-H-CO2-glc]⁻ 945.5439[M-H-CO2-Ac-glc]⁻ 783.5100[M-H-CO2-Ac-2glc]⁻ 621.4362[M-H-CO2-Ac-3glc]⁻	PPD1-glc-glc-glc-glc-malonyl	Ginsenoside Ma-Rb1 isomer	C57H94O26	1194.6033	B1, B2, B3	-0.59
142	7.74	987.5467	987.5421[M-H]⁻ 945.5394[M-H-Ac]⁻ 927.5360[M-H-Ac-H2O]⁻ 783.4935[M-H-Ac-glc]⁻ 621.4373[M-H-Ac-2glc]⁻ 459.3839[M-H-Ac-3glc]⁻	glc-PPD1-2glc-acetyl	6'''-O-acetylgypenoside XVII isomer	C50H84O19	988.5607	B1, B2, B3, B4	6.28
143	7.76	679.4408	679.4396[M-Ac]⁻ 637.4322[M-H]⁻ 619.4113[M-H-H2O]⁻ 475.3744[M-H-Ac-glc]⁻	PPT1-glc-acetyl	20R-ginsenoside Rh1 6‘-acetate isomer	C38H64O10	680.4499	B1, B2, B3, B4	1.91
144	7.79823	815.47898	815.4803[M-H]⁻ 653.4264[M-H-glc]⁻ 491.3828[M-H-glc-glc]⁻	PG(母核)-2glc 或如图	Panajaponol A/isomer	C42H72O15	816.4871	B1, B2, B3, B4	0.39
145	7.92	807.491	807.4910[M-H]⁻ 765.4379[M-H-Ac]⁻ 603.4843[M-H-Ac-glc]⁻ 441.3493[M-H-Ac-2glc]⁻	PPD2-glc-glc-acetyl	20Z-ginsenoside-Rs4 isomer	C44H72O13	808.4973	B1, B2, B3	-1.86
146	7.97	1119.5946	1119.5988[M-H]⁻ 1077.5883[M-H-Ac]⁻ 1059.5782[M-H-Ac-H2O]⁻ 945.5323[M-H-Ac-xyl/ara]⁻ 783.4965[M-H-Ac-xyl/ara-glc]⁻ 621.4344[M-H-Ac-xyl/ara-2glc]⁻ 459.3851[M-H-Ac-xyl/ara-3glc]⁻	PPD1-glc-glc-glc-xyl/ara-acetyl	Rs1 isomer	C55H92O23	1120.6029	B1, B2, B3, B4	0.45

147	7.97	1059.5706	1059.5786[M+FA-H]⁻ 945.5333[M-H-C4H4O]⁻ 927.5494[M-H-C4H4O-H2O]⁻ 765.4869[M-H-C4H4O-glc-H2O]⁻ 621.4231[M-H-C4H4O-2glc]⁻ 459.3881[M-H-C4H4O-3glc]⁻	PPD1-glc-glc-glc-butenoyl	Quinquenoside I isomer	C52H86O19	1014.5763	B2, B3	-4.54
148	8.05	799.4838	799.4761 637.4593 475.3756	PPT1-2glc	Rg1 isomer	C42H72O14	800.4922	B4	0.75
149	8.09	987.5542	987.5578[M-H]⁻ 945.5469[M-H-Ac]⁻ 927.5306[M-H-Ac-H2O]⁻ 841.4994[M-H-rha]⁻ 799.5353[M-H-Ac-rha]⁻ 637.4142[M-H-Ac-rha-glc]⁻ 475.3844[M-H-Ac-rha-glc-glc]⁻	PPT1-glc-glc-rha-acetyl	Pseudoginsenoside Rs1/isomer	C50H84O19	988.5607	B1, B2, B3, B4	-1.32
150	8.19	807.493	807.4493[M-H]⁻ 765.4404[M-H-Ac]⁻ 603.3835[M-H-Ac-glc]⁻ 441.3430[M-H-Ac-2glc]⁻	PPD2-glc-glc-acetyl	20Z-ginsenoside-Rs4 isomer	C44H72O13	808.4973	B1, B2, B4	-4.33
151	8.2	825.4978	825.5012[M-H]⁻ 783.4893[M-H-Ac]⁻ 621.4283[M-H-Ac-glc]⁻ 459.3637[M-H-Ac-2glc]⁻	PPD1-glc-glc-acetyl	Rs3 isomer	C44H74O14	826.5079	B1, B4	2.79
152	8.21	1031.546	1031.5453[M-H]⁻ 987.5555[M-H-CO2]⁻ 945.5457[M-H-CO2-Ac]⁻ 927.5351[M-H-CO2-Ac-H2O]⁻ 783.4925[M-H-CO2-Ac-glc]⁻ 765.4826[M-H-CO2-Ac-glc-H2O]⁻ 621.4347[M-H-CO2-Ac-glc-glc]⁻ 459.3807[M-H-CO2-Ac-glc-glc-glc]⁻	PPD1-glc-glc-glc-malonyl	Malonyl-ginsenoside Re isomer	C51H84O21	1032.5505	B1, B2, B3, B4	-3.20
153	8.25	841.4976	841.4957[M-H]⁻ 799.4828[M-H-Ac]⁻ 781.4806[M-H-Ac-H2O]⁻ 679.4316[M-H-glc]⁻ 637.4337[M-H-Ac-glc]⁻ 619.4211[M-H-Ac-glc-H2O]⁻ 475.3762[M-H-Ac-2glc]⁻	glc-PPT1-glc-acetyl	noto-Rt/isomer	C44H74O15	842.5028	B1, B2, B3, B4	-3.09

154	8.26	1191.6112	1191.6156[M-H]⁻ 1149.6241[M-H-Ac]⁻ 1131.6143[M-H-Ac-H2O]⁻ 987.5597[M-H-Ac-glc]⁻ 945.5473[M-H-2Ac-glc]⁻ 927.5389[M-H-2Ac-glc-H2O]⁻ 783.4905[M-H-2Ac-2glc]⁻ 765.4808[M-H-2Ac-2glc-H2O]⁻ 621.4408[M-H-2Ac-3glc]⁻ 459.3743[M-H-2Ac-4glc]⁻	PPD1-glc-glc-glc-glc-acetyl-acetyl	Diacetyl-G-Rb1 isomer	C58H96O25	1192.6241	B1, B2, B3, B4	4.28
155	8.28	863.4994	863.5102[M+FA-H]⁻ 817.4886[M-H]⁻ 655.4433[M-H-glc]⁻ 493.3639[M-H-2glc]⁻	PPT4-glc-glc	20-S-Rf3/ isomer	C42H74O15	818.5028	B1, B2, B3, B4	7.83
156	8.31	945.5443	945.5464[M-H]⁻ 783.4924[M-H-glc]⁻ 621.4346[M-H-2glc]⁻ 459.3801[M-H-3glc]⁻	PPD1-glc-glc-glc	Rd isomer	C48H82O18	946.5501	B1, B2, B3, B4	-2.12
157	8.34	829.4948	829.4947[M+FA-H]⁻ 783.4879[M-H]⁻ 637.4256[M-H-rha]⁻ 619.4134[M-H-rha-H2O]⁻ 475.3664[M-H-rha-glc]⁻	PPT1-rha-glc	Rg2 isomer	C42H72O13	784.4973	B1, B2, B3, B4	2.04
158	8.4	987.5528	987.5528[M-H]⁻ 945.5436[M-H-Ac]⁻ 927.5336[M-H-Ac-H2O]⁻ 765.4803[M-H-Ac-H2O-glc]⁻ 621.4381[M-H-Ac-2glc]⁻ 459.3827[M-H-Ac-3glc]⁻	glc-PPD1-2glc-acetyl	6'''-O-acetylgypenoside XVII isomer	C50H84O19	988.5607	B1, B2, B3, B4	0.10
159	8.52	945.5401	945.5413[M-H]⁻ 783.4842[M-H-glc]⁻ 621.4326[M-H-2glc]⁻ 459.3746[M-H-3glc]⁻	PPD1-glc-glc-glc	Rd isomer	C48H82O18	946.5501	B1, B2, B3, B4	2.33
160	8.53	929.5466	929.5466[M-H]⁻ 767.4818[M-H-glc]⁻ 605.4286[M-H-2glc]⁻ 459.3959[M-H-2glc-rha]⁻	PPD1-glc-glc-rha	Gynosaponin III isomer	C48H82O17	930.5552	B1, B3, B4	0.86
161	8.6	1031.546	1031.5472[M-H]⁻ 987.5576[M-H-CO2]⁻ 945.5482[M-H-CO2-Ac]⁻ 927.5375[M-H-CO2-Ac-H2O]⁻ 783.4942[M-H-CO2-Ac-glc]⁻ 765.4810[M-	PPD1-glc-glc-glc-malonyl	Malonyl-ginsenoside Re isomer	C51H84O21	1032.5505	B1, B2, B3, B4	-3.20

			H-CO2-Ac-glc-H2O]⁻ 621.4350[M-H-CO2-Ac-2glc]⁻ 459.3804[M-H-CO2-Ac-3glc]⁻						
162	8.63	1119.5967	1119.6012[M-H]⁻ 1077.5995[M-H-Ac]⁻ 1059.5741[M-H-Ac-H2O]⁻ 945.5616[M-H-Ac-xyl/ara]⁻ 783.4758[M-H-Ac-xyl/ara-glc]⁻ 621.4350[M-H-Ac-xyl/ara-2glc]⁻ 459.3805[M-H-Ac-xyl/ara-3glc]⁻	PPD1-glc-glc-glc-xyl/ara-acetyl	Rs2 isomer	C55H92O23	1120.6029	B2, B3	-1.43
163	8.69	783.4893	783.4907[M-H]⁻ 621.4342[M-H-glc]⁻ 459.3858[M-H-2glc]⁻	PPD1-glc-glc	20(S)-G-Rg3 isomer	C42H72O13	784.4973	B1, B2, B3	0.26
164	8.74	799.4816	799.4761 637.4710 475.3837	PPT1-2glc	Rg1 isomer	C42H72O14	800.4922	B1, B2, B3, B4	3.50
165	8.75	961.5357	961.5382[M-H]⁻ 799.4880[M-H-glc]⁻ 781.4725[M-H-glc-H2O]⁻ 679.4304 637.4469[M-H-2glc]⁻ 475.3603[M-H-3glc]⁻	PPT1-3glc	Re1 isomer	C48H82O19	962.545	B1, B2, B3, B4	1.56
166	8.77	831.4737	831.4722[M+FA-H]⁻ 785.4706[M-H]⁻ 653.4274[M-H-xyl/ara]⁻ 491.3850[M-H-xyl/ara-glc]⁻	PG-glc-ara/xyl	M-R2/isomer	C41H70O14	786.4766	B1, B2, B3, B4	-2.29
167	8.79	987.5528	987.5561[M-H]⁻ 945.5443[M-H-Ac]⁻ 783.4872[M-H-Ac-glc]⁻ 765.4804[M-H-Ac-glc-H2O]⁻ 621.4402[M-H-Ac-glc-glc]⁻ 459.4001[M-H-Ac-glc-glc-glc]⁻	glc-PPD1-2glc-acetyl	6'''-O-acetylgypenoside XVII isomer	C50H84O19	988.5607	B1, B2, B3, B4	0.10
168	8.81	945.5391	945.5414[M-H]⁻ 783.5013[M-H-glc]⁻ 621.4222[M-H-2glc]⁻ 459.3891[M-H-3glc]⁻	PPD1-glc-glc-glc	Rd isomer	C48H82O18	946.5501	B1, B2, B3, B4	3.38
169	8.9	977.5219	977.5219[M+FA-H]⁻ 931.5304[M-H]⁻ 799.4721[M-H-xyl/ara]⁻ 637.4305[M-H-xyl/ara-glc]⁻ 475.3873[M-H-xyl/ara-2glc]⁻	PPT1-glc-glc-xyl/ara	Re4 isomer/NG-R1 isomer	C47H80O18	932.5345	B1, B2, B3, B4	-3.97

170	8.98	913.5198	913.5173[M+FA-H]⁻ 867.5149[M-H]⁻ 781.4694[M-H-C4H4O-H2O]⁻ 799.4615[M-H-C4H4O]⁻ 637.4379[M-H-C4H4O-glc]⁻ 619.4215[M-H-C4H4O-glc-H2O]⁻ 475.38249[M-H-C4H4O-2glc]⁻	PPT1-glc-glc-butenoyl	koryoginsenoside-R1/isomer	C46H76O15	868.5184	B1, B2, B3, B4	-4.96
171	9.06	1031.548	1031.5461[M-H]⁻ 987.5568[M-H-CO2]⁻ 945.5492[M-H-CO2-Ac]⁻ 783.4991[M-H-CO2-Ac-glc]⁻ 621.4164[M-H-CO2-Ac-glc-glc]⁻ 459.3901[M-H-CO2-Ac-glc-glc-glc]⁻	PPD1-glc-glc-glc-malonyl	Malonyl-ginsenoside Re isomer	C51H84O21	1032.5505	B1, B2, B3, B4	-5.14
172	9.06	987.553	987.5548[M-H]⁻ 945.5493[M-H-Ac]⁻ 927.5308[M-H-Ac-H2O]⁻ 783.4851[M-H-Ac-glc]⁻ 621.4411[M-H-Ac-glc-glc]⁻ 459.3694[M-H-Ac-glc-glc-glc]⁻	PPD1-glc-glc-glc-acetyl	6'''-O-acetylgypenoside XVII isomer	C50H84O19	988.5607	B1, B2, B3, B4	-0.10
173	9.07	929.4474	929.4868[M+FA-H]⁻ 883.4996[M-H]⁻ 799.4841[M-H-2Ac]⁻ 637.4349[M-H-2Ac-glc]⁻ 475.3790[M-H-2Ac-2glc]⁻	PPT1-glc-glc-acetyl-acetyl	6',6''-di-O-acetyl Rg1 isomer	C46H76O16	884.5133	B1, B2, B3, B4	6.68
174	9.07504	1239.635	1107.5982[M-H-Xyl]⁻ 945.5493[M-H-Xyl-Glc]⁻	NG-R4	NG-R4	C55H100O30	1240.6299	B1, B2, B3, B4	-10.00
175	9.0897	845.4926	845.4900[M+HCOO]⁻ 799.4860[M-H]⁻ 637.4344[M-H-Glc]⁻ 475.3802[M-H-2Glc]⁻	20(S)-G-Rf-1a	20(S)-G-Rf-1a	C42H72O14	800.4922	B1, B2, B3, B4	-2.00
176	9.09026	799.48686	799.4891[M-H]⁻ 637.4353[M-H-Glc]⁻ 475.3801[M-H-2Glc]⁻	20(R)-G-Rf	20(R)-G-Rf	C42H72O14	800.4922	B1, B2, B3, B4	-3.07
177	9.09026	799.48686	799.4891[M-H]⁻ 637.4353[M-H-Glc]⁻ 475.3801[M-H-2Glc]⁻	20(S)-G-Rf	20(S)-G-Rf	C42H72O14	800.4922	B1, B2, B3, B4	-3.07

178	9.15	913.4741	913.4822[M+FA-H]$^-$ 867.5104[M-H]$^-$ 799.4850[M-H-C4H4O]$^-$ 637.4326[M-H-C4H4O-glc]$^-$ 475.3892[M-H-C4H4O-2glc]$^-$	PPT1-glc-glc-butenoyl	koryoginsenoside-R1/isomer	C46H76O15	868.5184	B1, B2, B3, B4	0.23
179	9.4	987.553	987.5526[M-H]$^-$ 945.5483[M-H-Ac]$^-$ 783.4857[M-H-Ac-glc]$^-$ 621.4369[M-H-Ac-glc-glc]$^-$ 459.3979[M-H-Ac-glc-glc-glc]$^-$	glc-PPD1-2glc-acetyl	6'''-O-acetylgypenoside XVII isomer	C50H84O19	988.5607	B1, B2, B3, B4	-0.10
180	9.4	929.54	929.5140[M-H]$^-$ 783.5028[M-H-rha]$^-$ 621.4519[M-H-rha-glc]$^-$ 459.3665[M-H-rha-2glc]$^-$	PPD1-glc-glc-rha	Gynosaponin III isomer	C48H82O17	930.5552	B2, B4	7.96
181	9.43	931.5298	931.5312[M-H]$^-$ 769.4665[M-H-glc]$^-$ 637.4415[M-H-glc-xyl/ara]$^-$ 475.4008[M-H-2glc-xyl/ara]$^-$	PPT1-glc-glc-ara/xyl	Re4 isomer/NG-R1 isomer	C47H80O18	932.5345	B1, B2, B3, B4	-3.33
182	9.52	1269.65	1107.6022[M-H-glc]$^-$ 1089.5601[M-H-glc-H2O]$^-$ 945.5489[M-H-glc-glc]$^-$ 783.4800[M-H-glc-glc-glc]$^-$ 621.4350[M-H-glc-glc-glc-glc]$^-$ 459.3861[M-H-glc-glc-glc-glc-glc]	PPD1-glc-glc-glc-glc-glc	Ra0 isomer	C60H102O28	1270.6558	B1, B2, B3, B4	-1.58
183	9.59	769.4746	769.4762[M-H]$^-$ 637.4310[M-H-xyl/ara]$^-$ 475.3799[M-H-xyl/ara-glc]$^-$	PPT1-glc-xyl/ara	G-F3 isomer	C41H70O13	770.4816	B1, B2, B3	-1.04
184	9.66	947.5235	947.5275[M+FA-H]$^-$ 901.5202[M-H]$^-$ 769.4802[M-H-xyl/ara]$^-$ 637.4297[M-H-2xyl/ara]$^-$ 475.3774[M-H-2xyl/ara-glc]$^-$	PPT1-glc-xyl/ara-xyl/ara	chikusetsusaponin LM2 isomer	C46H78O17	902.5239	B3, B4	-4.55
185	9.67	845.4901	845.4864[M+FA-H]$^-$ 799.4923[M-H]$^-$ 637.4226[M-H-glc]$^-$ 475.3758[M-H-2glc]$^-$	PPT1-glc-glc	Rg1 isomer	C42H72O14	800.4922	B1, B2, B4	-9.88
186	9.71	915.5327	915.5387[M-H]$^-$ 783.5006[M-H-ara/xyl]$^-$ 621.4406[M-H-ara/xyl-glc]$^-$ 459.3950[M-H-ara/xyl-2glc]$^-$	PPD1-glc-glc-ara/xyl	CS-III isomer	C47H80O17	916.5396	B1, B2, B3	-0.98

187	9.75	945.5404	945.5440[M-H]⁻ 783.4906[M-H-glc]⁻ 621.4374[M-H-2glc]⁻ 459.3732[M-H-3glc]⁻	PPD1-glc-glc-glc	Rd isomer	C48H82O18	946.5501	B1, B2, B3, B4	2.01
188	9.77	1031.5434	1031.5494[M-H]⁻ 987.5551[M-H-CO2]⁻ 945.5428[M-H-CO2-Ac]⁻ 783.5040[M-H-CO2-Ac-glc]⁻ 621.4093[M-H-CO2-Ac-2glc]⁻ 459.3853[M-H-CO2-Ac-3glc]⁻	PPD1-glc-glc-glc-malonyl	Malonyl-ginsenoside Re isomer	C51H84O21	1032.5505	B1, B2, B3, B4	-0.68
189	9.78	987.5535	987.5529[M-H]⁻ 945.5419[M-H-Ac]⁻ 927.5268[M-H-Ac-H2O]⁻ 783.4873[M-H-Ac-glc]⁻ 765.4957[M-H-Ac-glc-H2O]⁻ 621.4374[M-H-Ac-2glc]⁻ 459.3875[M-H-Ac-3glc]	PPD1-3glc-acetyl	6'''-O-acetylgypenoside XVII isomer	C50H84O19	988.5607	B1, B2, B3, B4	-0.61
190	9.825	815.48178	815.4829[M+HCOO]⁻ 769.4762[M-H]⁻ 637.4333[M-H-Xyl]⁻ 475.3792[M-H-Xyl-Glc]⁻	20(S)-NG-R2	20(S)-NG-R2	C41H70O13	770.4816	B1, B2, B3, B4	-3.12
191	9.82738	769.47612	769.4793[M-H]⁻ 637.4369[M-H-Ara]⁻ 475.3803[M-H-Ara-Glc]⁻	G-F3	G-F3	C41H70O13	770.4816	B1, B2, B3, B4	-3.01
192	9.9	1239.6351	1077.5562[M-H-glc]⁻ 945.5306[M-H-glc-xyl/ara]⁻ 915.5393[M-H-2glc]⁻ 783.4931[M-H-2glc-xyl/ara]⁻ 621.4484[M-H-3glc-xyl/ara]⁻ 459.3743[M-H-4glc-xyl/ara]⁻	PPD1-4glc-xyl/ara	Ra3 isomer	C59H100O27	1240.6452	B1, B2, B3, B4	1.86
193	10.14	793.4352	793.4415[M-H]⁻ 631.3958[M-H-glc]⁻ 455.3711[M-H-glc-glcA]⁻	OA-glcA-glc	CS-Iva isomer	C42H66O14	794.4453	B1, B3	2.90
194	10.18	769.4749	769.4803[M-H]⁻ 637.4379[M-H-xyl/ara]⁻ 475.3849[M-H-xyl/ara]⁻	PPT1-glc-xyl/ara	G-F3 isomer	C41H70O13	770.4816	B1, B2, B3, B4	-1.43
195	10.2472	1209.6298	1077.5890[M-H-Xyl]⁻ 945.5514[M-H-Xyl-Ara(f)]⁻ 783.4951[M-H-Xyl-Ara(f)-Glc]⁻ 621.4350[M-H-Xyl-Ara(f)-2Glc]⁻	G-Ra2	G-Ra2	C58H98O26	1210.6346	B1, B2, B3, B4	-2.45

196	10.39	887.4989	887.5080[M+FA-H]$^-$ 841.5007[M-H]$^-$ 799.4905[M-H-Ac]$^-$ 781.4728[M-H-Ac-H2O]$^-$ 637.4221[M-H-Ac-glc]$^-$ 475.3722[M-H-Ac-2glc]$^-$	PPT1-glc-glc-acetyl	noto-Rt/isomer	C44H74O15	842.5028	B2, B4	-6.77
197	10.42	1029.563	1029.5696[M-H]$^-$ 987.5551[M-H-Ac]$^-$ 945.5430[M-H-2Ac]$^-$ 783.4938[M-H-2Ac-glc]$^-$ 621.4501[M-H-2Ac-2glc]$^-$ 459.4029[M-H-2Ac-3glc]$^-$	PPD1-glc-glc-glc-acetyl-acetyl	Diacetyl-G-Rd isomer	C52H86O20	1030.5712	B1, B2, B3, B4	0.39
198	10.4238	1239.6404	1107.5963[M-H-Xyl]$^-$ 945.5535[M-H-Xyl-Glc]$^-$ 783.4811[M-H-Xyl-2Glc]$^-$	G-Ra3	G-Ra3	C59H100O27	1240.6452	B1, B2, B3, B4	-2.40
199	10.4658	769.47477	769.4790[M-H]$^-$ 637.4347[M-H-Xyl]$^-$ 475.3819[M-H-Xyl-Glc]$^-$	20(R)-NG-R2	20(R)-NG-R2	C41H70O13	770.4816	B1, B2, B4	-1.26
200	10.4913	1107.5974	1107.6017[M-H]$^-$	G-Rb1	G-Rb1	C54H92O23	1108.6029	B1, B2, B3, B4	-2.05
201	10.552	783.49235	783.4928[M-H]$^-$ 637.4318[M-H-Rha]$^-$ 475.3794[M-H-Rha-Glc]$^-$	20(R)-G-Rg2	20(R)-G-Rg2	C42H72O13	784.4973	B1, B2, B3, B4	-3.64
202	10.552	783.49235	783.4928[M-H]$^-$ 637.4318[M-H-Rha]$^-$ 475.3794[M-H-Rha-Glc]$^-$	20(S)-G-Rg2	20(S)-G-Rg2	C42H72O13	784.4973	B1, B2, B3, B4	-3.64
203	10.62	793.4381	793.4445[M-H]$^-$ 631.4132[M-H-glc]$^-$ 613.3784[M-H-glc-H2O]$^-$ 455.3560[M-H-glc-glcA]$^-$	OA-glcA-glc	CS-Iva isomer	C42H66O14	794.4453	B1, B3	-0.76
204	10.64	913.5193	913.5207[M+FA-H]$^-$ 867.5122[M-H]$^-$ 799.4863[M-H-C4H4O]$^-$ 781.4766[M-H-C4H4O-H2O]$^-$ 637.4431[M-H-C4H4O-glc]$^-$ 619.4244[M-H-C4H4O-glc-H2O]$^-$ 475.3763[M-H-C4H4O-2glc]$^-$	PPT1-glc-glc-butenoyl	koryoginsenoside-R1/isomer	C46H76O15	868.5184	B1, B2, B3, B4	-1.84
205	10.64	683.4398	683.4405[M+FA-H]$^-$ 637.4328[M-H]$^-$ 475.3751[M-H-glc]$^-$	PPT1-glc	F1 isomer	C36H62O9	638.4394	B1, B2, B3, B4	-1.88

206	10.67	1031.5691	1031.5747[M-H]⁻ 989.5585[M-H-Ac]⁻ 971.5472[M-H-Ac-H2O]⁻ 945.5629[M-H-Ac-CO2]⁻ 783.4926[M-H-Ac-CO2-glc]⁻ 621.4489[M-H-Ac-CO2-2glc]⁻ 459.3765[M-H-Ac-CO2-3glc]⁻	PPD1-glc-glc-glc-malonyl	Malonyl-ginsenoside Re isomer	C51H84O21	1032.5505	B1	-4.56
207	10.78	827.4786	827.4932[M+FA-H]⁻ 781.4815[M-H]⁻ 635.4420[M-H-rha-glc]⁻ 473.3696[M-H-rha-glc]⁻	PPT3-glc-rha	ginsenoside Rh14 isomer	C42H70O13	782.4816	B4	-9.85
208	10.92	825.494	825.4940[M-H]⁻ 783.4792[M-H-Ac]⁻ 621.4465[M-H-Ac-glc]⁻ 459.4059[M-H-Ac-2glc]	PPD1-glc-glc-acetyl	Rs3 isomer	C44H74O14	826.5079	B1, B2	7.39
209	10.95	1029.5636	1029.5586[M-H]⁻ 987.5503[M-H-Ac]⁻ 945.5490[M-H-2Ac]⁻ 765.4753[M-H-2Ac-glc-H2O]⁻ 621.4652[M-H-2Ac-2glc]⁻ 459.3855[M-H-2Ac-3glc]⁻	PPD1-glc-glc-glc-acetyl-acetyl	Diacetyl-G-Rd isomer	C52H86O20	1030.5712	B1, B2, B3, B4	-0.19
210	10.97	829.413	829.5156[M+FA-H]⁻ 783.4889[M-H]⁻ 637.4366[M-H-rha]⁻ 475.3769[M-H-rha-glc]⁻	PPT1-rha-glc	Rg2 isomer	C42H72O13	784.4973	B1, B2, B3, B4	0.77
211	10.97	793.4395	793.4411[M-H]⁻ 631.3752[M-H-glc]⁻ 613.3723[M-H-glc-H2O]⁻ 455.3515[M-H-glc-glcA]	OA-glcA-glc	CS-Iva isomer	C42H66O14	794.4453	B1, B2, B3, B4	-2.52
212	10.99	815.4803	815.4651[M+FA-H]⁻ 769.4804[M-H]⁻ 637.4293[M-H-xyl/ara]⁻ 475.3784[M-H-xyl/ara-glc]⁻	PPT1-glc-xyl/ara	20(S)-NG-R2 isomer	C41H70O13	770.4816	B3, B4	-8.58
213	11.03	1239.6332	1077.5899[M-H-glc] 945.5191[M-H-glc-xyl/ara]⁻ 783.5035[M-H-2glc-xyl/ara]⁻ 621.4306[M-H-3glc-xyl/ara]⁻ 459.3937[M-H-4glc-xyl/ara]⁻	PPD1-glc-glc-glc-glc-xyl/ara	Ra3 isomer	C59H100O27	1240.6452	B1, B3	3.39
214	11.0408	1209.6294	1077.5893[M-H-Xyl]⁻ 945.5445[M-H-Xyl-Ara(p)]⁻ 783.4912[M-H-Xyl-Ara(p)-Glc]⁻	G-Ra1	G-Ra1	C58H98O26	1210.6346	B1, B2, B3, B4	-2.15

215	11.0588	1123.5928	1123.5928[M+HCOO]⁻ 1077.5920[M-H]⁻ 945.5500[M-H-Ara(f)]⁻ 783.4880[M-H-Ara(f)-Glc]⁻	G-Rc	G-Rc	C53H90O22	1078.5924	B1, B2, B3, B4	-6.87
216	11.1163	683.43968	683.4425[M+HCOO]⁻ 637.4366[M-H]⁻ 475.3809[M-H-Glc]⁻	20(R)-G-Rh1	20(R)-G-Rh1	C36H62O9	638.4393	B1, B2, B3, B4	-8.00
217	11.1163	683.43968	683.4425[M+HCOO]⁻ 637.4366[M-H]⁻ 475.3809[M-H-Glc]⁻	20(S)-G-Rh1	20(S)-G-Rh1	C36H62O9	638.4393	B1, B2, B3, B4	-8.00
218	11.19	845.4871	845.4671[M+FA-H]⁻ 799.4874[M-H]⁻ 637.4502[M-H-glc]⁻ 475.3769[M-H-2glc]⁻	PPT1-2glc	Rg1 isomer	C42H72O14	800.4922	B1, B2, B3, B4	-3.75
219	11.39	825.5003	825.5060[M-H]⁻ 783.4959[M-H-Ac]⁻ 621.4384[M-H-Ac-glc]⁻ 459.3862[M-H-Ac-2glc]⁻	glc-PPD1-glc-acetyl	Rs3 isomer	C44H74O14	826.5079	B1, B2, B3, B4	-0.24
220	11.43	913.518	913.5177[M+FA-H]⁻ 867.5096[M-H]⁻ 799.4952[M-H-C4H4O]⁻ 781.4744[M-H-C4H4O-H2O]⁻ 637.4505[M-H-C4H4O-glc]⁻ 619.4416[M-H-C4H4O-glc-H2O]⁻ 475.3893[M-H-C4H4O-2glc]⁻	PPT1-glc-glc-butenoyl	koryoginsenoside-R1/isomer	C46H76O15	868.5184	B2, B3, B4	1.15
221	11.49	869.4876	869.5031 825.4869 783.4580 621.4303 459.3656	glc-PPD1-glc-malonyl	β-D-Glucopyranoside, (3β,12β)-20-[[6-O-(2-carboxyacetyl)-β-D-glucopyranosyl]oxy]-12-hydroxydammar	C45H74O16	870.4977	B1, B2, B3	2.65

					-24-en-3-yl isomer				
222	11.5494	1077.5876	1077.5888[M-H]⁻ 945.5490[M-H-Ara(p)]⁻ 783.4941[M-H-Ara(p)-Glc]⁻	G-Rb2	G-Rb2	C53H90O22	1078.5924	B1, B2, B3, B4	-2.82
223	11.5494	1077.5876	1077.5888[M-H]⁻	G-Rb3	G-Rb3	C53H90O22	1078.5924	B1, B2, B3, B4	-2.82
224	11.58	825.4972	825.4998[M-H]⁻ 783.4838[M-H-Ac]⁻ 765.4795[M-H-Ac-H2O]⁻ 621.4407[M-H-Ac-glc]⁻ 603.4303[M-H-Ac-glc-H2O]⁻ 459.3765[M-H-Ac-2glc]⁻	PPD1-glc-glc-acetyl	Rs3 isomer	C44H74O14	826.5079	B1, B2, B3, B4	3.51
225	11.6	845.4915	845.4839[M+FA-H]⁻ 799.4891[M-H]⁻ 637.4364[M-H-glc]⁻ 475.3769[M-H-2glc]⁻	PPT1-2glc	Rg1 isomer	C42H72O14	800.4922	B1, B2, B3, B4	-5.88
226	11.77	841.4951	841.4975[M-H]⁻ 799.4912[M-H-Ac]⁻ 679.4430[M-H-glc]⁻ 781.4742[M-H-Ac-H2O]⁻ 637.4256[M-H-Ac-glc]⁻ 619.4249[M-H-Ac-glc-H2O]⁻ 475.3691[M-H-Ac-2glc]⁻	acetyl-PPT1-glc-glc	noto-Rt/isomer	C44H74O15	842.5028	B1, B2, B3, B4	-0.12
227	11.82	1209.6284	1077.5880[M-H-Xyl]⁻ 1047.5737[M-H-Glc]⁻ 945.5471[M-H-Xyl-Ara(p)]⁻ 783.4912[M-H-Xyl-Ara(f)-Glc]⁻ 765.4903[M-H-Xyl-Ara(f)-Glc-H2O]⁻ 621.4420[M-H-Xyl-Ara(f)-2Glc]⁻ 459.3802[M-H-Xyl-Ara(f)-3Glc]⁻	PPD1-glc-glc-glc-ara-xyl	G-Ra1 isomer	C58H98O26	1210.6346	B3, B4	-1.32
228	11.91	825.4982	825.4972[M-H] - 783.5059[M-H-Ac]⁻ 765.4744[[M-H-Ac-H2O]⁻ 621.4430[M-H-Ac-glc]⁻ 475.3766[M-H-Ac-glc-rha]⁻	acetyl-PPT1-glc-rha	β-D-Glucopyranoside, (3β,6α,12β,	C44H74O14	826.5079	B1, B2, B3, B4	2.30

					20*R*)-3,12,20-trihydroxydammar-24-en-6-yl 2-*O*-(6-deoxy-α-L-mannopyranosyl)-, 6-acetate isomer				
229	11.97	969.5422	969.5422 923.5105 783.4861 621.4352 459.3712	PPD1-3glc-acetyl	6'''-O-acetylgypenoside XVII isomer	C50H84O19	988.5607	B1, B2, B4	0.10
230	12	785.467	785.4922[M+FA-H]⁻ 739.4622[M-H]⁻ 607.4191[M-H-xyl/ara]⁻ 475.3762[M-H-2xyl/ara]⁻	PPT1-xyl/ara-xyl/ara	β-D-Xylopyranoside, (3β,6α,12β)-3,12,20-trihydroxydammar-24-en-6-yl 2-*O*-xylopyranosyl-isomer	C40H68O12	740.4711	B1, B2, B3	1.49

231	12.0736	1149.6077	1149.6140[M-H]$^-$ 1107.5995[M-H-Ac]$^-$ 945.5271[M-H-Ac-Glc]$^-$ 783.4751[M-H-Ac-2Glc]$^-$	Q-R1	Q-R1	C56H94O24	1150.6135	B1, B2, B3, B4	-1.78
232	12.08	869.4886	869.4886[M-H]$^-$ 825.5047[M-H-CO2]$^-$ 783.4911[M-H-CO2-Ac]$^-$ 765.4845[M-H-CO2-Ac-H2O]$^-$ 621.4388[M-H-CO2-Ac-glc]$^-$ 459.3842[M-H-CO2-Ac-2glc]$^-$	glc-PPD1-glc-malonyl	β-D-Glucopyranoside, (3β,12β)-20-[[6-*O*-(2-carboxyacetyl)-β-D-glucopyranosyl]oxy]-12-hydroxydammar-24-en-3-yl isomer	C45H74O16	870.4977	B1, B2, B3, B4	1.50
233	12.1	683.4369	683.4372[M+FA-H]$^-$ 637.4343[M-H]$^-$ 475.3808[M-H-glc]$^-$	PPT1-glc	F1 isomer	C36H62O9	638.4394	B1, B2, B3, B4	-4.24
234	12.14	1047.5746	1047.5763[M-H]$^-$ 915.5345[M-H-xyl/ara]$^-$ 783.4937[M-H-2xyl/ara]$^-$ 621.4351[M-H-2xyl/ara-glc]$^-$	PPD1-glc-glc-xyl/ara-xyl/ara	Notoginsenoside O isomer	C52H88O21	1048.5818	B1, B2, B3, B4	-0.57
235	12.17	1013.5601	1013.5523[M-H]$^-$ 945.5216[M-H-C4H4O]$^-$ 783.4902[M-H-C4H4O-glc]$^-$ 621.4246[M-H-C4H4O-glc-glc]$^-$ 459.3778[M-H-C4H4O-glc-glc-glc]$^-$	PPD1-glc-glc-glc-butenoyl	Quinquenoside I isomer	C52H86O19	1014.5763	B1, B3	8.26

236	12.3	829.495	829.5220[M+FA-H]⁻ 783.4936[M-H]⁻ 621.4401[M-H-glc]⁻ 475.3775[M-H-2glc]⁻	PPT1-rha-glc	Rg2 isomer	C42H72O13	784.4973	B1, B2, B3, B4	-5.23
237	12.31	825.5011	825.4991[M-H]⁻ 783.4947[M-H-Ac]⁻ 765.4786[M-H-Ac-H2O]⁻ 663.4434[M-H-glc]⁻ 621.4706[M-H-Ac-glc]⁻ 459.3755[M-H-Ac-2glc]⁻	glc-PPD1-glc-acetyl	Rs3 isomer	C44H74O14	826.5079	B1, B2, B3, B4	-1.21
238	12.36	763.4303	763.4304[M-H-xyl/ara] 631.3865[M-H-xyl/ara]⁻ 455.3466[M-H-xyl/ara-glcA]⁻	OA-glcA-xyl/ara	3-O-[R-L-arabinopyranosyl(1f2)-β-D-glucuronopyranosyl] oleanolic acid isomer	C41H64O13	764.4347	B1, B2, B3, B4	-4.45
239	12.39	1149.6082	1149.6078[M-H]⁻ 1107.5978[M-H-Ac]⁻ 1089.5882[M-H-Ac-H2O]⁻ 945.5434[M-H-Ac-glc]⁻ 783.4872[M-H-Ac-2glc]⁻ 621.4235[M-H-Ac-3glc]⁻ 459.3798[M-H-Ac-4glc]⁻	PPD1-glc-glc-glc-glc-acetyl	Q-R1 isomer	C56H94O24	1150.6135	B1, B2, B3, B4	-2.17
240	12.4	929.5482	929.5522[M-H]⁻ 767.5045[M-H-glc]⁻ 605.4526[M-H-2glc]⁻ 459.3850[M-H-2glc-rha]⁻	PPD1-rha-glc-glc	Gypenoside X isomer	C48H82O17	930.5552	B1, B2, B3, B4	-0.86
241	12.45	1119.5937	1119.6016[M-H]⁻ 1077.5889[M-H-Ac]⁻ 1059.5754[M-H-Ac-H2O]⁻ 945.5460[M-H-Ac-xyl/ara]⁻ 927.5327[M-H-Ac-xyl/ara-H2O]⁻ 915.5334[M-H-Ac-glc]⁻ 783.4920[M-H-Ac-glc-xyl/ara]⁻ 765.4832[M-H-Ac-glc-xyl/ara-H2O]⁻ 621.4377[M-H-Ac-2glc-xyl/ara]⁻ 459.3698[M-H-Ac-3glc-xyl/ara]⁻	PPD1-glc-glc-glc-xyl/ara-acetyl	Rs1 isomer	C55H92O23	1120.6029	B1, B2, B3, B4	1.25

242	12.5606	991.5504 9	991.5525[M+HCOO]‾ 945.5460[M-H]‾ 783.4973[M-H-Glc]‾ 621.4388[M-H--2Glc]‾	G-Rd	G-Rd	C48H82O18	946.5501	B1, B2, B3, B4	-3.91
243	12.66	1047.572 4	1047.5764[M-H]‾ 915.5212[M-H-xyl/ara]‾ 753.4696[M-H-xyl/ara-glc]‾ 621.4309[M-H-2xyl/ara-glc]‾ 459.1302[M-H-2xyl/ara-2glc]‾	PPD1-glc-glc-xyl/ara-xyl/ara	Notoginsenoside O isomer	C52H88O21	1048.5818	B1, B2, B3, B4	1.53
244	12.68	1077.587 4	1077.5859[M-H]‾ 945.5425[M-H-xyl/ara]‾ 783.4687[M-H-xyl/ara-glc]‾ 621.4494[M-H-xyl/ara-2glc]‾ 459.3653[M-H-xyl/ara-3glc]‾	PPD1-glc-glc-glc-xyl/ara	Rb2 isomer	C53H90O22	1078.5924	B1, B2, B3	-2.60
245	12.761	765.4774	765.4774[M-H]‾ 603.3942[M-H-Glc]‾ 441.3382[M-H-2Glc]‾	G-Rk1	G-Rk1	C42H70O12	766.4867	B1, B2, B3, B4	1.96
246	12.79	1149.605 6	1149.6084[M-H]‾ 1107.5950[M-H-Ac]‾ 1089.5862[M-H-Ac-H2O]‾ 945.5474[M-H-Ac-glc]‾ 783.4985[M-H-Ac-2glc]‾ 621.4451[M-H-Ac-3glc]‾ 459.3763[M-H-Ac-4glc]‾	glc-PPD1-glc-glc-glc-acetyl	Q-R1 isomer	C56H94O24	1150.6135	B1, B2, B3, B4	0.09
247	12.902 5	1119.597 2	1119.6023[M-H]‾ 1077.5944[M-H-C2H2O]‾ 1077.6355[M-H-C2H2O]‾ 1059.5786[M-H-Ac]‾	Rs1	Rs1	C55H92O23	1120.6029	B1, B2, B3, B4	-1.87
248	12.902 5	1119.597 2	1119.6023[M-H]‾ 1077.5944[M-H-C2H2O]‾ 1077.6355[M-H-C2H2O]‾ 1059.5786[M-H-Ac]‾	Rs2	Rs2	C55H92O23	1120.6029	B1, B2, B3, B4	-1.87
249	12.99	871.5036	871.5212[M+FA-H]‾ 825.5077[M-H]‾ 783.4969[M-H-Ac]‾ 637.4444[M-H-Ac-rha]‾ 475.3821[M-H-Ac-rha-glc]‾	PPT1-glc-rha-acetyl	β-D-Glucopyranoside, (3β,6α,12β,	C44H74O14	826.5079	B1, B2, B3, B4	-9.21

					20*R*)-3,12,20-trihydroxydammar-24-en-6-yl 2-*O*-(6-deoxy-α-L-mannopyranosyl)-, 6-acetate isomer				
250	12.99	767.496	767.4645[M-H]$^-$ 621.4372[M-H-rha]$^-$ 459.3942[M-H-rha-glc]$^-$	PPD1-glc-rha	Gynosaponin I isomer	$C_{42}H_{72}O_{12}$	768.5024	B3, B4	-1.82
251	13	1047.5722	1047.5785[M-H]$^-$ 915.5464[M-H-xyl/ara]$^-$ 783.4876[M-H-2xyl/ara]$^-$ 621.4268[M-H-2xyl/ara-glc]$^-$ 459.4064[M-H-2xyl/ara-2glc]$^-$	PPD1-glc-glc-xyl/ara-xyl/ara	Notoginsenoside O isomer	$C_{52}H_{88}O_{21}$	1048.5818	B3, B4	1.72
252	13.05	1175.6198	1175.6281[M-H]$^-$ 1107.6023[M-H-C4H4O]$^-$ 1089.5882[M-H-C4H4O-H2O]$^-$ 945.5458[M-H-C4H4O-glc]$^-$ 783.4935[M-H-C4H4O-2glc]$^-$ 765.4780[M-H-C4H4O-2glc-H2O]$^-$ 621.4361[M-H-C4H4O-3glc]$^-$ 459.3930[M-H-C4H4O-4glc]$^-$	PPD1-glc-glc-glc-glc-butenoyl	β-D-Glucopyranoside, (3β,12β)-20-[(6-*O*-β-D-glucopyranosyl-β-D-glucopyranosyl)	$C_{58}H_{96}O_{24}$	1176.6292	B1, B2, B3, B4	1.36

					oxy]-12-hydroxydammar-24-en-3-yl 2-*O*-[6-*O*-[(2*E*)-1-oxo-2-buten-1-yl]-β-D-glucopyranosyl]- isomer				
253	13.18	945.5438	945.5491[M-H]⁻ 783.4911[M-H-glc]⁻ 621.4233[M-H-2glc]⁻ 459.3912[M-H-3glc]⁻	PPD1-glc-glc-glc	Rd isomer	C48H82O18	946.5501	B1, B2, B3, B4	-1.59
254	13.19	913.5175	913.5067[M+FA-H]⁻ 867.5118[M-H]⁻ 799.4870[M-H-C4H4O]⁻ 637.4354[M-H-C4H4O-glc]⁻ 475.3766[M-H-C4H4O-2glc]⁻	PPT1-glc-glc-butenoyl	koryoginsenoside-R1/isomer	C46H76O15	868.5184	B1, B2, B4	-1.38
255	13.2	845.4883	845.3680[M+FA-H]⁻ 799.4841[M-H]⁻ 637.4318[M-H-glc]⁻ 475.3432[M-H-2glc]⁻	PPT1-2glc	Rg1 isomer	C42H72O14	800.4922	B1, B3	0.38
256	13.22	1099.6684	1099.5663[M-H]⁻ 945.5487[M-H-decanoyl]⁻ 783.4966[M-H-decanoyl-glc]⁻ 621.4190[M-H-decanoyl-2glc]⁻ 459.2828[M-H-decanoyl-3glc]⁻	PPD1-glc-glc-glc-decanoyl	β-D-Glucopyranoside, (3β,12β)-20-(β-D-glucopyranosyloxy)-12-	C58H100O19	1100.6859	B1, B2, B3, B4	8.82

					hydroxydammar-24-en-3-yl 2-*O*-[6-*O*-(1-oxodecyl)-β-D-glucopyranosyl]- (9CI)				
257	13.22	725.4481	725.4566[M+FA-H]⁻ 679.4468[M-H]⁻ 637.4486[M-H-Ac]⁻ 475.3851[M-H-Ac-glc]⁻	PPT1-glc-acetyl	β-D-Glucopyranoside, (3β,6α,12β,20S)-6-(acetyloxy)-3,12-dihydroxydammar-24-en-20-yl isomer	C38H64O10	680.4499	B1, B2, B3, B4	-6.92
258	13.32	681.4207	681.4132[M+FA-H]⁻ 635.4165[M-H]⁻ 473.3689[M-H-glc]⁻	PPT3-glc	Rh5 isomer	C36H60O9	636.4237	B1, B2, B3, B4	
259	13.42	1145.6106	1145.6186[M-H]⁻ 1077.5918[M-H-C4H4O]⁻ 1059.5801[M-H-CC4H4O-H2O]⁻ 945.5450[M-H-C4H4O-xyl/ara]⁻ 783.4872[M-H-C4H4O-xyl/ara-glc]⁻ 765.4993[M-H-C4H4O-xyl/ara-glc-H2O]⁻ 621.4356[M-H-C4H4O-xyl/ara-2glc]⁻ 459.3738[M-H-C4H4O-xyl/ara-3glc]⁻	xyl/ara-PPD1-glc-glc-glc-butenoyl	ginsenoside Ra7 isomer	C57H94O23	1146.6186	B1, B2, B3, B4	0.17

260	13.44	961.5386	961.5420[M+FA-H]$^-$ 915.5353[M-H]$^-$ 753.4954[M-H-glc]$^-$ 621.4523[M-H-glc-xyl/ara]$^-$ 459.4213[M-H-2glc-xyl/ara]$^-$	PPD1-glc-glc-xyl/ara	CS-III isomer	C48H86O16	914.5603	B1, B2, B3, B4	
261	13.45	1119.5962	1119.5975[M-H]$^-$ 1077.5926[M-H-Ac]$^-$ 1059.5864[M-H-Ac-H2O]$^-$ 945.5513[M-H-Ac-xyl/ara]$^-$ 915.5329[M-H-Ac-glc]$^-$ 783.5016[M-H-Ac-glc-xyl/ara]$^-$ 621.4504[M-H-Ac-2glc-xyl/ara]$^-$ 459.5302[M-H-Ac-3glc-xyl/ara]$^-$	PPD1-glc-glc-glc-xyl/ara-acetyl	Rs1 isomer	C55H92O23	1120.6029	B1, B2, B3, B4	-0.98
262	13.4884	969.50923	969.516[M-H]$^-$ 807.4603[M-H-Glc]$^-$ 609.3828[M-H-2Glc-2H2O]$^-$ 455.3540[M-H-2Glc-GluA-CH2]$^-$	G-Ro methyl ester	G-Ro methyl ester	C49H78O19	970.5137	B1, B2, B3, B4	-3.43
263	13.57	827.4787	827.4883[M+FA-H]$^-$ 781.4707[M-H]$^-$ 619.4312[M-H-glc]$^-$ 457.3872[M-H-2glc]$^-$	PPT2-glc-glc	G-Rh15/isomer	C42H70O13	782.4816	B1, B2, B3, B4	3.97
264	13.62	917.5386	917.5449[M-H]$^-$ 785.5032[M-H-xyl/ara]$^-$ 623.4563[M-H-xyl/ara-glc]$^-$ 461.3923[M-H-xyl/ara-2glc]$^-$	PPD5-glc-glc-xyl/ara	β-D-Glucopyranoside, (3β,12β)-12, 20-dihydroxydammaran-3-yl *O*-β-D-glucopyranosyl-	C47H82O17	918.5552	B2, B3	9.59

					(1→2)-*O*-[β-D-xylopyranosyl-(1→6)]- (9CI)				
265	13.77	827.48141	827.4802[M+HCOO]⁻ 781.4798[M-H]⁻ 619.4225[M-H-glc]⁻ 457.3717[M-H-2glc]⁻	G-Rg9	G-Rg9	$C_{42}H_{70}O_{13}$	782.4816	B1, B2, B3, B4	-7.68
266	13.78	987.5549	987.5549[M-H]⁻ 945.5439[M-H-Ac]⁻ 927.5337[M-H-Ac-H2O]⁻ 783.4908[M-H-Ac-glc]⁻ 765.4852[M-H-Ac-H2O-glc]⁻ 621.4333[M-H-Ac-2glc]⁻ 459.4046[M-H-Ac-3glc]⁻	glc-PPD1-2glc-acetyl	6'''-O-acetylgypenoside XVII isomer	$C_{50}H_{84}O_{19}$	988.5607	B1, B2, B3, B4	-2.03
267	13.78	915.5343	915.5353[M-H]⁻ 783.4908[M-H-ara/xyl]⁻ 621.4370[M-H-ara/xyl-glc]⁻ 459.3812[M-H-ara/xyl-2glc]⁻	PPD1-glc-glc-ara/xyl	CS-III isomer	$C_{47}H_{80}O_{17}$	916.5396	B1, B2, B3, B4	-2.73
268	13.81	913.5163	913.5406[M+FA-H]⁻ 867.5045[M-H]⁻ 799.4865[M-H-C4H4O]⁻ 781.4857[M-H-C4H4O-H2O]⁻ 637.4456[M-H-C4H4O-glc]⁻ 571.4121 475.3700[M-H-C4H4O-2glc]	PPT1-glc-glc-butenoyl	koryoginsenoside-R1/isomer	$C_{46}H_{76}O_{15}$	868.5184	B2, B3, B4	7.03
269	13.8946	637.43315	637.4349[M-H]⁻ 475.3828[M-H-glc]⁻	G-Rh19	G-Rh19	$C_{36}H_{62}O_9$	638.4394	B1, B2, B3, B4	-2.43
270	14.0807	807.49106	807.4943[M-H]⁻ 765.4824[M-H-Ac]⁻ 747.4726[M-H-Ac-H2O]⁻ 603.4296[M-H-Ac-glc]⁻ 441.3694[M-H-Ac-2glc]⁻	PPD2-2glc-acetyl	Rs4 isomer	$C_{44}H_{72}O_{13}$	808.4973	B1, B2, B3, B4	-1.93
271	14.096	783.49136	783.4913[M-H]⁻ 621.4361[M-H-Glc]⁻	DHDGG	DHDGG	$C_{42}H_{72}O_{13}$	784.4973	B2, B4	-2.37
272	14.17	751.4632	751.4642[M-H]⁻ 619.4247[M-H-xyl/ara]⁻ 457.3737[M-H-xyl/ara-glc]⁻	PPT2-glc-xyl/ara	NG-T5 isomer	$C_{41}H_{68}O_{12}$	752.4711	B1, B2, B3, B4	0.13

273	14.26	987.5523	987.5542[M-H]⁻ 945.5451[M-H-Ac]⁻ 927.5273[M-H-Ac-H2O]⁻ 783.4858[M-H-Ac-glc]⁻ 765.4884[M-H-Ac-glc-H2O]⁻ 621.4309[M-H-Ac-glc-glc]⁻ 459.3818[M-H-Ac-glc-glc-glc]	glc-PPD1-2glc-acetyl	6'''-O-acetylgypenoside XVII isomer	C50H84O19	988.5607	B1, B2, B3, B4	0.61
274	14.3926	811.48711	811.4896[M+HCOO]⁻ 765.4833[M-H]⁻ 619.4246[M-H-Rha]⁻ 457.3736[M-H-Rha-glc]⁻	G-Rg6	G-Rg6	C42H70O12	766.4867	B1, B2, B3, B4	-5.75
275	14.3941	765.4814	765.482[M-H]⁻ 619.4232[M-H-Rha]⁻	G-F4	G-F4	C42H70O12	766.4867	B1, B2, B3, B4	-3.27
276	14.4632	797.47064	751.4998[M-H]⁻ 797.4733[M+HCOO]⁻ 619.4275[M-H-Xyl]⁻	DHDXG	DHDXG	C42H72O11	752.5075	B1, B2, B3, B4	-0.13
277	14.4954	807.45439	645.4015[M-H-Glc]⁻ 807.4582[M-H]⁻	CS-Iva methyl ester	CS-Iva methyl ester	C43H68O14	808.4609	B1, B2, B3, B4	-1.59
278	14.5	725.4486	725.4466[M+FA-H]⁻ 679.4402[M-H]⁻ 637.4289[M-H-Ac]⁻ 475.3965[M-H-Ac-glc]⁻	PPT1-glc-acetyl	β-D-Glucopyranoside, (3β,6α,12β,20S)-6-(acetyloxy)-3,12-dihydroxydammar-24-en-20-yl isomer	C38H64O10	680.4499	B1, B2, B4	2.80
279	14.68	1059.577	1059.5753[M+FA-H]⁻ 1013.5717[M-H]⁻ 945.5444[M-H-C4H4O]⁻ 927.5337[M-H-C4H4O-H2O]⁻ 783.4901[M-H-C4H4O-glc]⁻ 765.4861[M-	PPD1-glc-glc-glc-butenoyl	Quinquenoside I isomer	C52H86O19	1014.5763	B1, B2, B3, B4	-3.16

			H-C4H4O-glc-H2O]$^-$ 621.4401[M-H-C4H4O-2glc]$^-$ 459.3767[M-H-C4H4O-3glc]$^-$						
280	14.68	975.5552	975.5552[M+FA-H]$^-$ 929.5511[M-H]$^-$ 783.4878[M-H-rha]$^-$ 621.4535[M-H-rha-glc]$^-$ 459.3782[M-H-rha-glc-glc]	rha-PPD1-glc-glc	Gypenoside X isomer	C48H82O17	930.5552	B1, B2, B3, B4	-3.98
281	14.68	811.4877	811.4762[M+FA-H]$^-$ 765.4814[M-H]$^-$ 619.4101[M-H-rha]$^-$ 457.3726[M-H-rha-glc]$^-$	G-Rg6 isomer	G-Rg6 isomer	C42H70O12	766.4867	B1, B2, B3, B4	-3.27
282	14.72	825.4591	825.4723[M+FA-H]$^-$ 779.4533[M-H]$^-$ 617.3951[M-H-glc]$^-$ 455.3551[M-H-2glc]$^-$	OA-glc-glc	Erythrosaponin B	C42H68O13	780.466	B1, B4	6.29
283	14.8346	665.42862	665.4299[M+HCOO]$^-$ 619.4241[M-H]$^-$	G-Rh4	G-Rh4	C36H60O8	620.4288	B1, B2, B3, B4	-5.00
284	14.8346	665.42862	665.4299[M+HCOO]$^-$ 619.4241[M-H]$^-$	G-Rk3	G-Rk3	C36H60O8	620.4288	B1, B2, B3, B4	-5.00
285	14.84	827.4788	827.4481 781.4740 619.4313 457.3703	PPT2-glc-glc	G-Rh15/isomer	C42H70O13	782.4816	B1, B2, B3, B4	-0.26
286	14.91	1059.5739	1059.5742[M+FA-H]$^-$ 1013.5706[M-H]$^-$ 945.5519[M-H-C4H4O]$^-$ 927.5194[M-H-C4H4O-H2O]$^-$ 783.4810[M-H-C4H4O-glc]$^-$ 765.4677 [M-H-C4H4O-glc-H2O]$^-$ 621.4466[M-H-C4H4O-2glc]$^-$	PPD1-glc-glc-glc-butenoyl	Quinquenoside I isomer	C52H86O19	1014.5763	B1, B2, B3, B4	-2.07
287	14.92	945.5409	945.5442[M-H]$^-$ 783.4678[M-H-glc]$^-$ 621.4510[M-H-2glc]$^-$ 459.3463[M-H-3glc]$^-$	PPD1-glc-glc-glc	Rd isomer	C48H82O18	946.5501	B1, B2, B4	1.48
288	14.98	783.49097	783.4913[M-H]$^-$ 621.4361[M-H-Glc]$^-$	G-F2	G-F2	C42H72O13	784.4973	B1, B2, B3, B4	-1.88
289	15.34	827.4784	827.4749[M+FA-H]$^-$ 781.4764[M-H]$^-$ 619.4216[M-H-glc]$^-$ 457.3858[M-H-2glc]$^-$	PPT2-glc-glc	G-Rh15/isomer	C42H70O13	782.4816	B3, B4	-3.33

290	15.39	1013.5701	1013.5723[M-H]$^-$ 945.5463[M-H-C4H4O]$^-$ 927.5273[M-H-C4H4O-H2O]$^-$ 783.4935[M-H-C4H4O-glc]$^-$ 765.4950[M-H-C4H4O-glc-H2O]$^-$ 621.4445[M-H-C4H4O-2glc]$^-$ 459.3787[M-H-C4H4O-3glc]$^-$	PPD1-glc-glc-glc-butenoyl	Quinquenoside I isomer	C52H86O19	1014.5763	B1, B2, B3, B4	-1.58
291	15.6	1029.5647	1029.5679[M-H]$^-$ 987.5458[M-H-Ac]$^-$ 945.5502[M-H-2Ac]$^-$ 783.5046[M-H-2Ac-glc]$^-$ 621.4294[M-H-2Ac-2glc]$^-$ 459.3784[M-H-2Ac-3glc]$^-$	PPD1-glc-glc-glc-acetyl-acetyl	Diacetyl-G-Rd isomer	C52H86O20	1030.5712	B1, B2, B3, B4	-1.26
292	15.7	681.4203	681.4254 635.4122 473.3730	PPT3-glc	Rh5 isomer	C36H60O9	636.4237	B1, B2	
293	15.76	1231.6845	1107.5937[M-H-octenoyl]$^-$ 1089.5847[M-H-octenoyl-H2O]$^-$ 945.5622[M-H-octenoyl-glc]$^-$ 783.4521[M-H-octenoyl-2glc]$^-$ 621.4537[M-H-octenoyl-3glc]$^-$ 459.4149[M-H-octenoyl-4glc]$^-$	PPD1-4glc-octenoyl	quinquenoside II isomer	C62H104O24	1232.6918	B1, B2, B3, B4	-0.41
294	15.79	931.5279	931.5271[M+FA-H]$^-$ 885.5209[M-H]$^-$ 753.4783[M-H-xyl/ara]$^-$ 621.4376[M-H-2xyl/ara]$^-$ 459.3730[M-H-2xyl/ara-glc]$^-$	PPD1-2xyl/ara-glc	Compound Ra1 isomer	C46H78O16	886.529	B3, B4	0.34
295	15.88	1059.5734	1059.5758[M+FA-H]$^-$ 1013.5717[M-H]$^-$ 945.5350[M-H-C4H4O]$^-$ 927.5350[M-H-C4H4O-H2O]$^-$ 927.5546 783.4812[M-H-C4H4O-glc]$^-$ 621.4560 [M-H-C4H4O-2glc]$^-$	PPD1-glc-glc-glc-butenoyl	Quinquenoside I isomer	C52H86O19	1014.5763	B1, B2, B3, B4	-3.16

296	15.9	827.477	827.4912[M+FA-H]‾ 781.4725[M-H]‾ 619.4240[M-H-glc]‾ 457.3695[M-H-2glc]	PPT2-2glc	G-Rh15/isomer	C42H70O13	782.4816	B1, B3, B4	1.66
297	16.16	851.5094	851.4918[M-H]‾ 783.4875[M-H-C4H4O]‾ 621.4525[M-H-C4H4O-glc]‾ 459.3966[M-H-C4H4O-2glc]‾	PPD1-glc-glc-butenoyl	β-D-Glucopyranoside, (3β,12β)-3-(β-D-glucopyranosyloxy)-12-hydroxydammar-24-en-20-yl, 6-(2*E*)-2-butenoate isomer	C46H76O14	852.5235	B1, B2, B4	7.40
298	16.18	783.4925	783.4947[M-H]‾ 621.4389[M-H-Glc]‾ 459.3840[M-H-2Glc]‾	20(R)-G-Rg3	20(R)-G-Rg3	C42H72O13	784.4973	B1, B2, B3, B4	-3.83
299	16.18	783.4925	783.4947[M-H]‾ 621.4389[M-H-Glc]‾ 459.3840[M-H-2Glc]‾	20(S)-G-Rg3	20(S)-G-Rg3	C42H72O13	784.4973	B1, B2, B3, B4	-3.83
300	16.31	681.4227	681.4258[M+FA-H]‾ 635.4307[M-H]‾ 473.3700[M-H-glc]‾	PPT3-glc	Rh5 isomer	C36H60O9	636.4237	B3	
301	16.46	829.4954	829.5001[M+FA-H]‾ 783.4931[M-H]‾ 621.4475[M-H-glc]‾ 459.3833[M-H-2glc]‾	PPD1-glc-glc	F2 isomer	C42H72O13	784.4973	B1, B2, B3, B4	-4.59
302	16.97	459.3845	459.3845[M+H]+ 441.3741[M+H-H2O]+	DEDT	DEDT	C30H50O3	458.376	B1, B2, B3, B4	-1.52

303	17.0143	475.37871	475.3786[M-H]⁻ 391.2836[M-H-C6H12]⁻	20(R)-PPT1	20(R)-PPT1	$C_{30}H_{52}O_4$	476.3866	B1, B2, B3, B4	0.19
304	17.0143	475.37871	475.3786[M-H]⁻ 391.2836[M-H-C6H12]⁻	20(S)-PPT1	20(S)-PPT1	$C_{30}H_{52}O_4$	476.3866	B1, B2, B3, B4	0.19
305	17.18	799.4847	799.4891[M+FA-H]⁻ 753.4784[M-H]⁻ 621.4433[M-H-xyl/ara]⁻ 459.3814[M-H-xyl/ara-glc]⁻	PPD1-xyl/ara-glc	G-C-Y isomer	$C_{41}H_{70}O_{12}$	754.4867	B1, B2, B3, B4	0.66
306	17.35	827.4803	827.4759[M+FA-H]⁻ 781.4773[M-H]⁻ 619.4345[M-H-glc]⁻ 457.3735[M-H-2glc]⁻	PPT2-glc-glc	G-Rh15/isomer	$C_{42}H_{70}O_{13}$	782.4816	B1, B2, B3, B4	-4.48
307	17.55	799.4844	799.4859[M+FA-H]⁻ 753.4824[M-H]⁻ 621.4417[M-H-xyl/ara]⁻ 459.3995[M-H-xyl/ara-glc]⁻	PPD1-glc-xyl/ara	G-C-Y isomer	$C_{41}H_{70}O_{12}$	754.4867	B1, B2, B3, B4	-4.65
308	17.7	871.5045	871.5041[M+FA-H]⁻ 825.4979[M-H]⁻ 783.4866[M-H-Ac]⁻ 621.4428[M-H-Ac-glc]⁻ 459.3854[M-H-Ac-glc-glc]	PPD1-glc-glc-acetyl	Rs3 isomer	$C_{44}H_{74}O_{14}$	826.5079	B1, B2, B4	2.67
309	18.33	871.5073	871.4739[M+HCOO]⁻ 825.5006[M-H]⁻ 783.4949[M-H-C2H2O] 765.4761[M-H-AC]⁻ 621.4482[M-H-Glc]⁻ 459.3823[M-H-2Glc]⁻	20(R)-G-Rs3	20(R)-G-Rs3	$C_{44}H_{74}O_{14}$	826.5079	B1, B2, B3, B4	-0.61
310	18.33	871.5073	871.4739[M+HCOO]⁻ 825.5006[M-H]⁻ 783.4949[M-H-C2H2O] 765.4761[M-H-AC]⁻ 621.4482[M-H-Glc]⁻ 459.3823[M-H-2Glc]⁻	20(S)-G-Rs3	20(S)-G-Rs3	$C_{44}H_{74}O_{14}$	826.5079	B1, B2, B3, B4	-0.61
311	18.4408	807.4955	807.4955[M-H]⁻ 609.3828[M-H-Glc-2H2O]⁻	Rs4	Rs4	$C_{44}H_{72}O_{13}$	808.4973	B1, B2, B3, B4	-7.43
312	18.4408	807.45483	807.4572[M-H]⁻ 609.3828[M-H-Glc-2H2O]⁻ 537.3592 455.3544[M-H-GlcA-6'-O-Me-Glc]	ZB-RI-6‘ methyl ester	ZB-RI-6‘ methyl ester	$C_{43}H_{68}O_{14}$	808.4609	B1, B2, B3, B4	-2.14

313	18.45	797.4658	797.4840[M+FA-H]¯ 751.4653[M-H]¯ 619.4250[M-H-xyl/ara]¯ 457.3681[M-H-xyl/ara-glc]¯	PPT2-glc-xyl/ara	noto-T5 isomer	C41H68O12	752.4711	B1, B2, B3, B4	-2.66
314	19.06	663.4096	663.4111[M+FA-H]¯ 617.40474[M-H]¯ 455.3506[M-H-glc]¯	OA-glc	Oleanolic acid-28-O-β-D-glucopyranoside /isomer	C36H58O8	618.4132	B1, B2, B3, B4	1.13
315	19.07	665.4161	665.4419[M+FA-H]¯ 619.4148[M-H]¯ 457.3730[M-H-glc]¯	PPT2-glc	G-Rh4 isomer	C36H60O8	620.4288	B1, B2, B3, B4	10.00
316	19.9	667.4459	621.4426[M-H]¯ 459.3852[M-H-Glc]¯	G-C-K	G-C-K	C36H62O8	622.4445	B1, B2, B3, B4	-9.49
317	19.9316	765.48153	765.4820[M-H]¯ 603.4280[M-H-Glc]¯ 441.3389[M-H-2Glc]¯	G-Rg5	G-Rg5	C42H70O12	766.4867	B1, B2, B3, B4	-3.44
318	20.19	851.5149	851.5131[M-H]¯ 783.4955[M-H-C4H4O]¯ 765.4755[M-H-C4H4O-H2O]¯ 621.4417[M-H-C4H4O-glc]¯ 459.3909[M-H-C4H4O-2glc]¯	PPD1-glc-glc-butenoyl	β-D-Glucopyranoside, (3β,12β)-3-(β-D-glucopyranosyloxy)-12-hydroxydammar-24-en-20-yl, 6-(2*E*)-2-butenoate	C46H76O14	852.5235	B1, B2, B4	0.94

319	21.0154	621.43715	621.4382[M-H]⁻ 459.3831[M-H-Glc]⁻	20(S)-G-Rh2	20(S)-G-Rh2	C36H62O8	622.4445	B1, B2, B3, B4	-0.72
320	21.0154	621.43715	621.4382[M-H]⁻ 459.3831[M-H-Glc]⁻	20(S)-G-Rh2	20(S)-G-Rh2	C36H62O8	622.4445	B1, B2, B3, B4	-0.72
321	21.29	665.4263	665.4260[M+FA-H]⁻ 619.4224[M-H]⁻ 457.3760[M-H-glc]⁻	PPT2-glc	G-Rh4 isomer	C36H60O8	620.4288	B1, B2, B3, B4	-2.26
322	21.68	459.3819	459.3819[M+H]+ 441.3741[M+H-H20]+	DDT	DDT	C30H50O3	458.376	B1, B2, B3, B4	4.14
323	21.9083	807.49047	807.4898[M-H]⁻ 765.4821[M-H-Ac] 747.4694[M-H-Ac-H2O]⁻ 603.4212[M-H-Ac-glc]⁻ 441.3721[M-H-Ac-2glc]	PPD2-2glc-acetyl	Rs4 isomer	C44H72O13	808.4973	B1, B2, B3, B4	-1.20
324	23.7187	649.43368	649.4332[M+HCOO]⁻ 603.4311[M-H]⁻	G-Rh3	G-Rh3	C36H60O7	604.4339	B1, B2, B3, B4	-8.29
325	23.7187	649.43368	649.4332[M+HCOO]⁻ 603.4311[M-H]⁻	G-Rk2	G-Rk2	C36H60O7	604.4339	B1, B2, B3, B4	-8.29
326	25.5428	505.38907	505.3847[M+HCOO]⁻ 459.3830[M-H]⁻	20(S)-PPD1	20(S)-PPD1	C30H52O3	460.3916	B1, B2, B3, B4	1.74
327	25.5428	505.38907	505.3847[M+HCOO]⁻ 459.3830[M-H]⁻	20(S)-PPD1	20(S)-PPD1	C30H52O3	460.3916	B1, B2, B3, B4	1.74

Table S3

The contents of 41 constituents in the 4 groups of *P. ginseng* samples (n = 3, mg/g).

No.	Compounds	B1	B2	B3	B4
1	20-glu-Rf	0.545	0.382	0.558	0.595
2	NG-R_1	0.483	0.403	0.206	0.213
3	G-Rg_1	3.539	1.986	6.366	0.627
4	G-Re	7.187	5.973	4.952	1.091
5	G-Ro	4.411	4.460	3.506	0.565
6	PG-F_{11}	Nd	Nd	Nd	Nd
7	PG-RT_5	Nd	Nd	Nd	Nd
8	CS-IVa	2.774	4.505	0.984	0.892
9	*S*-Rf	1.211	0.847	1.525	0.456
10	G-Ra_3	2.852	1.093	0.126	0.621
11	G-Ra_1	0.208	0.090	7.300	1.113
12	G-Ra_2	0.670	0.307	0.839	0.088
13	*S*-NG-R_2	0.555	0.421	1.103	0.177
14	*R*-NG-R_2	0.017	0.016	0.464	0.044
15	*S*-Rg_2	1.465	0.999	1.098	0.744
16	*R*-Rg_2	0.004	0.003	0.002	0.115
17	G-Rb_1	3.197	2.082	2.128	0.482
18	*S*-Rh_1	0.130	0.079	0.069	0.085
19	*R*-Rh_1	0.041	0.029	0.008	0.005
20	G-Rc	2.394	1.687	1.650	0.489
21	G-Rb_2	2.945	2.183	0.639	0.618
22	G-Rb_3	5.177	2.512	1.057	1.004
23	Q-R_1	0.845	0.642	0.249	0.522
24	G-Rs_1	0.138	0.116	0.107	0.174
25	G-Rg_9	Nd	Nd	0.019	0.012
26	*S*-Rg_3	0.011	0.010	0.009	0.014
27	*R*-Rg_3	0.015	0.017	0.017	0.010
28	G-F_2	0.027	0.016	0.050	0.021
29	Ro ME	0.009	0.005	Nd	0.007
30	CS-IVa ME	0.002	0.002	0.001	Nd
31	*S*-PPT	0.008	0.002	0.014	0.003
32	*R*-PPT	0.004	0.001	0.015	0.001
33	*S*-Rs_3	0.001	0.001	Nd	0.002
34	*R*-Rs_3	0.001	0.001	Nd	0.002
35	G-C-K	0.016	0.018	0.007	0.002
36	G-Rk_1	0.008	0.008	0.003	0.005
37	G-Rg_5	0.066	0.078	0.056	0.040
38	G-Rh_2	0.013	0.002	0.007	0.004

39	G-Rs_4	0.025	0.034	0.059	0.008
40	*S*-PPD	0.009	0.003	Nd	0.005
41	*R*-PPD	0.002	0.001	0.032	0.008

Nd: not detected

Table S4

The semi-quantitative data of 408 ginsenosides in 4 batches of *P. ginseng* samples.

Rt	Found At Mass	Identification	Name	B1	B2	B3	B4
1.18	861.4858	PG-glc-glc	Panajaponol A/isomer	1.9E+05	8.2E+04	2.0E+05	9.9E+04
1.25	863.5005	PPT4-glc-glc	20-S-Rf3/ isomer	2.0E+04		2.8E+04	2.6E+04
1.32	695.4369	PG-glc-acetyl	acetyl-PG-RT5A	7.2E+03	4.1E+04	2.9E+04	4.5E+03
1.37	1003.5493	PPT1-glc-glc-glc-acetyl	6-acetyl ginsenoside-Rg3/isomer	2.0E+04	1.3E+04	2.9E+04	2.4E+04
1.39	975.5436	PPD4/PPT5-glc-glc-xyl/ara-acetyl	H-acetyl-NG-R1A	5.8E+03	6.1E+03	7.4E+03	
1.52	861.4849	PG-glc-glc	Panajaponol A/isomer		6.6E+03	9.4E+03	6.5E+03
1.59	829.4844	PPT1-rha-glc	Rg2 isomer	3.6E+03		1.0E+04	
1.6	885.4866	glc-PPT1-glc-malonyl	mRg1 isomer	2.1E+04	1.6E+04	6.1E+04	
1.62	843.503	PPD4/PPT5-glc-glc-acetyl	H-acetyl-Rg1A	6.2E+03		2.0E+04	
1.62	841.4954	acetyl-PPT1-glc-glc	noto-Rt/isomer	3.7E+04	2.8E+04	1.5E+06	4.6E+04
1.62	827.4795	PPT2-glc-glc	G-Rh15/isomer	1.6E+04	1.5E+04	4.6E+04	2.7E+04
1.63	987.5533	PPT1-glc-glc-rha-acetyl	Pseudoginsenoside Rs1/isomer	2.7E+04	3.0E+04	3.2E+04	3.7E+04
1.66	1003.5456	PPT1-glc-glc-glc-acetyl	6-acetyl ginsenoside-Rg3/isomer		1.3E+04		1.7E+04
1.68	695.4434	PG-glc-acetyl	acetyl-PG-RT5B		9.3E+03	4.2E+03	8.2E+03
1.79	1031.544	PPT1-glc-glc-rha-malonyl	mRd isomer	3.3E+04		3.6E+05	4.7E+04
1.87	887.4938	PPD4/PPT5-glc-glc-malonyl	H-malonyl-NG-R1	8.1E+03		3.1E+05	
1.87	861.4859	PG-glc-glc	Panajaponol A/isomer	2.7E+03			4.1E+03
1.89	885.4879	glc-PPT1-glc-malonyl	mRg1 isomer	1.3E+06	1.6E+04	1.8E+06	3.1E+04
1.93	1123.5858	PPT1-4glc	Notoginsenoside Fh7 isomer	3.0E+04		2.0E+04	4.1E+04
1.95	799.4848	PPT1-2glc	Rg1 isomer	6.0E+03	1.6E+04	3.9E+03	2.7E+04
1.96	945.5399	PPT1-glc-glc-rha	G-Re isomer		1.0E+04		

1.98	829.4873	PPT1-rha-glc	Rg2 isomer	8.4E+03			8.8E+03
2.09	987.5524	glc-PPT1-glc-rha-acetyl	Pseudoginsenoside Rs1/isomer	1.2E+06	5.2E+05	4.0E+05	6.5E+05
2.1	1031.5443	PPT1-glc-glc-rha-malonyl	mRd isomer	1.6E+06	5.5E+05	4.4E+04	2.6E+04
2.15	861.4839	PG-glc-glc	Panajaponol A/isomer	6.2E+03		4.7E+03	1.4E+03
2.21	843.5069	PPD4/PPT5-glc-glc-acetyl	H-acetyl-Rg1B	5.5E+03		9.1E+03	
2.21	885.4873	glc-PPT1-glc-malonyl	mRg1 isomer	5.1E+04	7.1E+05	3.8E+04	1.3E+06
2.21	841.498	acetyl-PPT1-glc-glc	noto-Rt/isomer	9.3E+05	6.1E+05	1.6E+05	7.9E+05
2.25	987.5548	glc-PPT1-glc-rha-acetyl	Pseudoginsenoside Rs1/isomer	8.0E+04	5.0E+04	7.8E+04	2.7E+04
2.34	827.4815	PPT2-glc-glc	G-Rh15/isomer	2.6E+04	1.2E+04	6.6E+04	1.4E+04
2.36	799.4806	PPT1-2glc	Rg1 isomer	4.2E+03	4.6E+03	1.2E+04	2.1E+03
2.38	945.5413	PPT1-glc-glc-rha	G-Re isomer	1.8E+04	1.0E+04	1.1E+04	9.1E+03
2.41	927.4953	PPT1-glc-glc-malonyl-acetyl	malonyl-acetyl-Rg1C	2.1E+04	1.3E+04	1.3E+04	1.1E+04
2.71	861.4856	PG-glc-glc	Panajaponol A/isomer	2.3E+04	1.4E+04	2.0E+04	1.8E+04
2.72	799.4849	PPT1-2glc	Rg1 isomer	1.2E+03	4.6E+03	1.9E+03	2.6E+03
2.74	885.4877	glc-PPT1-glc-malonyl	mRg1 isomer	1.1E+04	5.1E+03	2.3E+04	3.0E+04
2.77	841.4953	PPT1-glc-glc-acetyl	noto-Rt/isomer	8.2E+03	4.9E+03	1.7E+04	2.9E+04
2.79	945.5448	PPT1-glc-glc-rha	G-Re isomer	6.0E+03		3.8E+03	
2.82	863.4849	PPT4-glc-glc	20-S-Rf3/ isomer	1.5E+03	3.1E+03	3.0E+03	2.2E+03
2.83	987.5541	PPT1-glc-glc-rha-acetyl	20-S-Rf3/ isomer	2.1E+04	8.1E+03	2.2E+04	4.8E+03
2.84	1093.5771	PPT1-glc-glc-glc-xyl/ara	20S-sanchirhinosides A6 isomer	5.9E+03	5.2E+03	7.4E+03	2.0E+03
2.87	1031.5437	PPT1-glc-glc-rha-malonyl	mRd isomer	3.1E+04	4.7E+03	2.6E+04	3.0E+03
2.92	1123.5888	PPT1-glc-glc-glc-glc	Notoginsenoside Fh7 isomer	1.9E+04			2.9E+04
3	861.4824	PG-glc-glc	Panajaponol A/isomer	2.5E+03	3.3E+04	3.2E+03	2.6E+03
3.07	885.4847	glc-PPT1-glc-malonyl	mRg1 isomer		5.1E+03	2.2E+03	7.1E+03

3.16	811.484	PPT1-glc-ara/xyl-acetyl	β-D-Glucopyranoside, (3β,6α, 12β,20*R*)-3,12,20-trihydroxydammar-24-en-6-yl 2-*O*-β-D-xylopyranosyl-, 6-acetate isomer			1.3E+03	
3.2	961.5397	PPT1-glc-glc-glc	Re1 isomer	9.8E+03	7.4E+03	2.5E+04	1.3E+04
3.3	947.52	PG-glc-glc-xyl/ara	Vinaginsenoside R6 isomer	1.4E+03	1.9E+03	2.9E+03	
3.375229	815.4816011	PG(母核)-2glc 或如图	Panajaponol A/isomer	4.3E+04	1.1E+04	1.6E+05	5.2E+04
3.38	883.5058	PG-glc-glc--butenoyl	butenoyl-PG-RT5-G			1.0E+04	
3.4	863.4889	PPT4-glc-glc	20-S-Rf3/ isomer	4.3E+03	6.8E+03	2.1E+03	1.0E+04
3.43	1063.5697	PG-glc-rha-xyl/ara-xyl/ara	PG-F11-X2	4.6E+03		5.5E+03	2.8E+03
3.67	1123.5924	PPT1-4glc	Notoginsenoside Fh7 isomer	1.8E+04	1.4E+04	6.9E+03	1.4E+04
3.67	861.4837	PG-glc-glc	Panajaponol A/isomer	2.8E+03	2.1E+03		2.6E+03
3.68	961.5382552	G-Re3	G-Re3	5.2E+05	3.0E+05	3.0E+05	5.9E+05
3.69	811.4863	PPT1-glc-ara/xyl-acetyl	β-D-Glucopyranoside, (3β,6α, 12β,20*R*)-3,12,20-trihydroxydammar-24-en-6-yl 2-*O*-β-D-xylopyranosyl-, 6-acetate isomer			1.9E+03	
3.79	1009.5535	PPD4/PPT5-glc-glc-glc	Re-mo isomer	2.8E+04	2.6E+04	2.2E+04	
3.82	831.4767	PG-glc-ara/xyl	M-R2/isomer	1.3E+04	8.9E+03	8.8E+03	1.8E+03
3.88	855.4702	PPT1-glc-xyl/ara-malonyl	malonyl-NG-R2A	1.3E+03		1.8E+03	
4.15	787.4832	PPT4-glc-xyl/ara	3β,12β,20,25-tetrahydroxydammarane-6-O-β-D-xylopyranosyl-(1→2)-β-D-glucopyranoside isomer	8.3E+02			

4.45	653.4267	PG-glc	PG-F11 isomer	1.0E+03	9.5E+02	8.0E+02	1.1E+03
4.48	961.538	PPT1-glc-glc-glc	Re1 isomer	1.9E+03	1.0E+06	2.8E+03	1.4E+06
4.52	855.476	PPT1-glc-xyl/ara-malonyl	malonyl-NG-R2B			1.4E+03	
4.56	811.4856	PPT1-glc-ara/xyl-acetyl	β-D-Glucopyranoside, (3β,6α, 12β,20*R*)-3,12,20-trihydroxydammar-24-en-6-yl 2-*O*-β-D-xylopyranosyl-, 6-acetate isomer		7.8E+03	5.2E+03	7.1E+02
4.61	1007.54364	20-glu-G-Rf	20-glu-G-Rf	3.5E+05	5.8E+05	2.5E+03	8.4E+05
4.635203	931.528357	G-Re4	G-Re4	1.0E+05	6.7E+04	1.3E+05	9.1E+04
4.66	845.4894	PG-glc-rha	PG-F11 isomer	8.4E+03	6.5E+03	2.7E+03	7.0E+03
4.68	831.475	PG-glc-ara/xyl	M-R2/isomer	7.2E+04	3.8E+03	2.1E+05	8.8E+03
4.731942	1007.54462	G-Re2	G-Re2	9.4E+05	4.5E+05	1.6E+06	7.2E+05
4.75	999.5567	PPT1-glc-glc-xyl/ara-butenoyl	butenoyl-Re1	8.1E+03		9.3E+04	5.4E+04
4.82	1077.5826	ara/xyl-rha-PPT1-glc-glc	Floralginsenoside M isomer	6.5E+04	3.7E+04	4.5E+04	3.1E+04
4.85	1093.5789	PPT1-glc-glc-glc-xyl/ara	20S-sanchirhinosides A6 isomer	8.3E+04	8.7E+04	3.5E+04	1.1E+04
4.9	883.5063	PPT1-glc-glc-acetyl-acetyl	6',6"-di-O-acetyl Rg1 isomer	6.3E+03	5.2E+03	8.2E+03	1.1E+04
4.91	861.4853	PG-glc-glc	Panajaponol A/isomer	2.1E+03	3.2E+03	2.7E+03	4.6E+03
4.91	811.4826	PPT1-glc-ara/xyl-acetyl	β-D-Glucopyranoside, (3β,6α, 12β,20R)-3,12,20-trihydroxydammar-24-en-6-yl 2-O-β-D-xylopyranosyl-, 6-acetate isomer		4.7E+03		5.6E+03
5.01	845.4846	PG-glc-rha	PG-F11 isomer		2.8E+03	7.0E+03	
5.03	925.5862	PPT1-glc-glc-octanoyl	octanoyl-Rg1			1.5E+04	

5.03	701.4383	PPT4-rha	α-L-Mannopyranoside, (3β,12β)-3,12,20,24,25-pentahydroxydammaran-6-yl 6-deoxy- isomer			3.2E+03	
5.03945	653.4263151	PG-glc	PG-RT5 isomer	4.2E+03	4.3E+03	2.3E+04	4.7E+03
5.08	931.5247	PPT1-glc-glc-xyl/ara	NG-R1 isomer	2.0E+05	1.3E+05	1.5E+05	1.9E+05
5.19	1107.5973	glc-glc-PPT1-glc-rha	Yesanchinoside E/isomer		5.3E+04		
5.192836	931.5284102	NG-R1	NG-R1	1.9E+06	1.3E+06	2.1E+06	1.2E+05
5.22	1077.586	ara/xyl-PPT1-glc-glc-rha	Floralginsenoside M isomer	1.9E+05	9.0E+04	8.6E+04	5.5E+04
5.29	887.4939	PPD4/PPT5-glc-glc-malonyl	H-malonyl-F2B			8.6E+03	9.7E+03
5.3	799.4853	PG-F11 isomer	PG-F11 isomer	4.1E+04	2.2E+04	3.3E+04	1.7E+04
5.34	983.5583	PPT1-glc-glc-malonyl-hexanoyl	malonyl-hexanoyl-Rg1	1.4E+04		2.0E+04	
5.34	961.5397054	G-Re1	G-Re1	5.5E+05	4.7E+05	5.4E+05	1.1E+06
5.35	1279.5996	PPD1-glc-glc-glc-glc-malonyl-malonyl	D-malonyl-Rb1	4.1E+05	3.4E+05	6.4E+05	1.9E+05
5.35	1003.5427	PPT1-glc-glc-glc-acetyl	6-acetyl ginsenoside-Rg3/isomer	3.7E+04	2.0E+04	2.9E+03	1.3E+04
5.36	1193.5915	PPT1-glc-glc-glc-rha-malonyl	malonyl-Re-Ga	3.0E+04		5.0E+04	
5.38	1009.5498	PPD4/PPT5-glc-glc-glc	Re-mo isomer	3.5E+04	3.0E+04	3.9E+04	
5.44	885.482	glc-PPT1-glc-malonyl	mRg1 isomer	2.7E+05	1.3E+05	3.7E+05	3.5E+05
5.46	861.4864	PG-glc-glc	Panajaponol A/isomer	3.0E+04	2.5E+04	3.2E+04	3.8E+04
5.47	1191.6141	PPD1-glc-glc-glc-glc-2acetyl	Diacetyl-G-Rb1 isomer	6.0E+04	2.0E+04	4.6E+04	
5.47	841.497	glc-PPT1-glc-acetyl	noto-Rt/isomer	8.5E+05	5.2E+04	8.5E+04	6.1E+05
5.5	817.4849	PPT4-glc-glc	R1-Hy isomer			2.2E+03	
5.54	931.5264	PPT1-glc-glc-ara/xyl	Re4 isomer/NG-R1 isomer	3.3E+04	2.7E+04	3.2E+04	7.1E+03
5.6	1107.5946	glc-glc-PPT1-glc-rha	Yesanchinoside E/isomer				5.0E+06
5.62	961.5394	PPT1-glc-glc-glc	Re1 isomer	1.3E+05	1.4E+05	1.2E+05	4.4E+05

5.66	1249.5866	PPD1-glc-glc-glc-xyl/ara-malontl-malonyl	D-malonyl-Rc	1.1E+05		1.3E+05	
5.66	653.4207	PG-glc	PG-F11 isomer		3.2E+03		
5.74	1047.5349	PPT1-glc-glc-glc-malonyl	malonyl-Re2-C	3.2E+04	2.0E+04		1.1E+04
5.76	883.5036	PPT1-glc-glc-acetyl-acetyl	6',6"-di-O-acetyl Rg1 isomer	3.5E+03			
5.773957	845.4929834	G-Rg1	G-Rg1	1.2E+07	9.9E+06	1.5E+07	1.2E+07
5.811302	991.5497354	G-Re	G-Re	1.3E+07	1.2E+07	1.7E+07	1.2E+07
5.86	995.6385	PPT1-2glc-decadienoyl	decadienoyl-Rg1		9.8E+04		1.1E+05
5.923654	811.4851388	PPT1-glc-xyl/ara-acetyl	β-D-Glucopyranoside, (3β,6α, 12β,20*R*)-3,12,20-trihydroxydammar-24-en-6-yl 2-*O*-β-D-xylopyranosyl-, 6-acetate isomer	5.1E+04	2.0E+05	2.4E+05	4.2E+04
5.98	1249.5851	PPD1-glc-glc-glc-xyl/ara-malony-malonyl	D-malonyl-Rc	1.3E+05	4.5E+04	1.3E+04	1.7E+04
6.03	971.5583	PPD1-glc-glc-rha-acetyl	acetyl-C-K-GR	1.0E+05		2.7E+04	
6.03	841.4946	PPT1-glc-glc-acetyl	noto-Rt/isomer	6.3E+04	4.9E+04	8.3E+04	4.5E+04
6.07	827.5129	PPD4/PPT5-glc-rha-acetyl	H-acetyl-Rg2			6.7E+03	
6.07	931.5273	PPT1-glc-glc-ara/xyl	Re4 isomer/NG-R1 isomer	4.7E+03			6.9E+03
6.12	885.4868	glc-PPT1-glc-malonyl	mRg1 isomer	1.3E+04		1.4E+04	1.1E+04
6.13	825.496	PPD1-glc-glc-acetyl	Rs3 isomer	7.6E+04	2.0E+04	3.5E+04	3.2E+04
6.14	1031.535	PPT1-glc-glc-rha-malonyl	mRd isomer	2.3E+05			4.0E+04
6.15	831.4744	PG-glc-ara/xyl	M-R2/isomer		2.2E+04		
6.16	969.5454	PPD1-3glc-acetyl	6'''-O-acetylgypenoside XVII isomer	7.5E+05	1.7E+05	2.7E+05	1.5E+05
6.18	1123.5938	PPT1-4glc	Notoginsenoside Fh7 isomer		3.3E+04		

6.19	825.4991	acetyl-PPT1-glc-rha	β-D-Glucopyranoside, (3β,6α, 12β,20*R*)-3,12,20-trihydroxydammar-24-en-6-yl 2-*O*-(6-deoxy-α-L-mannopyranosyl)-, 6-acetate isomer	2.1E+04	1.9E+04	2.0E+04	3.9E+04
6.2	799.4847	PPT1-2glc	Rg1 isomer	1.6E+04	4.7E+03		
6.26	1029.5618	PPD1-glc-glc-glc-acetyl-acetyl	Diacetyl-G-Rd isomer	3.4E+05			
6.29	679.4419	PPT1-glc-acetyl	20R-ginsenoside Rh1 6‘-acetate isomer	4.2E+04	4.3E+04	1.3E+05	7.5E+04
6.29326	955.492	G-Ro	G-Ro	1.3E+07	1.2E+08	1.3E+07	1.2E+07
6.32	665.3858	PPT2-glc	G-Rh4 isomer	5.9E+03	4.0E+03	2.7E+03	3.6E+03
6.37	845.4893	PG-F11 isomer	PG-F11 isomer	1.6E+04	4.7E+03		
6.43	931.5232	PPT1-glc-glc-ara/xyl	Re4 isomer/NG-R1 isomer	8.1E+03	4.8E+03	9.5E+03	9.0E+03
6.46	855.4767	PPT1-glc-xyl/ara-malonhyl	malonhyl-NG-R2	9.0E+03	5.2E+03	1.6E+04	1.8E+03
6.5	831.4723	PG-glc-ara/xyl	M-R2/isomer	1.6E+03	3.1E+03	3.0E+03	
6.51	815.478	PG-glc-glc	Panajaponol A/isomer	1.3E+03	2.3E+03		3.4E+03
6.51	811.4834	PPT1-glc-ara/xyl-acetyl	β-D-Glucopyranoside, (3β,6α, 12β,20*R*)-3,12,20-trihydroxydammar-24-en-6-yl 2-*O*-β-D-xylopyranosyl-, 6-acetate isomer	2.2E+04	1.1E+04	2.6E+04	
6.52	1093.5808	PPT1-glc-glc-glc-xyl/ara	20S-sanchirhinosides A6 isomer	2.3E+04	1.6E+04		9.1E+03
6.54	1003.5454	PPT1-glc-glc-glc-acetyl	6-acetyl ginsenoside-Rg3/isomer	4.5E+03	4.5E+03		8.2E+03
6.54	769.4724	PPT1-glc-xyl/ara	F3 isomer	5.9E+03	3.2E+03	7.6E+03	

6.64	1291.6627	PPD1-glc-glc-glc-glc-hexanoyl-malonyl	hexanoyl-malonyl-Rb1	3.3E+04	2.6E+04	4.8E+04	2.2E+04
6.64	947.522	PPT1-glc-xyl/ara-xyl/ara	chikusetsusaponin LM2 isomer	5.6E+03	7.8E+03	9.4E+03	
6.72	961.5359	PPT1-glc-rha-xyl/ara	β-D-Glucopyranoside, (3β,6α, 12β)-3,12-dihydroxy-20-(β-D-xylopyranosyloxy)dammar-24-en-6-yl 2-*O*-(6-deoxy-α-L-mannopyranosyl)- isomer	2.8E+03		9.2E+03	
6.73	1149.6066	PPD1-glc-glc-glc-glc-acetyl	Q-R1 isomer	7.4E+06	5.4E+06	8.7E+06	3.4E+06
6.742854	925.4817601	CS-IV	CS-IV	1.2E+05	1.7E+05	1.9E+05	1.8E+05
6.75	1281.6478	PPD1-glc-glc-glc-glc-acetyl-xyl/ara	O-acetyl-G-Ra3 isomer		9.4E+02	5.4E+04	1.8E+03
6.76	815.4796	PPT1-glc-xyl/ara	20(S)-NG-R2 isomer	1.5E+04	5.2E+03	1.9E+04	
6.77	679.4418	PPT1-glc-acetyl	20R-ginsenoside Rh1 6‘-acetate isomer	6.3E+03	3.3E+03	1.5E+04	5.5E+03
6.79	1193.5966	PPD1-glc-glc-glc-glc-malonyl	Ginsenoside Ma-Rb1 isomer	8.3E+06	4.1E+06	7.2E+06	1.6E+06
6.81	1153.6172	PPD1-glc-glc-glc-glc	Rb1 isomer	1.7E+05	6.5E+04	2.7E+05	
6.82	973.5329	PPT1-glc-glc-xyl/ara-acetyl	acetyl-NG-R1C		4.4E+03		
6.95	825.5009	PPT1-glc-rha-acetyl	β-D-Glucopyranoside, (3β,6α, 12β,20*R*)-3,12,20-trihydroxydammar-24-en-6-yl 2-*O*-(6-deoxy-α-L-mannopyranosyl)-, 6-acetate isomer	1.0E+04	5.3E+03		7.2E+03
6.98	1149.6074	glc-PPD1-glc-glc-glc-acetyl	Q-R1 isomer	2.8E+06	1.9E+06	5.1E+06	1.9E+06
7.04	769.473	PPT1-glc-xyl/ara	F3 isomer	5.2E+03	1.5E+04	4.6E+04	7.6E+03

7.080361	1119.595938	R-s2isomer	R-s2 isomer	2.5E+06	3.6E+06	6.8E+06	4.2E+06
7.13	1077.5817	xyl/ara-PPD1-glc-glc-glc	Rb2 isomer	2.5E+05	9.3E+04	2.3E+05	6.9E+04
7.134982	793.4396269	CS-Iva	CS-Iva		7.2E+05	7.1E+05	9.2E+05
7.17	915.5306	PPT1-glc-rha-xyl/ara	β-D-Glucopyranoside, (3β,6α, 12β)-3,12-dihydroxy-20-(β-D-xylopyranosyloxy)dammar-24-en-6-yl 2-*O*-(6-deoxy-α-L-mannopyranosyl)- isomer	3.0E+04	1.6E+04	1.3E+04	1.5E+04
7.18	1163.5858	PPD1-glc-glc-glc-xyl/ara-malonyl	m-floral-Rc4 isomer	1.4E+06	1.1E+05	1.5E+06	
7.24	771.4855	PPD4/PPT5-glc-xyl/ara	β-D-Glucopyranoside, (3β,6α, 12β)-3,12,25-trihydroxydammaran-6-yl 2-*O*-β-D-xylopyranosyl- isomer			4.1E+03	9.6E+03
7.25	1007.5434	PPT1-glc-glc-glc	20-glu-G-Rf isomer	2.2E+04	1.2E+04	1.7E+04	3.0E+04
7.26	815.4806	PPT1-glc-xyl/ara	20(S)-NG-R2 isomer	7.0E+04	4.3E+04	1.1E+05	2.9E+04
7.27	679.4408	PPT1-glc-acetyl	20R-ginsenoside Rh1 6‘-acetate isomer	3.2E+04	1.5E+04	7.4E+03	1.2E+04
7.31	1261.645	PPD1-glc-glc-glc-xyl/ara-hexanoyl-malonyl	hexanoyl-malonyl-Rc	2.7E+04	1.6E+04	1.8E+04	1.3E+04
7.36	1121.6043	PPD5-glc-glc-glc-xyl/ara-acetyl	H-acetyl-Rc	1.0E+06	5.4E+06	2.1E+05	
7.36	841.4969	glc-PPT1-glc-acetyl	noto-Rt/isomer	9.8E+03	2.4E+04	7.2E+03	3.6E+04
7.41	1119.5978	PPD1-glc-glc-glc-xyl/ara-acetyl	Rs1 isomer	2.5E+06	2.9E+06	3.4E+06	2.2E+06
7.48	1163.589	PPD1-glc-glc-glc-xyl/ara-malonyl	Malonyl-ginsenosideRc isomer	1.4E+06	1.1E+05	4.2E+06	
7.49	1059.5786	PPD1-glc-glc-glc-butenoyl	Quinquenoside I isomer	8.0E+04	5.3E+04	7.1E+04	3.1E+04

7.52	1149.6075	Q-R1isomer	Q-R1 isomer	5.8E+04	1.0E+04	7.6E+04	1.7E+04
7.54	1077.5881	xyl/ara-PPD1-glc-glc-glc	Rb2 isomer	7.4E+04	1.9E+04	2.3E+05	1.4E+05
7.59	825.4986	PPD1-glc-glc-acetyl	Rs3 isomer	4.5E+03		3.8E+03	
7.65	931.5248	PPT1-glc-glc-ara/xyl	Re4 isomer/NG-R1 isomer	1.1E+04	5.4E+03	7.5E+03	5.4E+03
7.69	829.4938	PPT1-rha-glc	Rg2 isomer		2.7E+03	4.0E+03	3.6E+03
7.71	961.5355	PPT1-glc-glc-glc	Re1 isomer	1.1E+05	7.2E+04	6.7E+04	6.2E+04
7.74	1193.5962	PPD1-glc-glc-glc-glc-malonyl	Ginsenoside Ma-Rb1 isomer	4.2E+04	3.6E+04	8.6E+04	
7.74	987.5467	glc-PPD1-2glc-acetyl	6'''-O-acetylgypenoside XVII isomer	7.2E+03	7.5E+03	4.8E+03	3.8E+03
7.76	679.4408	PPT1-glc-acetyl	20R-ginsenoside Rh1 6‘-acetate isomer	3.1E+03	1.8E+03	1.3E+03	1.5E+03
7.798233	815.4789784	PG(母核)-2glc 或如图	Panajaponol A/isomer	7.0E+04	2.7E+04	4.3E+04	3.0E+04
7.92	807.491	PPD2-glc-glc-acetyl	20Z-ginsenoside-Rs4 isomer	5.3E+03	2.2E+03	3.7E+03	
7.97	1119.5946	PPD1-glc-glc-glc-xyl/ara-acetyl	Rs1 isomer	6.2E+05	3.3E+05	7.1E+05	3.5E+05
7.97	1059.5706	PPD1-glc-glc-glc-butenoyl	Quinquenoside I isomer		1.5E+04	3.0E+04	
8.05	799.4838	PPT1-2glc	Rg1 isomer				1.6E+03
8.08	1193.5905	PPT1-glc-glc-glc-rha-malonyl	malonyl-Re-Gb	1.0E+04	3.6E+04		1.8E+04
8.09	987.5542	PPT1-glc-glc-rha-acetyl	Pseudoginsenoside Rs1/isomer	1.6E+06	6.2E+04	2.6E+06	6.1E+04
8.16	1043.606	PPD1-glc-glc-glc-hexanoyl	hexanoyl-Rd	8.0E+04	5.5E+04	6.2E+04	3.5E+04
8.19	807.493	PPD2-glc-glc-acetyl	20Z-ginsenoside-Rs4 isomer	5.3E+03	3.2E+04		3.9E+03
8.2	825.4978	PPD1-glc-glc-acetyl	Rs3 isomer	3.2E+03			2.9E+03
8.21	1031.546	PPD1-glc-glc-glc-malonyl	Malonyl-ginsenoside Re isomer	5.8E+06	3.7E+06	4.6E+06	2.8E+06
8.25	841.4976	glc-PPT1-glc-acetyl	noto-Rt/isomer	4.4E+03	3.5E+03	3.7E+03	9.1E+03
8.26	1191.6112	PPD1-glc-glc-glc-glc-acetyl-acetyl	Diacetyl-G-Rb1 isomer	2.0E+04	3.7E+04	2.1E+04	2.5E+04
8.28	863.4994	PPT4-glc-glc	20-S-Rf3/ isomer	1.2E+03	2.1E+03	1.9E+03	1.7E+03

8.31	999.5543	PPT1-glc-glc-xyl/ara-butenoyl	butenoyl-NG-R1		4.7E+03		
8.31	945.5443	PPD1-glc-glc-glc	Rd isomer	7.2E+04	5.3E+04	3.8E+04	4.3E+04
8.34	829.4948	PPT1-rha-glc	Rg2 isomer	5.0E+03	4.5E+03	1.8E+04	9.6E+03
8.4	987.5528	glc-PPD1-2glc-acetyl	6'''-O-acetylgypenoside XVII isomer	2.2E+06	2.3E+06	1.5E+06	1.3E+06
8.43	1089.5827	PPD1-glc-glc-xyl/ara-xyl/ara-acetyl	acetyl-CS-III-G	3.1E+04	1.9E+04	2.0E+04	1.3E+04
8.48	1043.606	PPD1-glc-glc-glc-hexanoyl	hexanoyl-Rd	2.2E+04	1.3E+04	3.3E+04	1.4E+04
8.52	945.5401	PPD1-glc-glc-glc	Rd isomer	6.5E+04	2.6E+04	2.1E+04	2.2E+04
8.53	929.5466	PPD1-glc-glc-rha	Gynosaponin III isomer	1.1E+04		8.0E+03	5.9E+03
8.6	1031.546	PPD1-glc-glc-glc-malonyl	Malonyl-ginsenoside Re isomer	2.4E+06	9.3E+05	1.4E+06	6.0E+05
8.63	1119.5967	PPD1-glc-glc-glc-xyl/ara-acetyl	Rs2 isomer		2.5E+04	2.2E+04	
8.69	783.4893	PPD1-glc-glc	20(S)-G-Rg3 isomer	9.7E+03	3.1E+03	3.1E+03	
8.74	971.5768	OA-decadienoyl-2glc-acetyl	decadienoyl-acetyl-CS-2G	3.4E+05	1.5E+04	2.7E+04	1.4E+04
8.74	799.4816	PPT1-2glc	Rg1 isomer	3.8E+03	2.4E+03	1.8E+03	7.5E+03
8.75	961.5357	PPT1-3glc	Re1 isomer	3.6E+04	2.6E+04	2.7E+04	6.3E+04
8.77	831.4737	PG-glc-ara/xyl	M-R2/isomer	3.0E+04	2.5E+04	1.8E+04	4.0E+03
8.79	987.5528	glc-PPD1-2glc-acetyl	6'''-O-acetylgypenoside XVII isomer	2.0E+05	1.4E+05	2.1E+05	1.2E+05
8.81	945.5391	PPD1-glc-glc-glc	Rd isomer	4.1E+04	8.1E+03	1.1E+04	7.9E+03
8.9	977.5219	PPT1-glc-glc-xyl/ara	Re4 isomer/NG-R1 isomer	4.6E+04	4.4E+04	3.9E+03	1.4E+03
8.98	913.5198	PPT1-glc-glc-butenoyl	koryoginsenoside-R1/isomer	1.0E+05	2.1E+05	6.2E+04	8.6E+04
9.06	1031.548	PPD1-glc-glc-glc-malonyl	Malonyl-ginsenoside Re isomer	2.9E+05	2.6E+04	1.2E+05	5.5E+04
9.06	987.553	PPD1-glc-glc-glc-acetyl	6'''-O-acetylgypenoside XVII isomer	2.2E+05	3.8E+04	7.9E+04	3.3E+04
9.07	929.4474	PPT1-glc-glc-acetyl-acetyl	6',6"-di-O-acetyl Rg1 isomer	1.7E+04	6.1E+03	4.1E+03	7.4E+03

9.07504	1239.635	NG-R4	NG-R4	8.4E+02	8.0E+02	7.6E+03	2.6E+04
9.089704	845.4925986	20(S)-G-Rf-1a	20(S)-G-Rf-1a	6.6E+06	3.6E+06	8.8E+06	5.8E+06
9.090255	799.4868567	20(R)-G-Rf	20(R)-G-Rf	4.1E+06	2.7E+06	5.4E+06	2.8E+06
9.090255	799.4868567	20(S)-G-Rf	20(S)-G-Rf	4.1E+06	2.7E+06	5.4E+06	2.8E+06
9.14	897.562	PPT1-glc-glc-hexanoyl	hexanoyl-Rg1	1.5E+04	1.3E+04	3.4E+04	1.0E+04
9.15	913.4741	PPT1-glc-glc-butenoyl	koryoginsenoside-R1/isomer	8.8E+03	1.4E+04	9.9E+03	9.8E+03
9.38	823.4842	PPT2-glc-glc-acetyl	acetyl-Rg9A	2.6E+03	1.6E+03	3.3E+03	
9.4	987.553	glc-PPD1-2glc-acetyl	6'''-O-acetylgypenoside XVII isomer	3.7E+04	1.7E+04	1.6E+04	1.2E+04
9.4	929.54	PPD1-glc-glc-rha	Gynosaponin III isomer		3.7E+03		1.7E+04
9.43	931.5298	PPT1-glc-glc-ara/xyl	Re4 isomer/NG-R1 isomer	1.8E+03	1.9E+03	3.3E+03	2.0E+03
9.52	1269.65	PPD1-glc-glc-glc-glc-glc	Ra0 isomer	3.5E+03	3.3E+03	1.8E+03	4.2E+03
9.59	769.4746	PPT1-glc-xyl/ara	G-F3 isomer	4.1E+03	3.0E+03	4.3E+03	
9.66	947.5235	PPT1-glc-xyl/ara-xyl/ara	chikusetsusaponin LM2 isomer			1.8E+03	6.2E+03
9.67	845.4901	PPT1-glc-glc	Rg1 isomer	4.8E+03	2.8E+03		8.9E+03
9.68	1203.6863	PPD1-glc-glc-glc-xyl/ara-octanoyl	octanoyl-Rb2	6.5E+02		2.9E+03	
9.71	915.5327	PPD1-glc-glc-ara/xyl	CS-III isomer	4.6E+03	9.5E+02	5.4E+03	
9.75	1023.6231	PPD1-glc-glc-decanoyl-malonyl	decanoyl-malonyl-F2	1.0E+04		6.7E+03	2.6E+03
9.75	945.5404	PPD1-glc-glc-glc	Rd isomer	1.0E+04	4.0E+03	5.6E+03	3.5E+03
9.77	1031.5434	PPD1-glc-glc-glc-malonyl	Malonyl-ginsenoside Re isomer	1.7E+05	9.5E+03	8.9E+04	3.4E+04
9.78	987.5535	PPD1-3glc-acetyl	6'''-O-acetylgypenoside XVII isomer	6.0E+04	1.9E+04	4.3E+04	1.5E+04
9.81	867.5455	PPT1-glc-xyl/ara-hexanoyl	hexanoyl-F3	9.6E+03	9.1E+03	1.2E+04	5.1E+03
9.825	815.4817778	20(S)-NG-R2	20(S)-NG-R2	3.9E+06	2.5E+06	5.7E+06	5.1E+05
9.827384	769.4761157	G-F3	G-F3	1.6E+06	1.4E+06	2.8E+06	2.9E+05

9.9	1239.6351	PPD1-4glc-xyl/ara	Ra3 isomer	5.7E+02	9.7E+02	6.7E+02	2.9E+03
10.08	943.5259	PPT2-glc-glc-glc	Rg9-G	1.9E+04	1.4E+04	1.6E+04	1.4E+04
10.09	1073.5519	PPD1-glc-glc-glc-malonyl-acetyl	malonyl-acetyl-Rd	1.5E+04	6.4E+03	5.0E+03	7.2E+03
10.14	793.4352	OA-glcA-glc	CS-Iva isomer	3.3E+03		2.9E+03	
10.18	769.4749	PPT1-glc-xyl/ara	G-F3 isomer	1.4E+04	9.0E+03	1.3E+04	5.1E+03
10.24716	1209.629758	G-Ra2	G-Ra2	1.1E+03	1.2E+03	2.9E+04	7.8E+04
10.39	887.4989	PPT1-glc-glc-acetyl	noto-Rt/isomer		7.2E+03		3.7E+03
10.42	1029.563	PPD1-glc-glc-glc-acetyl-acetyl	Diacetyl-G-Rd isomer	1.5E+05	5.0E+04	3.8E+02	3.6E+04
10.42379	1239.640373	G-Ra3	G-Ra3	9.2E+02	6.5E+02	1.5E+04	4.2E+04
10.4658	769.4747725	20(R)-NG-R2	20(R)-NG-R2	4.4E+44	4.5E+03		4.3E+03
10.4913	1107.597366	G-Rb1	G-Rb1	1.0E+07	8.7E+06	1.1E+07	8.1E+06
10.55198	783.4923541	20(R)-G-Rg2	20(R)-G-Rg2	1.3E+06	7.4E+05	7.3E+05	9.4E+05
10.55198	783.4923541	20(S)-G-Rg2	20(S)-G-Rg2	1.3E+06	7.4E+05	7.3E+05	9.4E+05
10.56	833.5049	PPT1-glc-decadienoyl	decadienoyl-Rh1		1.9E+04		2.6E+04
10.58	851.5228	PPT1-rha-glc-butenoyl	butenoyl-Rg2	1.7E+04	9.5E+03		
10.62	793.4381	OA-glcA-glc	CS-Iva isomer	6.1E+04		4.4E+05	
10.64	913.5193	PPT1-glc-glc-butenoyl	koryoginsenoside-R1/isomer	2.7E+04	3.4E+04	1.2E+04	2.4E+04
10.64	683.4398	PPT1-glc	F1 isomer	5.2E+04	7.1E+03	1.5E+05	1.5E+05
10.67	1031.5691	PPD1-glc-glc-glc-malonyl	Malonyl-ginsenoside Re isomer	3.7E+04			
10.78	827.4786	PPT3-glc-rha	ginsenoside Rh14 isomer				1.7E+03
10.83	867.4659	PPT2-glc-glc-malonyl	malonyl-Rg9A	1.3E+03	1.1E+03	3.6E+03	1.0E+04
10.89	971.5536	PPD1-glc-glc-rha-acetyl	acetyl-F2-G	7.2E+03	3.8E+03	2.8E+04	2.6E+03
10.92	825.494	PPD1-glc-glc-acetyl	Rs3 isomer	2.1E+03	7.9E+02		
10.95	1029.5636	PPD1-glc-glc-glc-acetyl-acetyl	Diacetyl-G-Rd isomer	2.0E+04	3.5E+04	6.9E+03	4.2E+04
10.97	829.413	PPT1-rha-glc	Rg2 isomer	1.2E+04	1.9E+04	1.2E+04	1.6E+04
10.97	793.4395	OA-glcA-glc	CS-Iva isomer	3.4E+05	3.3E+05	7.9E+04	4.6E+05

10.99	823.4839	PPT2-glc-glc-acetyl	acetyl-Rg9B	5.4E+03	1.2E+03	7.6E+03	1.5E+03
10.99	815.4803	PPT1-glc-xyl/ara	20(S)-NG-R2 isomer			2.2E+03	2.9E+03
11.03	1239.6332	PPD1-glc-glc-glc-glc-xyl/ara	Ra3 isomer	1.4E+04		3.3E+03	
11.04081	1209.629401	G-Ra1	G-Ra1	8.2E+02	5.9E+02	4.9E+04	8.2E+04
11.05879	1123.592801	G-Rc	G-Rc	9.3E+06	5.0E+05	6.9E+06	4.7E+06
11.11627	683.4396838	20(R)-G-Rh1	20(R)-G-Rh1	5.2E+03	7.1E+03	6.5E+03	7.8E+03
11.11627	683.4396838	20(S)-G-Rh1	20(S)-G-Rh1	5.2E+03	2.2E+04	6.5E+03	7.8E+03
11.19	845.4871	PPT1-2glc	Rg1 isomer	6.5E+03	4.4E+03	3.9E+03	4.5E+03
11.24	1071.637	PPD1-glc-glc-glc-octanoyl	octanoyl-Rd	7.7E+03		8.0E+03	1.9E+03
11.39	825.5003	glc-PPD1-glc-acetyl	Rs3 isomer	1.9E+04	1.3E+04	3.8E+04	6.1E+03
11.43	913.518	PPT1-glc-glc-butenoyl	koryoginsenoside-R1/isomer		9.1E+03	3.7E+03	4.8E+03
11.49	869.4876	glc-PPD1-glc-malonyl	β-D-Glucopyranoside, (3β,12β)-20-[[6-O-(2-carboxyacetyl)-β-D-glucopyranosyl]oxy]-12-hydroxydammar-24-en-3-yl isomer	1.8E+03	2.4E+03	1.1E+04	
11.5	857.4916	PPT1-glc-xyl/ara-acetyl	acetyl-F3	1.4E+03			
11.54939	1077.58764	G-Rb2	G-Rb2	8.7E+06	6.7E+06	7.3E+06	5.4E+06
11.54939	1077.58764	G-Rb3	G-Rb3	8.7E+06	6.7E+06	7.3E+06	5.4E+06
11.58	825.4972	PPD1-glc-glc-acetyl	Rs3 isomer	1.9E+04	1.3E+04	3.7E+03	6.1E+03
11.6	845.4915	PPT1-2glc	Rg1 isomer	2.3E+03	2.6E+03	3.9E+03	3.0E+03
11.65	941.5449	PPT1-glc-glc-hexenoyl	hexenoyl-Rg1	3.6E+03	2.7E+03	3.0E+03	
11.77	841.4951	acetyl-PPT1-glc-glc	noto-Rt/isomer	2.6E+04	1.9E+04	2.0E+04	5.7E+04
11.82	1209.6284	PPD1-glc-glc-glc-ara-xyl	G-Ra1 isomer			6.0E+02	1.0E+04
11.84	895.5051	PPD1-butanoyl-glc-glc-acetyl	butanoyl-acetyl-F2	4.5E+03	2.2E+03	3.0E+03	1.1E+03

11.91	825.4982	acetyl-PPT1-glc-rha	β-D-Glucopyranoside, (3β,6α, 12β,20*R*)-3,12,20-trihydroxydammar-24-en-6-yl 2-*O*-(6-deoxy-α-L-mannopyranosyl)-, 6-acetate isomer	1.0E+05	2.5E+03	5.0E+03	4.4E+04
11.92	937.5157	PPD1-glc-glc-butenoyl-malonyl	butenoyl-malonyl-F2	2.2E+03	1.9E+03		
11.97	969.5422	PPD1-3glc-acetyl	6'''-O-acetylgypenoside XVII isomer	8.5E+03	4.8E+03		3.7E+03
12	785.467	PPT1-xyl/ara-xyl/ara	β-D-Xylopyranoside, (3β,6α,12β)-3,12,20-trihydroxydammar-24-en-6-yl 2-*O*-xylopyranosyl-isomer	9.8E+03	8.0E+03	1.3E+04	
12.07364	1149.607746	Q-R1	Q-R1	1.9E+06	1.3E+06	1.3E+06	1.7E+06
12.08	869.4886	glc-PPD1-glc-malonyl	β-D-Glucopyranoside, (3β,12β)-20-[[6-*O*-(2-carboxyacetyl)-β-D-glucopyranosyl]oxy]-12-hydroxydammar-24-en-3-yl isomer	5.1E+04	3.6E+03	2.3E+04	2.4E+04
12.1	683.4369	PPT1-glc	F1 isomer	2.5E+04	9.3E+03	1.4E+04	4.2E+04
12.14	1047.5746	PPD1-glc-glc-xyl/ara-xyl/ara	Notoginsenoside O isomer	1.6E+05	7.8E+04	3.5E+04	1.9E+04
12.17	1013.5601	PPD1-glc-glc-glc-butenoyl	Quinquenoside I isomer	5.4E+03		3.0E+03	
12.3	829.495	PPT1-rha-glc	Rg2 isomer	4.2E+03	3.3E+03	3.9E+03	5.1E+03
12.31	825.5011	glc-PPD1-glc-acetyl	Rs3 isomer	2.0E+04	1.5E+04	1.6E+04	6.9E+03

12.36	763.4303	OA-glcA-xyl/ara	3-O-[R-L-arabinopyranosyl(1f2)-β-D-glucuronopyranosyl] oleanolic acid isomer	1.8E+04	2.6E+04	7.7E+03	2.5E+04
12.39	1149.6082	PPD1-glc-glc-glc-glc-acetyl	Q-R1 isomer	5.9E+03	1.3E+05	8.4E+04	1.0E+05
12.4	929.5482	PPD1-rha-glc-glc	Gypenoside X isomer	4.0E+04	2.1E+04	3.3E+04	1.6E+04
12.45	1119.5937	PPD1-glc-glc-glc-xyl/ara-acetyl	Rs1 isomer	5.4E+05	3.6E+05	3.5E+03	6.2E+05
12.56063	991.5504876	G-Rd	G-Rd	7.2E+06	3.8E+06	4.2E+06	3.5E+06
12.58	857.4912	PPT1-glc-xyl/ara-acetyl	acetyl-NG-R2		8.8E+03		
12.65	955.5617	PPT1-glc-glc-heptenoyl	heptenoyl-Rg1	1.3E+04	8.7E+03	6.2E+03	1.6E+04
12.66	1047.5724	PPD1-glc-glc-xyl/ara-xyl/ara	Notoginsenoside O isomer	1.6E+04	1.1E+04	1.1E+04	6.4E+03
12.68	1077.5874	PPD1-glc-glc-glc-xyl/ara	Rb2 isomer	8.3E+03	3.1E+03	8.5E+03	
12.71	989.5319	PPT3-glc-glc-rha	Rh5-GR	6.1E+03	3.4E+03	5.1E+03	3.7E+03
12.76098	765.4774	G-Rk1	G-Rk1	7.7E+03	5.4E+03	3.6E+03	6.3E+03
12.79	1149.6056	glc-PPD1-glc-glc-glc-acetyl	Q-R1 isomer	7.5E+04	3.3E+04	4.5E+04	4.1E+04
12.90251	1119.597199	Rs1	Rs1	1.5E+06	1.2E+06	6.9E+05	5.4E+05
12.90251	1119.597199	Rs2	Rs2	1.5E+06	1.2E+06	6.9E+05	5.4E+05
12.91	1167.605	PPD1-glc-glc-glc-glcA	Rd-GA	1.5E+05	7.4E+04	4.5E+04	4.6E+04
12.99	871.5036	PPT1-glc-rha-acetyl	β-D-Glucopyranoside, (3β,6α,12β,20*R*)-3,12,20-trihydroxydammar-24-en-6-yl 2-*O*-(6-deoxy-α-L-mannopyranosyl)-, 6-acetate isomer	2.9E+03	3.0E+03	1.5E+03	4.0E+03
12.99	767.496	PPD1-glc-rha	Gynosaponin I isomer			1.6E+03	2.3E+03
13	1047.5722	PPD1-glc-glc-xyl/ara-xyl/ara	Notoginsenoside O isomer			1.2E+04	3.0E+04

13.05	1175.6198	PPD1-glc-glc-glc-glc-butenoyl	β-D-Glucopyranoside, (3β,12β)-20-[(6-*O*-β-D-glucopyranosyl-β-D-glucopyranosyl)oxy]-12-hydroxydammar-24-en-3-yl 2-*O*-[6-*O*-[(2*E*)-1-oxo-2-buten-1-yl]-β-D-glucopyranosyl]- isomer	4.7E+05	1.3E+06	1.7E+05	2.2E+05
13.08	1273.7025	PPD1-glc-glc-glc-glc-butenoyl-hexanoyl	butenoyl-hexanoyl-Rb1	1.1E+04	1.2E+03	9.2E+02	
13.08	799.4836	PPT1-xyl/ara-rha	PPTXR1	2.5E+03	2.4E+03	3.1E+03	
13.18	945.5438	PPD1-glc-glc-glc	Rd isomer	1.1E+05	1.9E+05	2.5E+05	1.5E+05
13.19	913.5175	PPT1-glc-glc-butenoyl	koryoginsenoside-R1/isomer	2.0E+04	3.8E+04		2.2E+04
13.2	845.4883	PPT1-2glc	Rg1 isomer	1.7E+03		3.1E+03	
13.22	1099.6684	PPD1-glc-glc-glc-decanoyl	β-D-Glucopyranoside, (3β,12β)-20-(β-D-glucopyranosyloxy)-12-hydroxydammar-24-en-3-yl 2-*O*-[6-*O*-(1-oxodecyl)-β-D-glucopyranosyl]- (9CI)	2.7E+03	2.1E+03	2.5E+03	3.1E+03
13.22	725.4481	PPT1-glc-acetyl	β-D-Glucopyranoside, (3β,6α,12β,20S)-6-(acetyloxy)-3,12-dihydroxydammar-24-en-20-yl isomer	4.4E+03	4.0E+03	4.9E+03	1.2E+04
13.32	681.4207	PPT3-glc	Rh5 isomer	1.9E+03	1.1E+03	7.2E+02	2.9E+03
13.36	985.5362	PPT2-glc-glc-glc-acetyl	acetyl-Rg9-G	4.5E+03	3.3E+03	1.9E+03	2.8E+03
13.42	1145.6106	xyl/ara-PPD1-glc-glc-glc-butenoyl	ginsenoside Ra7 isomer	1.2E+05	4.0E+05	7.9E+04	4.6E+04
13.44	961.5386	PPD1-glc-glc-xyl/ara	CS-III isomer	8.9E+04	2.2E+04	2.7E+04	1.5E+04

13.45	1119.5962	PPD1-glc-glc-glc-xyl/ara-acetyl	Rs1 isomer	9.7E+04	9.5E+04	2.4E+04	3.7E+04
13.48842	969.5092286	G-Ro methyl ester	G-Ro methyl ester	7.8E+04	9.8E+04	4.5E+03	5.4E+03
13.57	827.4787	PPT2-glc-glc	G-Rh15/isomer	3.9E+03	3.5E+03	2.6E+03	1.1E+04
13.61	981.5753	PPT1-glc-glc-decenedioyl	decenedioyl-Rg1	1.4E+01	7.7E+03	3.1E+03	
13.61	937.5889	PPT1-glc-glc-nonenoyl	nonenoyl-Rg1	2.4E+03	2.7E+03	3.0E+03	2.5E+03
13.62	917.5386	PPD5-glc-glc-xyl/ara	β-D-Glucopyranoside, (3β,12β)-12,20-dihydroxydammaran-3-yl *O*-β-D-glucopyranosyl-(1→2)-*O*-[β-D-xylopyranosyl-(1→6)]-(9CI)		8.2E+03	9.7E+03	
13.64	969.5818	PPT1-glc-glc-octenoyl	octenoyl-Rg1	9.1E+04	9.8E+04	1.1E+04	9.5E+03
13.77	827.4814115	G-Rg9	G-Rg9	4.2E+03	2.6E+03	5.7E+03	1.6E+04
13.78	987.5549	glc-PPD1-2glc-acetyl	6'''-O-acetylgypenoside XVII isomer	1.1E+06	5.4E+05	1.9E+05	5.4E+05
13.78	915.5343	PPD1-glc-glc-ara/xyl	CS-III isomer	8.2E+04	5.7E+04	9.5E+04	6.0E+04
13.81	913.5163	PPT1-glc-glc-butenoyl	koryoginsenoside-R1/isomer		3.1E+03	1.8E+03	1.4E+03
13.89462	637.4331512	G-Rh19	G-Rh19	4.0E+03	1.6E+03	2.0E+03	3.6E+03
13.97	955.5625	PPT1-glc-glc-heptenoyl	heptenoyl-Rg1	3.7E+03	2.5E+03		3.0E+03
14.08065	807.4910553	PPD2-2glc-acetyl	Rs4 isomer	6.9E+03	1.5E+04	5.0E+03	3.0E+04
14.09597	783.4913558	DHDGG	DHDGG		2.7E+03		3.4E+03
14.17	751.4632	PPT2-glc-xyl/ara	NG-T5 isomer	3.3E+03	3.2E+03	2.2E+03	2.8E+03
14.23	1085.629	PPD1-glc-glc-glc-acetyl-hexanoyl	acetyl-hexanoyl-Rd	9.3E+03			
14.26	987.5523	glc-PPD1-2glc-acetyl	6'''-O-acetylgypenoside XVII isomer	2.0E+05	3.5E+04	3.0E+04	5.9E+04
14.31	1101.6188	PPT1-glc-glc-xyl/ara-octenoyl	octenoyl-Ng-R1	4.2E+03	2.7E+03		

14.39262	811.4871125	G-Rg6	G-Rg6	1.0E+04	1.2E+04	5.6E+03	1.9E+04
14.39411	765.4814008	G-F4	G-F4	4.1E+03	7.8E+03	2.1E+03	1.5E+04
14.46318	797.4706362	DHDXG	DHDXG	5.5E+03	4.2E+03	3.8E+03	2.8E+03
14.4954	807.4543862	CS-Iva methyl ester	CS-Iva methyl ester	6.6E+03	5.9E+03	5.7E+03	8.5E+03
14.5	725.4486	PPT1-glc-acetyl	β-D-Glucopyranoside, (3β,6α, 12β,20S)-6-(acetyloxy)-3,12-dihydroxydammar-24-en-20-yl isomer	2.0E+03	1.4E+03		2.0E+03
14.65	1115.6338	PPD1-glc-glc-glc-octenoyl	octenoyl-Rd	1.3E+04	1.5E+04		
14.68	1059.577	PPD1-glc-glc-glc-butenoyl	Quinquenoside I isomer	1.7E+05	2.5E+05	3.7E+04	5.7E+04
14.68	975.5552	rha-PPD1-glc-glc	Gypenoside X isomer	2.5E+04	2.3E+04	3.0E+04	2.0E+04
14.68	811.4877	G-Rg6 isomer	G-Rg6 isomer	2.1E+04	2.1E+04	9.3E+03	3.7E+04
14.72	825.4591	OA-glc-glc	Erythrosaponin B	1.0E+03			2.0E+03
14.78	799.4822	PPT1-xyl/ara-rha	PPTXR2	3.2E+03		4.4E+03	2.2E+03
14.83463	665.4286204	G-Rh4	G-Rh4	6.3E+03	4.8E+03	5.5E+03	1.4E+04
14.83463	665.4286204	G-Rk3	G-Rk3	6.3E+03	4.8E+03	5.5E+03	1.4E+04
14.84	827.4788	PPT2-glc-glc	G-Rh15/isomer	4.9E+03	2.0E+03	5.5E+03	3.7E+03
14.91	1059.5739	PPD1-glc-glc-glc-butenoyl	Quinquenoside I isomer	7.6E+03	1.2E+04	2.8E+03	3.5E+03
14.92	945.5409	PPD1-glc-glc-glc	Rd isomer	4.8E+03	4.0E+03		3.6E+03
14.97999	783.4909738	G-F2	G-F2	1.7E+04	1.3E+04	1.4E+04	2.2E+04
15	969.5794	PPT1-glc-glc-octenoyl	octenoyl-Rg1	1.8E+04	1.2E+04	1.1E+03	1.9E+03
15.34	827.4784	PPT2-glc-glc	G-Rh15/isomer			7.9E+02	1.1E+03
15.39	1013.5701	PPD1-glc-glc-glc-butenoyl	Quinquenoside I isomer	3.5E+03	2.7E+04	1.0E+04	1.2E+04
15.51	1061.5785	PPD5-glc-glc-glc-butenoyl	H-butenoyl-Rd	2.0E+03	1.3E+04	5.0E+03	6.0E+03
15.6	1029.5647	PPD1-glc-glc-glc-acetyl-acetyl	Diacetyl-G-Rd isomer	1.3E+04	4.4E+03	1.7E+03	6.7E+03
15.7	681.4203	PPT3-glc	Rh5 isomer	8.7E+02	9.0E+02		

15.76	1231.6845	PPD1-4glc-octenoyl	quinquenoside II isomer	3.8E+04	4.2E+04	5.3E+03	2.3E+03
15.78	909.5574	PPT1-2glc-heptenoyl	heptenoyl-Rg1	2.0E+03	1.4E+03		3.1E+03
15.79	931.5279	PPD1-2xyl/ara-glc	Compound Ra1 isomer			1.4E+03	2.0E+04
15.88	1059.5734	PPD1-glc-glc-glc-butenoyl	Quinquenoside I isomer	5.3E+03	5.4E+03	1.9E+03	2.0E+03
15.9	827.477	PPT2-2glc	G-Rh15/isomer	1.4E+03		2.0E+03	1.1E+03
16.16	851.5094	PPD1-glc-glc-butenoyl	β-D-Glucopyranoside, (3β,12β)-3-(β-D-glucopyranosyloxy)-12-hydroxydammar-24-en-20-yl, 6-(2*E*)-2-butenoate isomer	2.3E+03	2.4E+03		2.6E+03
16.18	783.4925	20(R)-G-Rg3	20(R)-G-Rg3	6.9E+04	4.2E+04	3.4E+04	5.1E+04
16.18	783.4925	20(S)-G-Rg3	20(S)-G-Rg3	6.9E+04	4.2E+04	3.4E+04	5.1E+04
16.28	1029.564	PPD1-glc-glc-xyl/ara-butenoyl	butenoyl-CS-III	3.8E+03	7.1E+03	1.7E+03	2.7E+03
16.31	681.4227	PPT3-glc	Rh5 isomer			5.3E+02	
16.42	1201.678	PPD1-glc-glc-glc-xyl/ara-octenoyl	octenoyl-Rb2	1.9E+04	1.8E+04	2.5E+03	1.2E+03
16.46	829.4954	PPD1-glc-glc	F2 isomer	7.6E+03	5.0E+03	5.3E+03	1.2E+04
16.54	969.5763	PPT1-glc-glc-octenoyl	octenoyl-Rg1	2.8E+03	3.2E+03		
16.8	925.5497	PPT1-glc-xyl/ara-heptenoyl	heptenoyl-NG-R2	1.2E+03			
16.95	1201.6724	PPD1-glc-glc-glc-xyl/ara-octenoyl	octenoyl-Rb2	2.4E+04	2.2E+04	3.3E+03	6.0E+02
16.97	459.3845	DEDT	DEDT	1.7E+04	1.8E+04	1.4E+04	3.2E+04
17.01429	475.3787095	20(R)-PPT1	20(R)-PPT1	2.3E+03	1.1E+03	6.3E+02	2.1E+03
17.01429	475.3787095	20(S)-PPT1	20(S)-PPT1	2.3E+03	1.1E+03	6.3E+02	2.1E+03
17.08	969.5774	PPT1-glc-glc-octenoyl	octenoyl-Rg1	8.5E+03	7.4E+03	1.2E+03	1.9E+03
17.18	799.4847	PPD1-xyl/ara-glc	G-C-Y isomer	1.7E+04	1.4E+04	6.6E+03	2.2E+04
17.25	923.5729	PPT1-glc-glc-octenoyl	octenoyl-Rg1	1.4E+03	2.1E+03	9.1E+02	1.5E+03

17.3	1101.646	PPD1-glc-glc-glc-heptenoyl	heptenoyl-Rd	7.4E+03	6.6E+03	3.3E+03	2.3E+03
17.35	827.4803	PPT2-glc-glc	G-Rh15/isomer	5.5E+03	3.5E+03	3.6E+03	4.3E+03
17.55	799.4844	PPD1-glc-xyl/ara	G-C-Y isomer	2.7E+04	1.8E+04	7.9E+03	1.7E+03
17.7	871.5045	PPD1-glc-glc-acetyl	Rs3 isomer	2.6E+03	2.3E+03		1.5E+03
18.19	939.5674	PPT1-glc-xyl/ara-octenoyl	octenoyl-Rg1	6.1E+03	4.8E+03		
18.33	871.5073	20(R)-G-Rs3	20(R)-G-Rs3	7.0E+03	6.1E+03	2.0E+03	8.9E+03
18.33	871.5073	20(S)-G-Rs3	20(S)-G-Rs3	7.0E+03	6.1E+03	2.0E+03	8.9E+03
18.44077	807.4955	Rs4	Rs4	9.4E+02	1.4E+03	7.9E+02	1.5E+03
18.44077	807.4548277	ZB-RI-6‘ methyl ester	ZB-RI-6‘ methyl ester	9.4E+02	1.4E+03	7.9E+02	1.5E+03
18.45	797.4658	PPT2-glc-xyl/ara	noto-T5 isomer	3.0E+03	2.4E+03	2.6E+03	3.1E+03
18.89	1115.6364	PPD1-glc-glc-glc-octenoyl	octenoyl-Rd	3.1E+04	2.4E+04	2.7E+03	1.8E+03
19.06	663.4096	OA-glc	Oleanolic acid-28-O-β-D-glucopyranoside/isomer	9.9E+03	8.4E+03	6.4E+03	9.8E+03
19.07	665.4161	PPT2-glc	G-Rh4 isomer	1.7E+03	1.4E+03	1.1E+03	9.1E+02
19.9	667.4459	G-C-K	G-C-K	1.1E+03	1.5E+04	3.9E+03	1.1E+03
19.93165	765.4815319	G-Rg5	G-Rg5	1.5E+04	1.0E+04	6.5E+03	2.5E+04
20.19	851.5149	PPD1-glc-glc-butenoyl	β-D-Glucopyranoside, (3β,12β)-3-(β-D-glucopyranosyloxy)-12-hydroxydammar-24-en-20-yl, 6-(2*E*)-2-butenoate	1.8E+03	3.4E+03		3.2E+03
20.89	1105.6061	PPD1-glc-glc-glc-octenoyl	octenoyl-Rd	3.0E+04	5.0E+03	7.8E+02	2.0E+03
21.0154	621.4371481	20(S)-G-Rh2	20(S)-G-Rh2	1.7E+03	1.3E+03	8.8E+02	1.3E+03
21.0154	621.4371481	20(S)-G-Rh2	20(S)-G-Rh2	1.7E+03	1.3E+03	8.8E+02	1.3E+03
21.29	665.4263	PPT2-glc	G-Rh4 isomer	5.3E+03	4.4E+03	7.5E+03	3.4E+03
21.46	1115.6369	PPD1-glc-glc-glc-octenoyl	octenoyl-Rd	2.4E+03	1.3E+03		
21.68	459.3819	DDT	DDT	7.8E+03	4.6E+03	2.2E+03	4.8E+03

21.90833	807.490468	PPD2-2glc-acetyl	Rs4 isomer	1.9E+03	2.3E+03	1.2E+03	2.2E+03
23.71872	649.4336762	G-Rh3	G-Rh3	1.2E+03	9.5E+02	1.3E+03	1.2E+03
23.71872	649.4336762	G-Rk2	G-Rk2	1.2E+03	9.5E+02	1.3E+03	1.2E+03
25.54278	505.3890686	20(S)-PPD1	20(S)-PPD1	7.2E+02	5.9E+02	7.3E+02	1.1E+03
25.54278	505.3890686	20(S)-PPD1	20(S)-PPD1	7.2E+02	5.9E+02	7.3E+02	1.1E+03

Table S5

Batch information of *P. ginseng.*

Code no	Location	Batch No.	Age (years)	category
B1-1	Chang bai, Jilin Province	PY05CE0003	5	Ginseng cultivated 41°41′ N-42°25′ N, 127°42′ E-128°16′ E
B1-2		PY05CESB0008		
B1-3		PY05CS0003		
B2-1	Chang bai, Jilin Province	PN05CE0010		Ginseng cultivated 41°41′ N-42°25′ N, 127°42′ E-128°16′ E
B2-2		PN05CL0007		
B2-3		PN05CT0006		
B3-1	Lu dian, Yunnan Province	PY05YL0104		Ginseng cultivated 26°59′ N-27°32′ N, 103°09′ E-103°40′ E
B3-2		PY05YL0205		
B3-3		PY05YL0303		
B4-1	Hubei Province	PN05HB0105		Ginseng cultivated 29°01′ N-33°06′ N, 108°21′ E-116°07′ E
B4-2		PN05HB0207		
B4-3		PN05HB0302		

Table S6

Sources and types of the 63 ginsenosides reference.

No.	Name	Type	Source
1	Re3	PPT	our research group
2	20-glu-Rf	PPT	our research group
3	Re4	PPT	our research group
4	Re2	PPT	our research group
5	NG-R1	PPT	our research group
6	Re1	PPT	our research group
7	Rg1	PPT	our research group
8	Re	PPT	our research group
9	Ro	OA	our research group
10	CS-IV	OA	Chengdu Pusi Biotechnology Co., Ltd
11	CS-Iva	OA	Chengdu Pusi Biotechnology Co., Ltd
12	NG-R4	PPD	our research group
13	20(S)-Rf-1a	PPT	our research group
14	20(S)-Rf	PPT	our research group
15	20(R)-Rf	PPT	our research group
16	20(S)-NG-R2	PPT	our research group
17	F3	PPT	Chengdu Pusi Biotechnology Co., Ltd
18	Ra2	PPD	our research group
19	Ra3	PPD	our research group

20	20(R)-NG-R2	PPT	our research group
21	Rb1	PPD	our research group
22	20(S)-Rg2	PPT	our research group
23	20(R)-Rg2	PPT	our research group
24	Ra1	PPD	our research group
25	Rc	PPD	our research group
26	20(S)-Rh1	PPT	our research group
27	20(R)-Rh1	PPT	our research group
28	Rb2	PPD	our research group
29	Rb3	PPD	our research group
30	Q-R1	PPD	our research group
31	Rd	PPD	our research group
32	Rk1	PPD	our research group
33	Rs2	PPD	our research group
34	Rs1	PPD	our research group
35	Ro-methyl ester	OA	our research group
36	Rh19	PPT	our research group
37	DHDGG	PPD	our research group
38	Rg6	PPT	our research group
39	F4	PPT	our research group
40	DHDXG	PPT	our research group
41	CS-Iva methyl ester	OA	our research group
42	Rk3	PPT	our research group
43	Rh4	PPT	our research group
44	Rg9	PPT	our research group
45	F2	PPD	our research group
46	20(S)-Rg3	PPD	our research group
47	20(R)-Rg3	PPD	our research group
48	DEDT	PPT	our research group
49	20(S)-PPT	PPT	our research group
50	20(R)-PPT	PPT	our research group
51	20(S)-Rs3	PPD	our research group
52	20(R)-Rs3	PPD	our research group
53	Rs4	PPD	our research group
54	ZB-RI-6' methyl ester	OA	our research group
55	Rg5	PPD	our research group
56	G-C-K	PPD	Chengdu Pusi Biotechnology Co., Ltd
57	20(S)-Rh2	PPD	our research group
58	20(R)-Rh2	PPD	our research group
59	DDT	PPT	our research group
60	Rh3	PPD	Chengdu Pusi Biotechnology Co., Ltd

61	Rk2	PPD	Chengdu Pusi Biotechnology Co., Ltd
62	20(S)-PPD	PPD	our research group
63	20(R)-PPD	PPD	our research group
